# Effectiveness and Safety of Nonvitamin K Oral Anticoagulants Rivaroxaban and Apixaban in Patients with Venous Thromboembolism: A Meta-Analysis of Real-World Studies

**DOI:** 10.1155/2022/2756682

**Published:** 2022-06-09

**Authors:** Olivia Wu, Stephen Morris, Torben Bjerregaard Larsen, Flemming Skjøth, Alex Evans, Kevin Bowrin, Piotr Wojciechowski, Wojciech Margas, Maria Huelsebeck

**Affiliations:** ^1^Health Economics and Health Technology Assessment, Institute of Health and Wellbeing, University of Glasgow, Glasgow G12 8RZ, UK; ^2^Primary Care Unit, Department of Public Health & Primary Care, University of Cambridge, Cambridge CB2 0SR, UK; ^3^Department of Cardiology, Aalborg University Hospital, Aalborg 9100, Denmark; ^4^Aalborg Thrombosis Research Unit, Department of Clinical Medicine, Faculty of Health, Aalborg University, Aalborg 9100, Denmark; ^5^Unit of Clinical Biostatistics, Aalborg University Hospital, Aalborg 9100, Denmark; ^6^Bayer Plc, Reading RG2 6AD, UK; ^7^Creativ-Ceutical, Krakow, Poland; ^8^Bayer AG, Berlin 13353, Germany

## Abstract

**Background:**

Rivaroxaban and apixaban are the most widely used nonvitamin K oral anticoagulants (NOACs) in patients with venous thromboembolism (VTE). This meta-analysis evaluates the effectiveness and safety of both NOACs versus standard of care (SoC) in real-world practice.

**Methods:**

Real-world evidence (RWE) studies were identified through a systematic literature review conducted between January 2012 and July 2020, using Embase, MEDLINE, and the websites of cardiological, hematological, and oncological associations. Eligible RWE studies recruited adult patients with deep vein thrombosis and/or pulmonary embolism and presented a comparison between rivaroxaban and apixaban versus SoC, consisting either of vitamin K antagonists, heparins, or combinations thereof. Hazard ratios (HRs) for the comparison between NOACs and SoC were extracted from the relevant studies or estimated based on the reported binary data. The between-treatment contrasts were reported as HRs with associated 95% confidence intervals.

**Results:**

A total of 65 RWE studies were identified and considered relevant for the meta-analysis. Compared with SoC, both rivaroxaban and apixaban were associated with reduced risks of recurrent VTE and a lower rate of major bleeding events. Patients treated with rivaroxaban were at a lower risk of all-cause death compared with those receiving SoC (HR = 0.56 [0.39-0.80]), while evidence for apixaban from the identified studies was insufficient to demonstrate a statistically significant change in mortality (HR = 0.66 [0.30-1.47]).

**Conclusion:**

This analysis indicates that in real-world practice, rivaroxaban and apixaban are associated with a lower risk of recurrent VTE and major bleeding events compared with SoC. Survival benefit in patients treated with rivaroxaban was also observed.

## 1. Introduction

Venous thromboembolism (VTE), including deep vein thrombosis (DVT) and pulmonary embolism (PE), is the third most common cause of death from cardiovascular disease after heart attacks and stroke [1]. The reported annual incidence of VTE ranges from 1.04 to 1.83 per 1000 person-years in the overall population of people with European ancestry; however, it reaches 10 per 1000 person-years in the elderly [2]. DVT occurs when a blood clot is formed in the deep veins, usually in the large veins of the lower extremities or pelvis. It is associated with a higher risk of PE or postthrombotic syndrome. PE is a complication of DVT, which occurs when thrombi dislodge from clots in vein walls and travel through the heart to the pulmonary arteries [3]. PE is a serious life-threatening condition that may lead to pulmonary injury. It is considered a leading cause of sudden death if the circulation is blocked. Up to half of the patients with DVT will eventually develop long-term complications as a consequence of damaged valves in the blood vessels, which is referred to as postthrombotic syndrome [4]; this typically presents with swelling, pain, discoloration and, in severe cases, scaling or ulcers in the affected part of the body, predominantly the lower extremities.

One-third of patients with DVT or PE experience a recurrent episode within 10 years even in the absence of any acquired provoking risk factors underlying the first episode (referred to as an unprovoked event) [5, 6]. The traditional standard of care (SoC) for the prophylaxis of recurrent events includes a vitamin K antagonist (VKA) and/or heparins. However, VKAs are associated with an increased risk of major bleeding episodes, which occur in 1–3% of patients each year. Therefore, in the last decade, VKAs were replaced in the clinical practice with nonvitamin K oral anticoagulants (NOACs), which offer a superior risk–benefit profile [7–9]. The results of randomized controlled trials (RCTs) indicate that NOACs are as effective as VKAs, with a lower risk of major and life-threatening bleeding events, such as intracranial hemorrhage or gastrointestinal bleeding [10]. Additionally, NOACs do not require frequent monitoring and dose adjustment, or dietary restrictions; thus, they may be associated with a lower burden for patients. Therefore, NOACs are recommended over VKAs for the treatment of patients with VTE because they are associated with a favorable risk–benefit ratio. Warfarin is the preferred option for patients who cannot be treated with NOACs [10].

Of the four NOACs approved in the treatment of patients with VTE, apixaban (API) and rivaroxaban (RIV) can be initiated immediately following the VTE event, while the remaining two (dabigatran and edoxaban) require bridging heparins. Therefore, RIV and API, which were first approved for the treatment of patients with VTE in 2011 and 2012, respectively, are the most commonly used in patients with VTE and are now considered to be drugs of choice in the prevention and treatment of VTE [7, 8, 11]. Since approval, a large body of evidence for the safety and effectiveness of RIV and API in real clinical practice has been collected. Real-world evidence (RWE) is associated with higher external validity compared with clinical trials in the target population, which often represent a controlled environment that provides high internal validity but can fail to reflect the true nature of clinical practice due to tight exclusion criteria. Thus, this analysis is aimed at assessing the real-world effectiveness of RIV and API, as the most frequently used NOACs, versus SoC by pooling the results from the published real-world studies using meta-analysis.

## 2. Materials and Methods

### 2.1. Systematic Literature Review

Relevant RWE studies were identified from a systematic literature review carried out on 15 July 2020 using Embase, MEDLINE, and the websites of cardiological, hematological, and oncological associations. Eligible RWE included any kind of observational studies as well as analyses based on hospital registries and health insurance databases, recruited adult patients with DVT and/or PE, and presented a comparison between API and RIV versus either VKAs, heparins (including low-molecular-weight heparins), or combinations thereof. Trials with experimental design were excluded from this analysis. RWEs published since 2012 were considered to be relevant, with no restrictions for geographical scope. Eligible studies reported data for at least one relevant efficacy and safety outcome, including all-cause mortality, cardiovascular death, stroke, systemic embolism, myocardial infarction, transient ischemic attack, recurrent DVT, recurrent PE, and recurrent VTE events. The studies reporting data for pooled NOACs, single-arm studies, and papers not reporting relevant outcomes were excluded. Study selection was conducted by two analysts working independently, and any discrepancies were resolved by a third analyst. Data from studies meeting all inclusion criteria were extracted by one analyst and the correctness of the extraction was thoroughly checked by the second analyst.

Quality assessment was performed using a modified Downs and Black questionnaire, which was developed to evaluate both RCTs and noncontrolled trials [12]. The Downs and Black questionnaire is composed of 27 questions assessing study reporting (10 questions), external validity (3 questions), internal validity (13 questions), and power (1 question), with the maximum achievable score of 28 points (Supplementary Table 3). Since RCTs and extensions of RCTs were excluded in this review, the questions regarding study blinding, randomization, and power calculation were omitted. Therefore, the maximum achievable score was 25. Quality levels were determined according to Downs and Black score ranges: good (20–25), fair (15–19), and poor (≤14) [13].

### 2.2. Data Synthesis

#### 2.2.1. Study Overlapping

RWE studies often reported data from the same or overlapping sources of medical information, which could bias the results of the meta-analysis by duplication or multiplication of data from the same patients. An overlapping analysis was, therefore, conducted to identify such studies and to select unique estimates, in order to minimize the risk of patients' duplication in the meta-analysis (Supplementary Table 9). Two analysts working independently extracted information regarding the source of medical information and the period in which it was collected in each study. This data was then compared across all identified studies. Where overlapping estimates were identified, the one with the smallest variance was included in the meta-analysis.

#### 2.2.2. Data Aggregation

Hazard ratios (HRs) for the comparison between both NOACs and SoC were extracted from the relevant studies. If not reported, HRs were estimated either from incidence rates or binary data [14]. The natural logarithms of HRs for the between-treatment comparison and the corresponding standard errors were used as inputs for the meta-analysis. Estimates from relevant, unique (nonoverlapping) studies were pooled together using a random-effects meta-analysis with inverse variance weighting. Between-study heterogeneity was assessed using Cochran's *Q* test and the *I*^2^ index, which was interpreted as the proportion of true effect variance in relation to the whole observed variance [15]. In this analysis, we considered heterogeneity as substantial either when Cochran's *Q* test resulted in *p* < 0.10 or *I*^2^ > 50%.

Publication bias was assessed by visual inspection of funnel plot asymmetry and with the use of Egger's tests [16]. As recommended by the Cochrane Handbook [17], the analysis was conducted for meta-analyses with at least 10 studies with *p* value < 0.05 indicating asymmetry of funnel plot and the risk for publication bias. In the case of significant funnel plot asymmetry, the potential impact of bias on the estimates was assessed with two approaches. With the first approach, outliers were identified based on 95% confidence intervals, so that studies, in which 95% confidence intervals did not overlap with the confidence intervals of the pooled effect were considered outliers. Identified outliers were removed, and the meta-analysis was rerun [18]. With the second approach, a trim and fill method was used to impute estimates in order to restore the symmetry of funnel plots, followed by meta-analysis pooling both clinical data and imputed estimates [19].

All computations were carried out using the R statistical software version 4.0.3 using the *metafor* package.

#### 2.2.3. Scenarios

Several scenarios for the meta-analysis were considered to assess the robustness of data given various sets of comparators and the clinical effects in different follow-up times. The base case scenario adopted the broadest inclusion criteria that allow pooling of all unique studies. It included studies of various durations of follow-up, comparing RIV or API versus SoC. When individual studies reported estimates calculated at several durations of follow-up, the follow-up with the smallest associated variance was included. Finally, studies reporting HRs and those for which HRs were estimated were included in the base case.

The scenarios were conducted with modified assumptions of the base case, such as limiting the number of studies to those

- Reporting HRs directly for the relevant outcomes (scenario 1)
- With a reference group comprising VKA-based regimens (scenario 2)
- With a reference group comprising VKAs and heparin at any stage of the treatment (scenario 3)
- Reporting estimates relevant for the treatment duration ≤ 6 months (scenario 4)
- With estimates relevant for the treatment duration > 6 months (scenario 5)
- With estimates relevant for the treatment duration > 6 months and with the reference group comprising VKAs (scenario 6)

Included scenarios for the VTE population are presented in Supplementary Figure 1.

## 3. Results

### 3.1. Systematic Literature Review

A total of 10,801 articles were identified, of which 10,263 records were excluded after the deduplication process and abstract screening, and 369 were considered not to be relevant during a full-text analysis. Overall, 72 publications describing 63 studies that were focused on the VTE population were included. Additional two studies that recruited patients with VTE as well as with VTE and associated cancer were included (Supplementary Figure 2).

The quality of reporting varied between identified publications and a substantial proportion of them presented limited information regarding patients' sociodemographic factors and/or disease characteristics (Supplementary Tables 1-2). The number of patients observed in respective studies varied from 50 to 83,985 patients; however, the total number of participants in all studies was not possible to estimate due to significant overlapping of databases. Most studies reporting the type of VTE included patients diagnosed with both: DVT and/or PE; however, in six and five studies, the population consisted solely of patients with DVT and PE, respectively. The distribution of the type of VTE was not adequately described in nearly half of the papers. Overview of included studies and references are provided in the supplementary appendix.

The modified Downs and Black questionnaire scores ranged from 11 to 21 with 10, 48, and 7 reports assessed as being of good, fair, and poor quality, respectively (Supplementary Figure 3). Downs and Black score ranges were given corresponding quality levels as previously reported by Hooper et al. [13]. Most frequently points were subtracted for inadequate descriptions of the distribution of principal confounders in each group (Q5, 16 studies), the number of patients lost to follow-up (Q9, 22 studies), probability values for main outcomes (Q10, 18 studies), different time periods of outcome assessment between groups (Q17, 12 studies), and lack of adjustment for the confounding in the analysis of main findings (Q25, 10 studies) (Supplementary Tables 4-8).

### 3.2. Recurrent Thromboembolic Events

#### 3.2.1. Recurrent VTE

**Figure 1:**
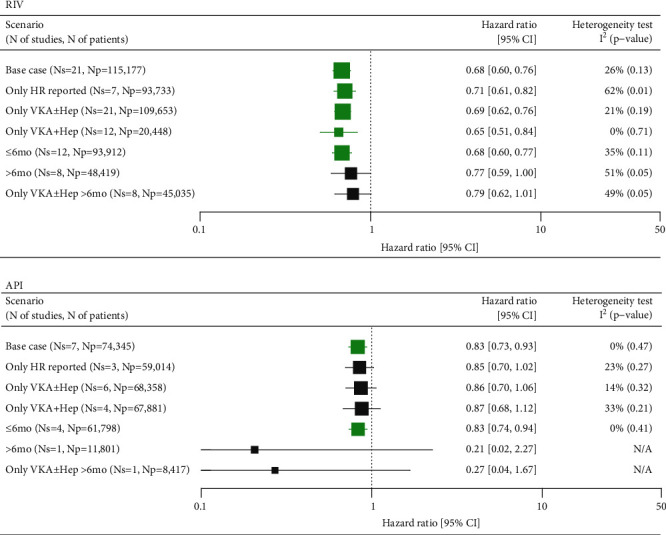
Summary of meta-analyses comparing RIV and API versus SoC in VTE for recurrent VTE. API: apixaban; CI: confidence interval; HR: hazard ratio; Np: number of patients; Ns: number of studies; RIV: rivaroxaban; SoC: standard of care; VKA±Hep: vitamin K antagonist±heparins; VTE: venous thromboembolism; N/A: not applicable. Green marks represent results indicating statistically lower event rate in the NOAC groups.

Twenty-one studies assessing more than 115,000 patients were included for the base case comparison between RIV versus SoC, whereas API was assessed in seven studies encompassing 74,345 patients. Both RIV and API were associated with a significantly reduced risk of recurrent VTE compared with SoC of 32% and 17%, respectively (HR = 0.68 (0.60, 0.76) for RIV and HR = 0.83 (0.73, 0.93) for API) (Supplementary Figures 4 and 12, Supplementary Tables 10 and 18). No significant heterogeneity was detected in either of the analyses (Figure 1).

The outcomes from the sensitivity analyses for the comparison between RIV and SoC were consistent with the base case scenario, showing a 21-32% lower risk of recurrent VTE in patients treated with RIV. Similarly, API compared with SoC presented a trend towards a 13-17% lower risk of VTE across majority of sensitivity analyses, except for the two scenarios assessing the risk of events within the follow-up periods exceeding 6 months. Both scenarios were informed by single studies conducted on a limited number of patients, therefore, the results were highly uncertain (Figure 1).

No evidence of publication bias was found for either scenario of the meta-analysis (Supplementary Figures 18-21).

#### 3.2.2. Recurrent PE

**Figure 2:**
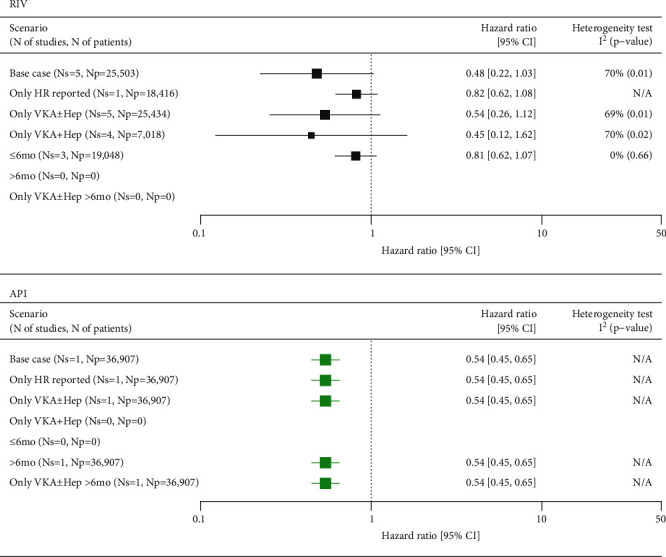
Summary of meta-analyses comparing RIV and API versus SoC in VTE for recurrent PE. API: apixaban; CI: confidence interval; HR: hazard ratio; Np: number of patients; Ns: number of studies; RIV: rivaroxaban; SoC: standard of care; VKA±Hep: vitamin K antagonist±heparins; VTE: venous thromboembolism; N/A: not applicable. Green marks represent results indicating statistically lower event rate in the NOAC groups.

The risk of PE was reported in five studies (Supplementary Figure 5, Supplementary Tables 11 and 19) assessing RIV versus SoC, which included a total of 25,503 patients. Of those, one trial reported a population-adjusted HR for between-treatment comparison, whereas between-group comparisons were made for the remaining trials. A random-effects meta-analysis of all studies showed a strong trend towards reduced risk of recurrent PE in the RIV group, although the result was imprecise due to a high level of heterogeneity (*I*^2^ = 70%). Likewise, the 95% confidence intervals crossed 1, thus indicating a lack of statistical significance (HR = 0.48 (0.22, 1.03)). The reasons for heterogeneity were investigated through a comparison of baseline characteristics of the included studies with a special emphasis on the proportion of patients with the provoked disease. However, the heterogeneity could not be explained by the reported differences in baseline characteristics between studies and was likely linked to the limited credibility of the estimated HRs. The remaining scenarios consistently presented HRs that were favorable for RIV, although without statistical significance (Figure 2).

One study encompassing nearly 37,000 patients compared API versus SoC regarding the risk of recurrent PE, showing a significantly lower risk of recurrent PE (HR = 0.54 (0.45, 0.65)) in favor of API.

#### 3.2.3. Recurrent DVT

**Figure 3:**
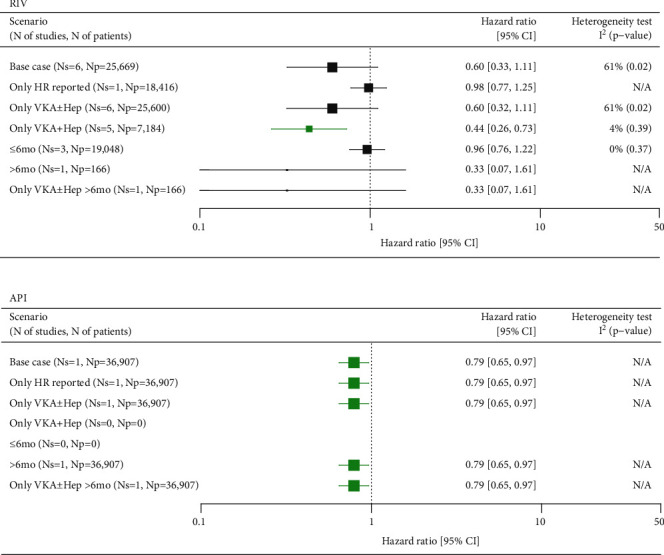
Summary of meta-analyses comparing RIV and API versus SoC in VTE for the recurrent DVT. API: apixaban; CI: confidence interval; DVT: deep vein thrombosis; HR: hazard ratio; Np: number of patients; Ns: number of studies; RIV: rivaroxaban; SoC: standard of care; VKA±Hep: vitamin K antagonist±heparins; VTE: venous thromboembolism; N/A: not applicable. Green marks represent results indicating statistically lower event rate in the NOAC groups.

The risk of recurrent DVT was reported in six studies (Supplementary Figure 6, Supplementary Tables 12 and 20) assessing RIV versus SoC, which together included a total of 25,669 patients. Population-adjusted HRs were reported in one study, whereas between-group comparisons were made for the other trials. A random-effects meta-analysis of all studies showed a strong trend towards reduced risk of recurrent DVT in the RIV group, although the result was imprecise due to a high level of heterogeneity (*I*^2^ = 61%). Likewise, 95% confidence intervals crossed 1, thus indicating a lack of statistical significance (HR = 0.60 (0.33, 1.11)). The heterogeneity could not be explained by the differences in baseline characteristics between studies and was likely linked to the limited credibility of HRs, which were estimated from binary data for five out of six studies included in the meta-analysis. In the remaining study, the result of the comparison could be biased by a significantly higher proportion of the patients on RIV who had a history of VTE, bleeding, and cardiovascular events [20]. A scenario pooling studies with VKA and heparins as reference presented a significantly lower risk of PE in the RIV group (HR = 0.44 (0.26, 0.73)) (Figure 3). In this analysis, the largest population-based cohort was excluded as the use of bridging heparins was not described.

One study encompassing nearly 37,000 patients demonstrated a 21% reduced risk of recurrent DVT in patients receiving API compared with SoC (HR = 0.79 (0.65, 0.97)) (Figure 3).

### 3.3. All-Cause Mortality

**Figure 4:**
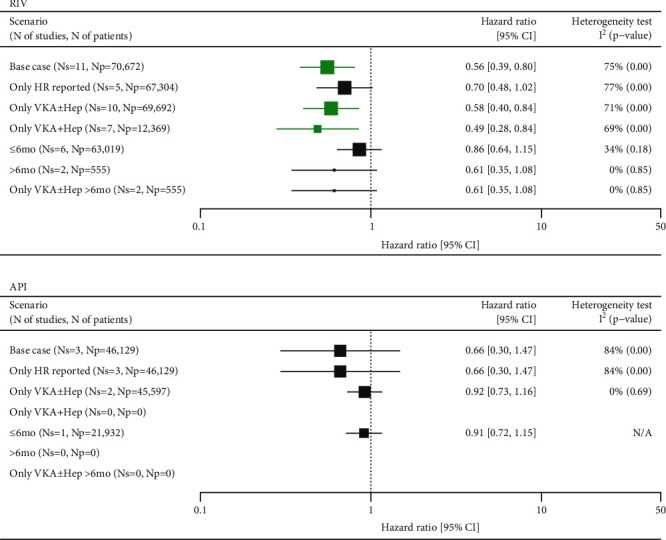
Summary of meta-analyses comparing RIV and API versus SoC in VTE for all-cause mortality. API: apixaban; CI: confidence interval; HR: hazard ratio; Np: number of patients; Ns: number of studies; RIV: rivaroxaban; SoC: standard of care; VKA±Hep: vitamin K antagonist±heparins; VTE: venous thromboembolism; N/A: not applicable. Green marks represent results indicating statistically lower event rate in the NOAC groups.

The risk of death was assessed in a total of 11 studies assessing RIV (*n* = 70,672 patients) and three studies assessing API (*n* = 46,129). A meta-analysis of all studies revealed that RIV was associated with a significantly lower risk of death compared with SoC (HR = 0.56 (0.39, 0.80)) (Supplementary Figures 7 and 13, Supplementary Tables 13 and 21), which was confirmed in scenarios including studies with VKA-based regimens as a reference treatment. Trends favoring RIV over SoC were observed in the subset of studies reporting population-adjusted HRs and in the subgroups of studies assessing either short-term or long-term therapy, although the results were not significant (Figure 4).

Meta-analysis of studies assessing API did not demonstrate significant differences versus SoC in either of the feasible scenarios but point estimates in favor of API (Figure 4).

Noticeable heterogeneity was detected in base-case meta-analyses for both RIV (*I*^2^ = 75%) and API (*I*^2^ = 84%). Visual inspection of the forest plots and analysis of baseline characteristics indicated that the data collected by Wysokinski et al. contributed to the between-trial heterogeneity [21]. This study compared RIV and API versus low-molecular-weight heparins and the proportions of patients with cancer in the NOAC groups were half of those of patients treated with heparins, thus favoring RIV and API. Wysokinski et al. was not eligible for the meta-analysis of studies comparing NOACs versus VKA-based therapies; the results of which were, however, consistent with the base case analyses (Figure 4) [21].

Potential asymmetry of funnel plots was revealed for the base case (*p* = 0.020) and scenario 2 (*p* = 0.025) for the comparison of RIV vs. SoC. In the base case, one study (Roetker 2018) was identified as an outlier. When excluded, the result of the meta-analysis indicated a significantly lower risk of RIV vs. SoC (HR = 0.50 (0.34, 0.73)). The alternative trim and fill method added 5 data points which changed the result, although the point estimate remained in favour of RIV (HR = 0.89 (0.62, 1.28)) (Supplementary Figures 22-26).

The analysis of scenario 2 did not reveal outliers and the hazard ratio following the addition of 5 data points using the trim and fill method did not indicate significant differences between RIV and SoC, although the point estimate was in favor of RIV (HR = 0.89 (0.62, 1.28)) [19].

### 3.4. Bleeding Events

#### 3.4.1. Major Bleeding

**Figure 5:**
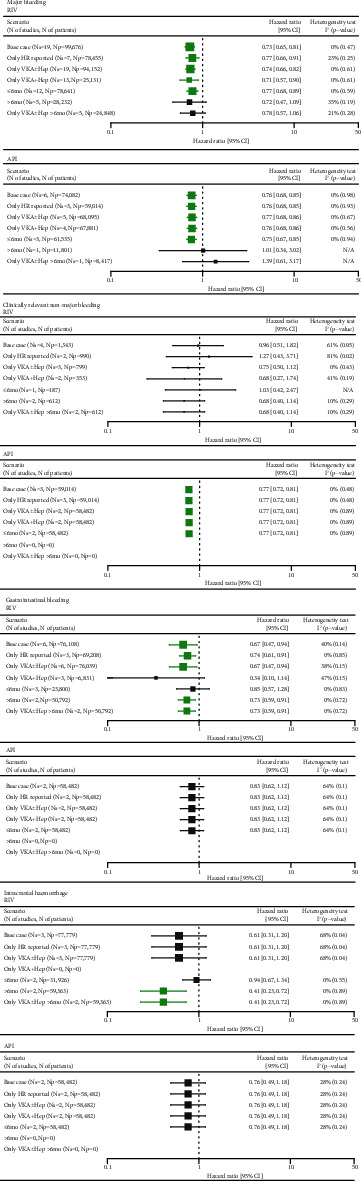
Summary of meta-analyses comparing RIV and API versus SoC in VTE for bleeding events. API: apixaban; CI: confidence interval; HR: hazard ratio; Np: number of patients; Ns: number of studies; RIV: rivaroxaban; SoC: standard of care; VKA±Hep: vitamin K antagonist±heparins; VTE: venous thromboembolism; N/A: not applicable. Green marks represent results indicating statistically lower event rate in the NOAC groups.

Meta-analyses of 19 RWE studies assessing RIV (*n* = 99,676) and six studies assessing API (*n* = 74,082) demonstrated that both NOACs are associated with a significantly lower risk of major bleeding compared with SoC (HR = 0.73 (0.65, 0.81) for RIV and HR = 0.76 (0.68, 0.85) for API) (Supplementary Figures 8 and 14, Supplementary Tables 14 and 22). These results were consistent across all scenarios except meta-analyses of the subset of studies assessing long-term treatment, for which there were no significant differences between NOACs and SoC, although the trend in favor of RIV versus SoC was maintained. No significant between-study heterogeneity was detected for either analysis (Figure 5).

No evidence of publication bias was found for either scenario of the meta-analysis for major bleeding (Supplementary Figures 27-30).

#### 3.4.2. Clinically Relevant Nonmajor Bleeding (CRNMB)

Four studies encompassing 1,343 patients for RIV and three studies encompassing 59,014 patients for API were included for the meta-analysis. The meta-analysis did not demonstrate significant differences between RIV and SoC in either scenario, whereas API was associated with a significantly (23%) lower risk of CRNMB versus SoC across all feasible scenarios (Supplementary Figures 9 and 15, Supplementary Tables 15 and 23). Significant heterogeneity was observed in meta-analyses assessing RIV based on studies with various comparators but not in scenarios including studies comparing with VKA-based regimens (Figure 5).

#### 3.4.3. Gastrointestinal (GI) Bleeds

Meta-analysis of six RWE studies encompassing 76,108 patients showed that RIV compared with VKAs was associated with a significantly reduced risk of GI bleeds by 26–33% across respective scenarios (Supplementary Figures 10 and 16, Supplementary Tables 16 and 24). In two scenarios, including studies with a reference group comprising VKAs (scenario 3) and short-term treatment (scenario 4), the results did not reach significance, although point estimates were in favor of RIV. Meta-analysis of two studies for API encompassing 58,482 patients did not demonstrate significant differences between API and SoC regarding the risk of GI bleeds across all scenarios. There was no evidence for significant between-study heterogeneity across the meta-analyses (Figure 5).

#### 3.4.4. Intracranial Hemorrhage

Three and two studies encompassing 77,779 and 58,482 patients were included for the meta-analysis for RIV and API, respectively (Supplementary Figures 11 and 17, Supplementary Tables 17 and 25). In all feasible scenarios, RIV was associated with a lower risk of intracranial hemorrhage compared with SoC; however, the statistical significance level was reached only for the scenarios with long-term treatment.

The meta-analysis did not reveal a significant difference between API and SoC regarding intracranial hemorrhage (Figure 5).

Significant heterogeneity was revealed between three trials assessing RIV, which could not be explained by differences in baseline populations (*I*^2^ = 68).

## 4. Discussion

The efficacy and safety of NOACs have been extensively studied in RCTs, which have paved the way for positive clinical recommendations in the treatment of patients with VTE and continuous replacement of the traditional VKA-based therapy. Of the four NOACs, RIV and API are preferred by the National Institute for Health and Care Excellence over dabigatran and edoxaban in patients with confirmed proximal DVT and PE [7, 8, 11, 22]. This reflects in the availability of real-world clinical data, which are most abundant for RIV and API. Therefore, this analysis was focused on the assessment of the real-world effectiveness and safety of the two drugs as representatives of the NOACs.

Although RCTs are considered to be the cornerstone for the assessment of clinical efficacy and safety of therapies, the role of observational studies cannot be underestimated. Despite RWE studies being associated with an elevated risk of bias compared with RCTs in establishing comparative effectiveness, the importance of this type of evidence is growing. This is particularly the case because RWE studies often provide information for broader and more diverse populations compared with clinical trials, which adopt tight inclusion criteria, to exclude the participation of patients with a range of concomitant diseases or high-risk profiles. Additionally, the experimental conditions within clinical trials, including regular and frequent visits or tight monitoring of adherence, cause the trial settings to drift away from real-world clinical practice. Therefore, although RCTs often present results, which are more consistent and easier to interpret compared to those from RWE studies, external credibility may be higher for RWE [23]. Additionally, large RWE studies may also supplement experimental studies in the assessment of rare clinical outcomes, which could not in practice be assessed in experimental studies due to inadequate power.

The results of this analysis are consistent with the outcomes of the existing clinical trials, which demonstrated that RIV and API have favorable risk–benefit profiles compared with SoC. The two pivotal RCTs EINSTEIN DVT and EINSTEIN PE showed noninferiority of RIV compared with VKA-based regimens regarding the risk of recurrent VTE in patients with DVT and PE, respectively [24, 25]. The findings from the EINSTEIN DVT trial indicate a strong trend towards a lower risk of recurrent VTE in the RIV group (HR = 0.68 (0.44, 1.04)). However, this trial was not designed to demonstrate differences between groups; therefore, the statistical power was probably inadequate to demonstrate superiority [25]. Interestingly, our meta-analysis encompassing 115,177 patients indicates the same similar magnitude of risk reduction of recurrent VTE in the RIV groups but with clear significance (HR = 0.68 (0.60, 0.76)). The analysis of predefined secondary outcomes of the EINSTEIN DVT trial demonstrated the superiority of RIV over SoC regarding the net clinical benefit, defined as the composite of recurrent VTE and major bleeding events (HR = 0.67 (0.47, 0.95)), and showed a trend towards a lower risk of death (HR = 0.67 (0.44, 1.02)). Similarly, the base case scenario of the current meta-analysis, encompassing 70,672 patients, indicated that RIV was associated with a significant (44%) reduction in the risk of death compared with SoC (HR = 0.56 (0.39, 0.80)). Major bleedings in the EINSTEIN DVT trials were recorded less frequently in the RIV group compared with SoC; however, the between-group difference did not reach significance due to insufficient power (0.8% vs. 1.2%, HR = 0.65 (0.33, 1.30)). A significant reduction in the risk of major bleeding was, however, demonstrated in the second EINSTEIN trial that was conducted on patients with PE (HR = 0.49 (0.31, 0.79)). In the current meta-analysis encompassing nearly 100,000 patients, RIV was associated with a significant (27%) reduction in the risk of major bleeding (HR = 0.73 (0.65, 0.81)). Therefore, the meta-analysis of RWE studies does not only support the noninferiority of RIV over SoC in terms of clinical outcomes but also indicates that the use of RIV instead of SoC is associated with a lower risk of recurrent VTE, a better survival, and a lower chance for major bleeding and GI bleeding events in the real world. Consistently, this analysis also reconfirmed the outcomes of the pivotal trial comparing API versus conventional treatment in patients with DVT and PE (AMPLIFY), which demonstrated noninferiority of API versus SoC regarding the risk of recurrent VTE and superiority regarding the risk of major bleeding and CRNMB events. The AMPLIFY trial reported an HR of 0.84 for the risk of recurrent VTE, indicating a nonsignificant trend towards lower event risk in the API group, which was confirmed in this meta-analysis, which pooled data from 74,345 patients (HR = 0.83 (0.73, 0.93)). However, the results of scenarios including studies reporting adjusted HRs and the comparison versus VKA-based regimens did not reach significance for the difference between API and conventional treatment, possibly due to an insufficient sample for this magnitude of the effect. Neither the AMPLIFY trial nor our meta-analysis demonstrated a significant difference between API and SoC regarding mortality [26].

Consistent with the findings from the clinical trials, this analysis indicates that in real-world practice RIV and API are associated with a more favorable risk–benefit ratio compared with SoC. Therefore, the observations collected in real-world practice support the recent recommendations issued by the American Society of Haematology that favor the choice of NOACs in the management of patients with VTE [10]. Furthermore, the recommendations for the management of patients with VTE presented by NICE prefer RIV and API over other NOACs [22]. Nevertheless, none of the current guidelines discriminate between RIV and API since the populations recruited in the respective clinical trials were not fully comparable leading to uncertainty of the comparison, although attempts to compare effectiveness and safety between RIV and API in real-world practice were undertaken. The recently published results of an instrumental variable estimation adjusting for the facility preference for medication used in stroke prevention in Danish patients with atrial fibrillation indicated that RIV might be associated with a higher risk of major bleeding compared with API (relative risk 1.89 (95% CI, 1.06–2.72)) [27]. Yet, the credibility of this analysis might be severely limited since the essential assumptions made by the authors could not be verified in practice [28]. Therefore, an intermediate comparison between RIV and API based on the identified observational studies would likely produce biased estimates due to unmeasured confounders and, in consequence, have insufficient credibility to either support or update existing guidelines.

Real-world studies reflect clinical experience across a broad population of patients, although carries an inherent risk of confounding, due to the studies being nonrandomized. Moreover, the allocation to different treatments in the observational studies is likely associated with patients' clinical status—for example, patients with cancer are more likely to be treated with low-molecular-weight heparins. Important between-arm differences make the comparison difficult and likely lead to biased inference when the heterogeneity is not adequately accounted for. Some authors adopted statistical methods to adjust for between-arm differences; however, it should be considered that the methods of adjustment of HRs varied across studies. Although most of the RWE studies reporting HRs used adequate methods for the adjustment of between-arm heterogeneity, including propensity score matching, HRs were estimated based on binary data for a noticeable number of studies. This approach introduces uncertainty, as the estimated HRs were unadjusted for within-study heterogeneity. Therefore, to mitigate the risk of bias associated with the inherent heterogeneity of RWE, six additional scenarios were planned to allow for the assessment of the robustness of data in subgroups regarding: the credibility of estimates, comparators, and duration of treatment.

The results of our meta-analysis could also be influenced by the definitions of outcomes differing between individual studies and the lack of proper outcome adjudication. For instance, the definition of major bleeding was not adequately reported in the studies and thus could vary substantially between clinical centers, thus introducing uncertainty for interpretation and inference.

Inclusion of studies carried out on the same database can lead to the same patients being repeatedly included in the meta-analysis. To avoid methodological bias caused by a potential overlap in the study populations, several studies were excluded from the analysis due to identified database overlap. However, although a thorough overlapping analysis was conducted, this does not guarantee that patients analyzed in single-center studies were not already captured by large medical databases. Therefore, the risk of duplication of patients' information could not be entirely excluded. Additionally, the largest identified studies collected medical data from the USA healthcare system, which have the largest contribution in estimates from this meta-analysis.

Asymmetry of funnel plots was analyzed to detect potential bias in the results of meta-analyses. However, the important limitation of this regression-based analysis is that it is not recommended for meta-analyses pooling less than 10 studies [29]. Therefore, it was only applicable for selected comparisons between RIV and SoC and could not be tested in any of the meta-analyses comparing API versus SoC due to the scarcity of clinical data. There was no evidence of asymmetry of funnel plots in most of the meta-analyses pooling an adequate number of studies, with the exception of two scenarios comparing the RIV with SoC for all-cause mortality. However, the available methods for restoring the symmetry of funnel plots, which included the exclusion of outliers and imputation of ‘missing' estimates, did not change the trends of meta-analyses indicating a lower risk of death in patients receiving RIV.

The presence of asymmetry in forest plots should be interpreted with due caution since it may not only indicate publication bias but also may have other reasons as well as occur by chance [29]. Moreover, observational studies are inherently characterized by larger variability and higher number of confounding factors compared with randomized trials; therefore, publication bias is much less important in the context of real-world data [29, 30].

Therefore, the estimates of this meta-analysis may not represent the clinical practice in other regions of the world.

## 5. Conclusions

Conclusions should clearly explain the main findings and implications of the work, highlighting its importance and relevance.

## Data Availability

All data is included in the manuscript and Supplementary Information files.

## Supplementary Materials

Supplementary Materials Supplementary Materials include an overview of included studies with quality and bias assessment, description of scenarios of meta-analysis in VTE population, PRISMA flowchart, inputs and results of meta-analyses, and the assessment for asymmetry of funnel plots.
