# Supplementary material for: Effectiveness and Safety of Nonvitamin K Oral Anticoagulants Rivaroxaban and Apixaban in Patients with Venous Thromboembolism: A Meta-Analysis of Real-World Studies
Source: Cardiovasc Ther. 2022 Jun 9;2022:2756682. doi: 10.1155/2022/2756682 (PMC9203223; doi:10.1155/2022/2756682)
Supplement: Supplementary Materials — include an overview of included studies with quality and bias assessment, description of scenarios of meta-analysis in VTE population, PRISMA flowchart, inputs and results of meta-analyses, and the assessment for asymmetry of funnel plots. [file 2756682.f1.docx]

# Supplementary Appendix

Supplement to: Wu O., et al. Effectiveness and safety of non-vitamin K oral anticoagulants rivaroxaban and apixaban in patients with venous thromboembolism – a meta-analysis of real-world studies.

Olivia Wu^1^, Stephen Morris^2^, Torben Bjerregaard Larsen^3,4^, Flemming Skjøth^4,5^, Alex Evans^6^, Kevin Bowrin^6^, Piotr Wojciechowski^7^, Wojciech Margas^7^, and Maria Huelsebeck^8^

^1^ Health Economics and Health Technology Assessment, Institute of Health and Wellbeing, University of Glasgow, Glasgow G12 8RZ, UK

^2^ Primary Care Unit, Department of Public Health & Primary Care, University of Cambridge, Cambridge CB2 0SR, UK

^3^ Department of Cardiology, Aalborg University Hospital, Aalborg 9100, Denmark

^4^ Aalborg Thrombosis Research Unit, Department of Clinical Medicine, Faculty of Health, Aalborg University, Aalborg 9100, Denmark

^5^ Unit of Clinical Biostatistics, Aalborg University Hospital, Aalborg 9100, Denmark

^6^ Bayer Plc, Reading RG2 6AD, UK

^7^ Creativ-Ceutical, Krakow, Poland

^8^ Bayer AG, Berlin 13353, Germany

# Overview of included studies

A total of 65 identified studies reported outcomes relevant for the meta-analysis. They included cohorts of 50 to 83,985 patients.[1-65] Regarding disease indication, 50 studies were conducted on patients with VTE overall, six studies focused specifically on patients with DVT[1, 8, 23, 51, 57, 58], five studies focused on patients with PE[30, 52, 53, 59, 60], three studies focused on patients with VTE and atrial fibrillation[35, 47, 49], and one study focused on patients with portal vein thrombosis.[64] Most studies (n=39) were multicentre[1, 3, 4, 7, 10, 12-18, 20, 23, 26-29, 31, 32, 34, 37-41, 47, 49, 50, 54-56, 58-62, 65], 23 studies described the experience of single clinical centres[2, 5, 6, 8, 9, 21, 22, 24, 25, 30, 33, 35, 36, 42-46, 48, 51-53, 63], whereas the remaining three studies did not report information regarding the location.[19, 57, 64] The mean treatment duration ranged from 1 to 12 months.

After the overlapping analysis, the meta-analysis included 35 publications comparing NOACs versus VKA-based regimens. Of those studies, 15 reported results for VKAs and heparins at any stage of the treatment and 20 reported results for VKAs without any information regarding the use of heparins.

In addition, the meta-analysis included three studies reporting outcomes for NOACs versus heparin (without VKA) regimens and seven studies reporting outcomes for both RIV and API versus comparators. Identified RWE allowed for comparisons between NOACs and SoC for all relevant outcomes.

Supplementary figure 1 Scenarios of meta-analysis in VTE population


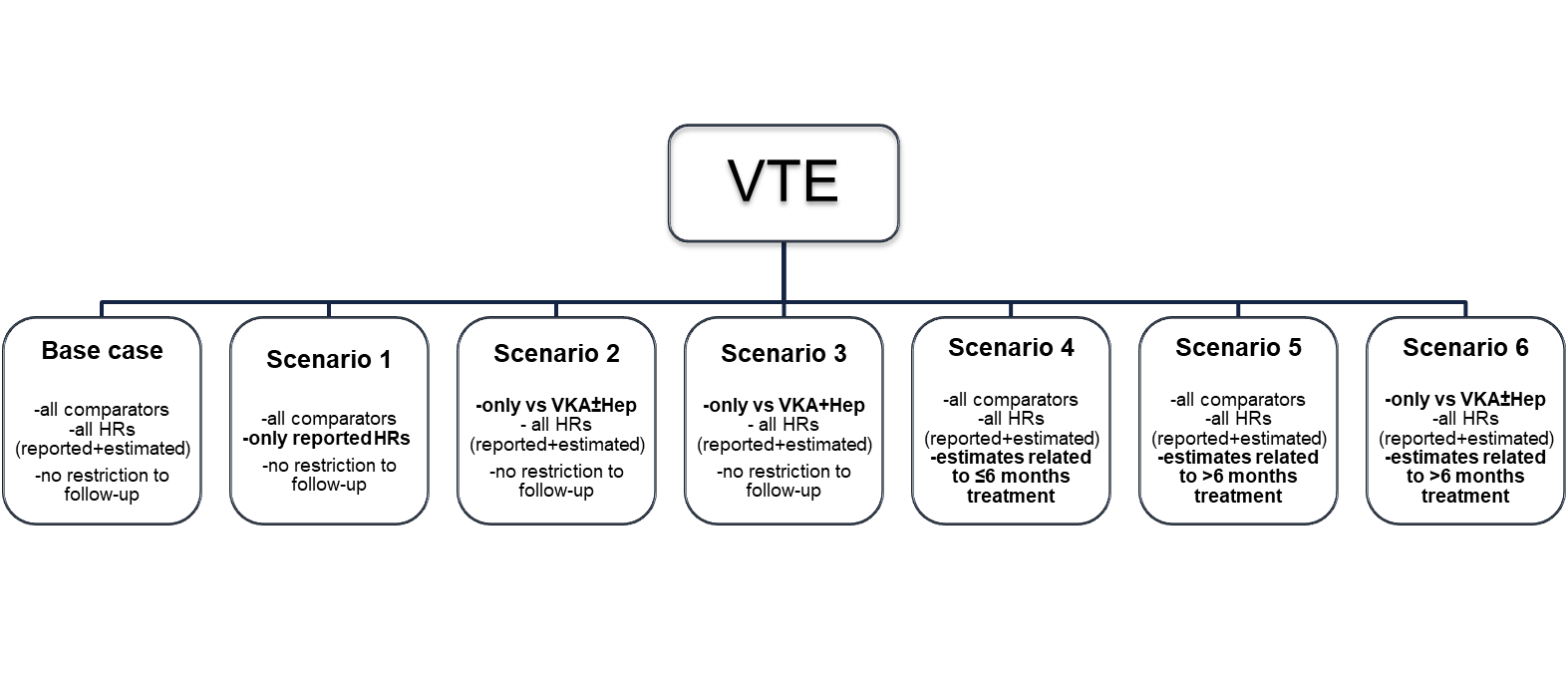


HR = Hazard ratio; VKA±Hep = vitamin K antagonist ±heparins; VTE = Venous thromboembolism.

Supplementary figure 2 PRISMA flowchart


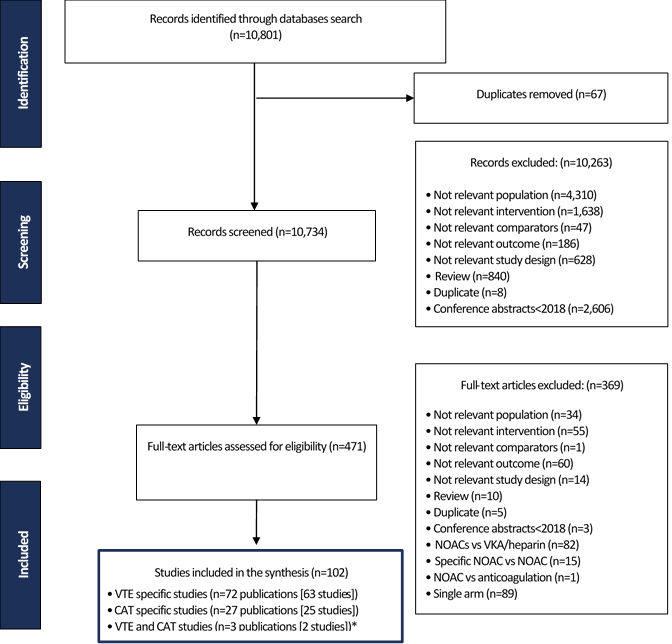


*: VTE and C-VTE (CAT) studies: Ageno, 2017; Coleman, 2017; Gaerter, 2017

NOAC = Non-vitamin K oral anticoagulant; VKA = vitamin K antagonist; VTE = Venous thromboembolism.

Supplementary table 1 Characteristics of SLR-identified studies

| **Author, year**  **(Acronym)** | **Country/ countries** | **Treatments** | **Type of study** | **Name of database** | **Time of recruitment** | **Population** | **Number of patients** | **Follow-up (months)** | **Treatment duration (months)** | **Newly vs. previously treated** |
| --- | --- | --- | --- | --- | --- | --- | --- | --- | --- | --- |
| Ageno, 2016[1] | International | RIV | Prospective Cohort study Multicentre | NR | 26^th^ Jun 2012- 31^st^ Mar 2014 | DVT | 4,768 | 239 (154–388) d ¥ | 184 (95-359) d ¥ | Previously |
|  |  | Standard therapy (LMWH, heparin /fondaparinux /VKA) |  |  |  |  |  | 284 (181–408) d ¥ | 190 (97-368) d ¥ |  |
| Badreldin, 2018[2] | USA | •API  •RIV  •EDO  •DAB  •WARF | Retrospective Chart review Single-centre | NR | Aug 2015- May 2017 | VTE | 441 | 3 and 6 | NR | Newly |
| Bouget, 2020[3] | France | •VKA  •API  •RIV  •DAB | Prospective  Cohort study Multicentre | French national health insurance database (SNIRAM) | 1^st^ Jan 2013- 31^st^ Dec 2015 | VTE | 47,469 | VKA: 234 (116-510) d ¥ | Duration of use: <6, 6-12 or >12 | Previously + Newly |
|  |  |  |  |  |  |  |  | HD NOAC: 163 (67-412) d ¥ |  |  |
|  |  |  |  |  |  |  |  | LD NOAC: 60 (60-119) d ¥ |  |  |
| Bounameaux, 2020[4] | International  (28 countries worldwide) | •VKA  •API  •RIV  •EDO  •DAB | Prospective Cohort study Multicentre | NR | May 2014- Jan 2017 | VTE | 7,987 | 12 | NR | Previously + Newly |
| Bryk, 2016[5] | Poland | •RIV  •VKA | Prospective Cohort study Single-centre | NR | May 2013- Jun 2016 | VTE | 121 | 13 ¥ | >3 | Not clear |
| Bui, 2019[6] | Vietnam | RIV | Prospective cohort and case control singl -centre | NR | Jan 2017- Aug 2018 | VTE | 187 | 6 | 167.6 (15.8) Ɣ | Not clear |
|  |  | Enoxaparin/ VKA |  |  |  |  |  |  | 169.2 (16.4) Ɣ |  |
| Carroll, 2018[7] | England | • API  • RIV  • WARF | Retrospective  Cohort study Multicentre | •Clinical Practice Research Datalink  •Hospital Episode Statistics | Jan 2013- Jul 2017 | VTE | 3,300 | 180 d | NR | Not clear |
| Chaudhari, 2019[8] | India | •RIV  •WARF | Prospective Cohort study Single-centre | NR | Jan 2017 -Dec 2017 | DVT | 139 | 6 | 6 | Previously |
| Chu, 2017[9] | USA | •RIV  •WARF /Enoxaparin | Retrospective  Chart review Single-centre | NR | 1^st^ Jan 2015 -Jan 31^st^ 2016 | VTE | 75 | NR | NR | Newly |
| Coleman, 2017[10] | USA | •RIV  •WARF | Retrospective  database analysis Multicentre | USA Truven MarketScan | Jan 2012 - Jun 2015 | VTE (Patients with  ≥6 months of continuous medical and prescription benefits prior to the index event) | 45,853 | 12 | NR | Newly |
| Coleman, 2017a [11] | USA | •RIV  •WARF | Retrospective Claim analysis Multicentre | USA Truven MarketScan | Jan 2012-Jun 2015 | VTE  (Patients with  ≥6 months of continuous medical and prescription benefits prior to and ≥4 moths follow-up after the index event) | 36,957 | 16 (9) Ɣ  Range: 4-39 | 7 Ɣ | Newly |
| Coleman, 2018b[12] | USA | •RIV  •WARF | Retrospective Claim analysis Multicentre | USA Truven MarketScan | Jan 2012- Dec 2016 | VTE  (frail patients) | 6,869 | 12 | 5 (6) Ɣ | Newly |
| Coleman, 2018[13] | USA | •RIV  •WARF | Retrospective Claim analysis Multicentre | USA Truven MarketScan | Jan 2012 - Dec 2016 | Provoked VTE | 17,618 | 12 | 91 d | Newly |
| Coleman, 2018c[14] | USA | •RIV  •WARF | Retrospective Claim analysis Multicentre | USA Truven MarketScan | Jan 2012 -Sep 2015 | VTE (PHSs) | 806 | 12 | 7 | Newly |
| Coleman, 2018d[15] | USA | •RIV | Retrospective Claim analysis Multicentre | USA Truven MarketScan | 1^st^ Jan 2012- 31^st^ Mar 2017 | VTE | 4,990 | 12 | >3 | Newly |
| Coleman, 2018a[16] | USA | •RIV  •WARF | Retrospective Claim analysis Multicentre | USA Truven MarketScan | 1^st^ Jan 2012 -31^st^ Dec 2016 | VTE | 47,342 | 3 and 6 | NR | Newly |
| Costa , 2020[17] | USA | •RIV  •WARF | Retrospective Cohort study | Optum® | 1^st^ Nov 2012 -30^th^ Sep 2018 | VTE  (African American patients) | 4,939 | 12 | NR | Newly |
| Costa , 2020a[18] | USA | •RIV  •WARF | Retrospective Cohort study Multicentre | Optum® | 1^st^ Nov 2012 -30^th^ Sep 2018 | Obese VTE | 13,510 | 12 | NR | Newly |
| Dawwas, 2018[19] | USA | •API  •WARF | Retrospective Cohort study | USA Truven MarketScan | Jan 2014 - Dec 2015 | VTE | 5,536 | NR | ≥12 | Newly |
| Dawwas, 2020[20] | USA | • API  • WARF | Retrospective Cohort analysis Multicentre | USA Truven MarketScan | Jan 2014 - Dec 2017 | VTE | 36,907 | NR | NR | Newly |
| De Crema, 2015[21] | Belgium | •RIV  •VKA | Retrospective Database analysis Single-centre | NR | Sep 2007- Sep 2014 | VTE | 119 | NR | NR | Not clear |
| Desai, 2016[22] | USA | •RIV  •WARF  •Enoxaparin | Retrospective Registry Single-centre | NR | Jan 2011- Jul 2014 | VTE | 414 | NR | NR | Newly |
| Ferreira, 2020[23] | Brazil | •RIV  •Enoxaparin/ WARF | Retrospective Cross-sectional Multicentre | NR | From 2015 to 2017 and from 2006 to 2016 | DVT | 129 | >15 (10–24) | 6 (4–7) ¥ | Previously |
|  |  |  |  |  |  |  |  | >61 (34–86.5) | 7 (6–12) ¥ |  |
| Fung, 2019[24] | China | •RIV  •WARF | Retrospective  Cohort study Single-centre | NR | 1^st^ Mar 2012 - 28^th^ Feb 2017 | VTE | 181 | 12 | NR | Newly |
| Gaertner, 2017[25] | USA | •RIV  •VKA  •Heparin /fondaparinux | Prospective Registry Single-centre | NR | Nov 2013 - Jul 2015 | VTE | 499 | 6 | 3 | Previously + Newly |
| Goldhaber, 2020[26] | USA | • VKA  • DAB | Retrospective Cohort study Multicentre | NR | NR | VTE | 2,969 | Up to 12 | 12 | Newly |
| Gollamudi, 2018[27] | USA | •API  •WARF | Retrospective Case-control study  Multicentre | Explorys Inc | NR | VTE | 9,100 | 90 d | NR | Previously |
| Guo, 2020[28] | USA | •API | Retrospective cohort study Multicentre | IQVIA PharMetrics | 1^st^ Sep 2014-30 Sep 2018 | VTE | 17, 716 | 6 | 103 d | Newly |
|  |  | •WARF |  |  |  |  |  |  | 109 d |  |
| Hlavacek, 2019[29] | USA | •API | Retrospective  Database analysis Multicentre | Center of Medicare & Medicaid Services (CMS) | 1^st^ Mar 2014 – 31^st^ Dec 2016 | VTE | 22,726 | Pre-matched:  123 d (60-183) ¥  Post-matched:  126 d (60-183) ¥ | Pre-matched: 134.7 d  Post-matched:  130.9 d | Newly |
|  |  | •WARF |  |  |  |  |  | Pre-matched: 167d (86-184) ¥  Post-matched:  156d¥ (79-184) | Pre-matched: 119.3 d  Post-matched:  120.5 d |  |
| Huang, 2020[30] | China | •RIV  •WARF | Semi-retrospective, semi-prospective Cohort study Single-centre | NR | Jan 2015 - Dec 2016 | PE | 128 | 6 | 3-6 | Not clear |
| Kohn, 2019[31] | USA | •RIV  •WARF | Retrospective Claims Database analysis Multicentre | USA Truven MarketScan | 1^st^ Jan 2012- 31^st^ Dec 2016 | VTE | 36,853 | Up to 12 | •RIV: 107 d ¥  •WARF: 113 d ¥ | Newly |
| Kreutz, 2019[32] | International | •RIV | Prospective Cohort study Multicentre | NR | 27^th^ Jun 2014- 31^st^ Oct 2015 | VTE | 1,987 | 215 d (149–323) ¥ | 184 d (I105–234) ¥ | Not clear |
|  |  | •Standard therapy (heparin, LMWH, fondaparinux, VKA) |  |  |  |  |  | 214 d (130–289) ¥ | 152 d (53–214) ¥ |  |
| Krivoshchekov, 2016[33] | Russia | •RIV  •VKA/ LMWH | Prospective Cohort study Single-centre | NR | 2014 - 2015 | VTE | 124 | 6 | ≥3 | Newly |
| Kucher, 2016[34] | Switzerland | •RIV  •LMWH /fonda-parinux/VKA | Retrospective Database analysis Multicentre | SWIss Venous ThromboEmbolism Registry (SWIVTER) | Nov 2012- Feb 2015 | VTE | 2,062 | 3 | NR | Previously |
| Kushnir, 2019[35] | USA | All | Retrospective Chart review Single-centre | Clinical Looking Glass | Mar 2013- Mar 2017 | VTE+AF | 366 | 196 d (89.4-457.3) Ɣ | 163.3 d | Newly |
|  |  | API |  |  |  |  |  | 163.3 d (90-233.3) Ɣ |  |  |
|  |  | RIV |  |  |  |  |  | 217.4 d (94.4-514.1) Ɣ | 217.4 d |  |
|  |  | WARF |  |  |  |  |  | 206.3 d (64.4-540.3) Ɣ | 191.5 d |  |
| Lai, 2016[36] | Singapore | •RIV | Retrospective Cohort study Single-centre | General Hospital of Singapore electronic databases | Sep 2009 -Oct 2014 | VTE | 231 | At least 3 | 11.1 (1.85) Ɣ | Previously + Newly |
|  |  | •WARF |  |  |  |  |  |  | 10.47 (0.79) Ɣ |  |
| Larsen, 2017[37] | Denmark | •RIV  •WARF | Prospective Matched-cohort Multicentre | •Danish Civil Registration system  •National Patient  •Danish National Prescription | 9^th^ Dec 2011- 28^th^ Feb 2016 | VTE | 5,004 | 3 and 6 | NR | Newly |
| Lopez-Nunez, 2019[38] | International | •API  •EDO  •DAB  •LMWH  •VKA | Retrospective Registry Multicentre | RIETE registry | Jan 2013- Apr 2018 | VTE | 10,054 | At least 3 | Initial therapy (Ɣ) in d:  •RIV: 21 (17)  •API: 10 (11)  •LMWH: 20 (16) | Not clear |
|  |  |  |  |  |  |  |  |  | Long-term therapy (Ɣ) in d:  •RIV: 144 (163)  •API: 162 (147)  •DAB: 173 (137)  •EDO: 125 (78)  •VKA: 251 (248) |  |
| Lutsey, 2018[39] | USA | •API  •RIV  •DAB  •WARF | Retrospective Database analysis Multicentre | USA Truven MarketScan | 2011 – 2015 | VTE | 83,831 | 13 Ɣ | NR | Newly |
| Lutsey, 2019[40] | USA | •API  •RIV  •WARF | Retrospective Cohort study Multicentre | USA Truven MarketScan | 1^st^ Jan 2011- 31^st^ Dec 2016 | VTE | 83,985 | 180 d | NR | Newly |
| Moustafa, 2018[41] | International | •API  •RIV  •LMWH  •Unfractionated heparin | Retrospective Registry Multicentre | RIETE registry | Jan 2013- Nov 2016 | VTE | 18,853 | NR | 176 (151) d Ɣ | Previously + Newly |
| Nagaoki, 2018[42] | Japan | •EDO  •WARF | Retrospective Cohort study Single-centre | NR | Dec 2011- Apr 2016 | PVT | 50 | Up to 6 | 6 | Newly |
| Naymagon, 2020[43] | USA | •WARF | Retrospective Cohort study Single-centre | NR | 1^st^ Jan 2000- 1^st^ Feb 2019 | VTE | 330 | 41.6 (44.3) Ɣ | 55.8 (27.4) Ɣ | Newly |
|  |  | •Enoxaparin |  |  |  |  |  |  | 33 (18.9) Ɣ |  |
|  |  | •API  •RIV  •DAB |  |  |  |  |  |  | 28.1 (11.3) Ɣ |  |
| Outler, 2018[44] | USA | •RIV  •WARF | Retrospective Chart review Single-centre | Grady Health System | Jan 2016- Jul 2017 | VTE | 314 | 3 | 3 | Previously |
| Ouyang, 2019[45] | USA | •RIV  •API  •Heparin  •LMWH | Retrospective Database analysis Single-centre | NR | Jan 2016 -Dec 2018 | VTE | 177 | NR | NR | Not clear |
| Patel, 2020[46] | USA | •RIV  •WARF | Retrospective Cohort study Single-centre | NR | Jan 2016 - Jun 2017 | VTE | 314 | 90 d | NR | Newly |
| Perales, 2020[47] | USA | •RIV  •VKA | Retrospective Chart review Multicentre | NR | Nov 1st, 2013 – 30th Sep 2017 | VTE+ AF | 176 | 12 | NR | Newly |
| Petrikov, 2017[48] | Russia | •DAB •WARF | Prospective Single-centre | NR | Jan 2011- Feb 2016 | DVT + PTE | 183 | 12 | •DAB: 1-12  •WARF: 3-12 | Previously |
| Poli, 2020[49] | Italy | •VKA | Prospective Cohort study Multicentre | START2 Register | Jul 2013 - Jul 2019 | VTE+AF | 344 | 362 d  1.6 (0.1-10.1) y ¥ | NR | Previously + Newly |
|  |  | •API  •RIV  •EDO  •DAB |  |  |  |  |  | 390 d  1.9 (0.1-7.2) y ¥ |  |  |
| Roetker, 2018[50] | USA | •API  •RIV  •WARF | Retrospective Claims analysis Multicentre | OptumLabs | 2^nd^ Nov 2012- 31^st^ Mar 2017 | VTE | 62,431 | 6 | NR | Not clear |
| Sebastian, 2018[51] | Switzerland | •RIV  •VKA | Prospective Cohort study Single-centre | Swiss Venous Stent registry | Since Jul 2011 | DVT | 111 | Max FU: 12  •Total: 24 (19) Ɣ  •RIV: 22 (16) Ɣ  •VKA: 31 (23) Ɣ | •RIV:180 (98)  d Ɣ  VKA: 284 (199) d Ɣ | Newly |
| Sena, 2020[52] | Turkey | •RIV  •WARF | Retrospective Registry Single-centre | Hospital  informatics database | Sep 2011- Apr 2018 | PE | 501 | 9 y ¥ | NR | Not clear |
| Sharifi, 2015 [53] | USA | •API  •RIV | Retrospective Cohort study Single-centre | NR | Jan 2012- Dec 2013 | PE | 159 | 18 (3) Ɣ | NR | Newly |
| Sindet-Pedersen, 2017[54] | Denmark | •RIV  •VKA | Retrospective Registry Multicentre | •Danish Civil Registration system  •National Patient  •Danish National Prescription | 6^th^ Feb 2012 – 30^th^ Sep 2016 | VTE | 12,318 | 6 | 3-6 | Newly |
| Søgaard, 2018[55] | Denmark | All | Retrospective  Registry Multicentre | •Danish Civil Registration system  •National Patient  •Danish National Prescription | 9^th^ Dec 2011- 31^st^ Mar 2017 | VTE | 19,957 | 2.6 (1.3-3.9) y ¥ | NR | Previously |
|  |  | RIV |  |  |  |  |  | 1.8 (1-2.9) y ¥ |  |  |
|  |  | VKA |  |  |  |  |  | 3.3 (1.9-4.5) y ¥ |  |  |
| Spyropoulos, 2019[56] | USA | •RIV  •VKA | Retrospective matched-cohort Multicentre | USA Truven MarketScan | 2011 - 2016 | VTE | 5,780 | • Overall: ≥3  • RIV: 10 Ɣ  • WAR: 10.5 Ɣ | •RIV: 181 d  •WARF: 193 d | Newly |
| Trutyak, 2016[57] | Russia | •RIV •WARF | Prospective | NR | NR | Acute idiopathic DVT | 71 | 12 | NR | Not clear |
| Utne, 2018[58] | Norway | RIV | Retrospective Cross-sectional Multicentre | Thrombosis Registry of Østfold Hospital – The TROLL registry | 2011 and 2014 | DVT | 309 | 22 (24–26) ¥ | 6 (3–12) ¥ | Previously |
|  |  | WARF |  |  |  |  |  | 27 (24–31) ¥ | 6 (3–19) ¥ |  |
| Wang, 2017[59] | USA | •RIV  •WARF /LMWH | Retrospective matched cohort Multicentre | Veterans’ Health Administration (VHA)’s national Medical Statistical Analysis System Dataset and Decision Support System | 1^st^ Oct 2010- 30^th^ Sep 2015 | PE | 6,746 | 3 | NR | Newly |
| Weeda, 2016[60] | USA | •RIV  •WARF | Retrospective Claims analysis Multicentre | USA Premier Hospital claims | Nov 2012- Mar 2015 | PE | 624 | NR | NR | Previously |
| Weycker, 2018[61] | USA | •API  •WARF | Retrospective Cohort study Multicentre | 2 large US private healthcare claims repositories | Mar 2014- Jun 2017 | VTE | 25,951 | Max. FU: 180 d  •API: 137 d Ɣ  •WAR: 172 d Ɣ | •API:137 d  •WARF :142 d | Previously + Newly |
| Weycker, 2018a[62] | USA | API | Retrospective Claims analysis Multicentre | 4 US databases  •Truven MarketScan + Medicare  • PharMetrics  • Humana | 1^st^ Mar 2014 – 30^th^ Jun 2017 | VTE | 35,756 | Max. FU: 180 d  143 (57) d Ɣ | 113 (60) d Ɣ  Matched cohorts:  116.2 (61) d Ɣ | Previously |
|  |  | WARF |  |  |  |  |  | 152 (52) d Ɣ | 126.2 (60.2) d Ɣ  Matched cohorts:  135.8 (71) d Ɣ |  |
| Wysokinski, 2018[63] | USA | •API  •RIV  •LMWH  •VKA | Prospective Registry Single-centre | Mayo Thrombophilia Clinic Anticoagulants Registry | 1^st^ Mar 2013- Jan 30 2018 | VTE | 896 | NR | ≥3 | Not clear |
| Yuko, 2018[64] | Japan | •EDO  •WARF | Retrospective Cohort study | NR | NR | PVT | 65 | 6 | WARF: 2 w | Previously |
| Zakai, 2019[65] | USA | All | Retrospective Database analysis Multicentre | USA Truven MarketScan | 2011 -2017 | VTE | 26,126 | 1.57 y Ɣ | NR | Not clear |
|  |  | API |  |  |  |  |  | 0.96 y Ɣ |  |  |
|  |  | RIV |  |  |  |  |  | 1.52 y Ɣ |  |  |
|  |  | WARF |  |  |  |  |  | 1.75 y Ɣ |  |  |

Abbreviations: AF – Arterial Fibrillation, API – Apixaban, d – days, DVT - Deep Vein Thrombosis, DAB – dabigatran, EDO – edoxaban, FU – follow-up, PE - Pulmonary Embolism, LMWH – Low Molecular Weight Heparin, NOAC – Non-Vitamin K Antagonist Oral Anticoagulants, NR – Not Reported, PHSs – primary hypercoagulable state, PVT – portal vein thrombosis, RIV – rivaroxaban, USA – United States of America, VKA – Vitamin K Antagonist, VTE - Venous Thromboembolism, WARF - Warfarin, w – weeks, y – years.

¥ – median or median (interquartile), Ɣ – mean or mean (standard deviation).

Supplementary table 2 Patient baseline characteristics from SLR-identified studies

| **Au thor, year**  **(Acronym)** | **Treatment arm** | **Female (%)** | **Age** | | **Type of VTE** **n (%)** | **VTE aetiology** **n (%)** | **BMI (kg/m^2^)** | | **Weight (Kg)**  **mean (SD)** | **CrCI level mean (SD) (mL/min)** | **Active cancer n (%)** |
| --- | --- | --- | --- | --- | --- | --- | --- | --- | --- | --- | --- |
|  |  |  | **mean (SD)** | **median (IQR)** |  |  | **mean (SD)** | **median (IQR)** |  |  |  |
| Ageno, 2016[1] | RIV | 1,191 (45) | NR | 59 (45-71) | •DVT: 2399 (92)  •DVT + PE: 220 (8) | •Provoked: 896 (34)  •Unprovoked: 1692 (65)  •Missing: 31 (1) | 28 (5.2) | NR | NR | NR | 146 (6) |
|  | Heparin or fondaparinux + VKA | 1,033 (48) | NR | 66 (47-73) | •DVT: 1894 (88)  •DVT+PE: 255 (12) | •Provoked: 823 (38)  •Unprovoked: 1300 (61)  •Missing: 26 (1) | 28.4 (6.9) | NR | NR | NR | 411 (19) |
| Badreldin, 2018[2] | WARF | 86 (47.8) | 61.6 (15.1) | NR | •DVT: 102 (55.6)  •PE: 50 (27.8)  •Both: 28 (15.6) | NR | 30.3 (8.7) | NR | NR | 81.0 (34.4) | NR |
|  | NOAC (API, RIV, EDO, DAB) | 105 (40.2) | 62.2 (17.6) | NR | •DVT: 159 (60.9) •PE :63 (24.1)  •Both: 39 (14.9) | NR | 29.3 (7.1) | NR | NR | 85.6 (23.3) | NR |
| Bouget, 2020[3] | VKA | 3131 (55.3) | 66.9 | NR | NR | NR | NR | NR | NR | NR | NR |
|  | NOAC (API, DAB, RIV) | 2,213 (46.9) | 58.9 | NR | NR | NR | NR | NR | NR | NR | NR |
| Bounameaux, 2020[4] | VKA | 1,552 (48.6) | NR | 59 (45-70) | • DVT: 1979 (61.9)  •PE: 800 (25)  •PE + DVT: 417 (13) | NR | NR | 27.8  (24.3-32.1) | NR | NR | 122 (3.8) |
|  | NOAC (API, RIV, EDO, DAB) | 2,323 (48.5) | NR | 61 (47-72) | • DVT: 2894 (60.4)  •PE: 1081 (22.6)  •PE and DVT: 816 (17) | NR | NR | 27.5  (24.3-31.6) | NR | NR | 201 (4.2) |
| Bryk, 2016[5] | RIV | 76 (100) | 36.2 (9.1) | NR | •DVT: 31 (41)  •PE: 18 (24)  •Concomitant DVT and PE: 23 (30)  •Others: 4 (5) | •Surgery/trauma: 9 (12)  •Pregnancy: 11 (15)  •Combined hormonal contraceptives/Hormone replacement therapy: 30 (39)  •Immobilization: 2 (3)  •Positive family history: 22 (29)  •Unprovoked VTE: 24 (32)  •Inherited thrombophilia: 28 (37)  •Acquired thrombophilia: 15 (20)  •Smoking: 14 (18)  •Obesity: 19 (25) | NR | 24.3  (21.8–29.7) | NR | NR | NR |
|  | VKA | 45 (100) | 38.2 (10.5) | NR | •DVT: 11 (24)  •PE: 18 (40)  •Concomitant DVT and PE: 11 (24)  • Others: 5 (12) | •Surgery/trauma: 7 (16)  •Pregnancy: 3 (7)  •Combined hormonal contraceptives/Hormone replacement therapy: 18 (40)  •Immobilization: 0 (0)  •Positive family history: 17 (38)  •Unprovoked: 17 (38)  •Inherited thrombophilia: 12 (27)  •Acquired thrombophilia: 5 (11)  •Smoking: 7 (16)  •Obesity: 9 (20) | NR | 25.4 (21.9–29.1) | NR | NR | NR |
| Bui, 2019[6] | RIV | 53 (63.9) | 57.08 (19.5) | NR | •DVT: 71 (85.5)  •DVT/PE: 3 (3.6)  •PE: 9 (10.8) | Unprovoked: 62 (74.7) | NR | NR | NR | NR | 5 (6) |
|  | Enoxaparin + VKA | 77 (74.8) | 60.2 (16.4) | NR | •DVT: 91 (87.5)  •DVT/PE: 5 (4.8)  •PE: 7 (6.7) | Unprovoked: 71 (68.3) | NR | NR | NR | NR | 9 (8.4) |
| Carroll, 2018[7] | RIV | 1,696 (51.4) | 62.6 | NR | •PE: 1,152 (34.9)  •DVT: 2039 (61.8)  •PE+DVT: 106 (3.2) | NR | NR | NR | NR | NR | NR |
|  | API |  |  |  |  |  |  |  |  |  |  |
|  | WARF |  |  |  |  |  |  |  |  |  |  |
| Chaudhari, 2019[8] | RIV | NR | NR | NR | NR | NR | NR | NR | NR | NR | NR |
|  | Enoxaparin + VKA | NR | NR | NR | NR | NR | NR | NR | NR | NR | NR |
| Chu, 2017[9] | RIV | 16 (41) | 53 (17) | NR | • DVT: 39 (95)  • PE: 2 (5)  • DVT and PE:0 | NR | NR | NR | NR | NR | NR |
|  | RIV + Enoxaparin | 18 (53) | 60 (16) | NR | • DVT: 30 (88)  • PE: 3 (9)  •DVT & PE: 1 (3) | NR | NR | NR | NR | NR | NR |
| Coleman, 2017[10] | RIV | 6641 (48.8) | NR | NR | PE: 6,192 (45.5) | NR | NR | NR | NR | NR | NR |
|  | WARF | 15864 (49.2) | NR | NR | PE: 14,381 (44.6) | NR | NR | NR | NR | NR | NR |
| Coleman, 2017a [11] | RIV | 5,127 (49) | ≥60: 4,687 (44.8) | NR | PE: 4,635 (44.3) | NR | NR | NR | NR | NR | NR |
|  | WARF | 13,008 (49.1) | ≥60: 9,140 (34.5) | NR | PE: 11,683 (44.1) | NR | NR | NR | NR | NR | NR |
| Coleman, 2018b[12] | RIV | 895 (65.6) | 81.8 (6.4) | NR | PE: 569 (41.7) | NR | NR | NR | NR | NR | NR |
|  | WARF | 3,545 (64.4) | 82.4 (6.3) | NR | PE: 2,499 (45.4) | NR | NR | NR | NR | NR | NR |
| Coleman, 2018[13] | RIV | 2,298 (48.6) | NR | 57 (47-65) | PE: 2,000 (44.9) | NR | NR | NR | NR | NR | 1,171 (26.3) |
|  | WARF | 6,727 (51.1) | NR | 59 (48-69) | PE: 5,700 (43.3) | NR | NR | NR | NR | NR | 3,752 (28.5) |
| Coleman, 2018c[14] | RIV | 202 (50.1) | 50.3 (14.5) | NR | PE: 217 (53.9) | NR | NR | NR | NR | NR | NR |
|  | WARF | 199 (49.4) |  |  | PE: 209 (51.9) |  |  |  |  |  |  |
| Coleman, 2018d[15] | RIV | 1,952 (51.3) | NR | 58 (48-66) | PE: 1,774 (46.6) | Provoked: 3806 (100) | NR | NR | NR | NR | 556 (14.6) |
|  | Discontinued AC | 604 (49) | NR | 58 (48-67) | PE: 554 (46.8) | Provoked: 1184 (100) | NR | NR | NR | NR | 185(15.6) |
| Coleman, 2018a [16] | RIV | NR | NR | NR | NR | Unprovoked: 10489 (100) | NR | NR | NR | NR | NR |
|  | WARF | NR | NR | NR | NR | Unprovoked: 26364 (100) | NR | NR | NR | NR | NR |
| Costa , 2020[17] | RIV | 906 (43.81) | NR | 50 (39-62) | PE±DVT: 374 (18.09) | NR | NR | NR | NR | NR | NR |
|  | WARF | 917 (44.34) | NR | 51 (40-64) | PE±DVT: 370 (17.89) | NR | NR | NR | NR | NR | NR |
| Costa , 2020a[18] | RIV | 3627 (53.7) | NR | NR | PE ± DVT: 1,351 (20.7) | NR | NR | NR | NR | NR | NR |
|  | WARF | 3695 (54.7) | NR | NR | PE ± DVT: 1,648 (24.4) | NR | NR | NR | NR | NR | NR |
| Dawwas, 2018[19] | API | 1411 (51) | 63 | NR | VTE or PE: 2,768 (100) | NR | NR | NR | NR | NR | NR |
|  | WARF | 1467 (53) | 63 | NR | VTE or PE: 2,768 (100) | NR | NR | NR | NR | NR | NR |
| Dawwas, 2020[20] | API | 4,183 (51.7) | 58.9 (16.3) | NR | NR | •Provoked:4,188 (51.7)  •Unprovoked: 3,848 (47.5) | NR | NR | NR | NR | 1,168 (14.4) |
|  | WARF | 14,999 (52.1) | 59.3 (15.8) | NR | NR | •Provoked:15,282 (53)  •Unprovoked: 11,969 (41.5) | NR | NR | NR | NR | 4,648 (16.1) |
| De Crema, 2015[21] | RIV | 52 (100) | NR | 38 (23-43) | • DVT: 33 (63)  • PE: 9 (17) | NR | NR | 24 (21-30) | NR | NR | 2 (3.8) |
|  | VKA | 52 (100) | NR | 29 (24-49) | • DVT: 18 (35)  • PE: 14 (27) | NR | NR | 25 (21-28) | NR | NR | 0 (0) |
| Desai, 2016[22] | RIV | 43 (59.7) | 63.3 (16.7) | NR | •PE: 21 (29.2)  •DVT: 23 (31.9) | •Hormonal: 5 (6.9)  •Hypercoagulable state: 3 (4.2)  •Immobilization: 4 (5.6)  •Surgery: 8 (11.1)  •Trauma: 3 (4.2)  •Travel: 8 (11.1)  •Unprovoked: 32 (44.4) | NR | NR | 87.4 (25.6) | NR | 16 (22.2) |
|  | WARF | 106 (52.2) | 68.7 (15.5) | NR | •PE: 59 (29.1)  •DVT: 82 (40.4) | •Hormonal: 7 (3.5)  •Hypercoagulable state: 16 (8.0)  •Immobilization: 21 (10.5)  •Surgery: 15 (7.5)  •Trauma: 8 (4.0)  •Travel: 11 (5.5)  •Unprovoked: 92 (45.8) | NR | NR | 85.7 (22.8) | NR | 43 (21.2) |
|  | Enoxaparin + WARF | 49 (55.1) | 60.8 (15.6) | NR | •PE: 22 (24.7)  •DVT: 46 (51.7) | •Hormonal: 5 (5.6)  •Hypercoagulable state: 8 (9.0)  •Immobilization: 8 (9.0)  •Surgery: 10 (11.2)  •Trauma: 2 (2.3)  •Travel: 7 (7.9)  •Unprovoked: 41 (46.1) | NR | NR | 86.7(21.8) | NR | 10 (11.2) |
|  | Enoxaparin | 29 (58) | 59.2 (18.2) | NR | •PE: 14 (28.0)  •DVT: 22 (44.0) | •Hormonal: 2 (4.0)  •Hypercoagulable state: 2 (4.0)  •Immobilization: 2 (4.0)  •Surgery: 3 (6.0)  •Trauma: 3 (6.0)  •Travel: 2 (4.0)  •Unprovoked: 6 (12.0) | NR | NR | 82.1 (25.1) | NR | 22 (44.0) |
| Ferreira, 2020[23] | RIV | 39 (54.9) | NR | 42 (33–56) | NR | • Idiopathic: 34 (47.9)  • Provoked: 37 (52.1) | NR | 27.3 (23.8–31.5) | NR | NR | NR |
|  | Enoxaparin/WARF | 49 (84.5) | NR | 44 (29.5–52) | NR | • Idiopathic: 22 (37.1)  • Provoked: 36 (62.1) | NR | 29.3 (25.6–34.5) | NR | NR | NR |
| Fung, 2019[24] | RIV | 53 (58.9) | 63.3 (18.2) | NR | •DVT: 65 (72.2)  •PE: 25 (27.8) | •Unprovoked: 62 (68.9)  •Provoked: 28 (31.1) | NR | NR | NR | NR | NR |
|  | WARF | 48 (52.7) | 61.8 (17.9) | NR | •DVT: 70 (76.9)  •PE: 21 (23.1) | •Unprovoked: 73 (80.2)  •Provoked: 18 (19.8) | NR | NR | NR | NR | NR |
| Gaertner, 2017[25] | RIV | 157 (51) | 62.2 (18.2) | NR | •DVT: 32 (10.4)  •PE: 74 (24)  •PE + DVT :202 (65.6) | •Unprovoked: 257 (83.4)  •Provoked: 51 (16.6) | NR | NR | NR | NR | 8 (2.6) |
|  | VKA | 59 (53.2) | 70.7 (17.6) | NR | •DVT: 9 (8.1)  •PE:33 (29.7)  •PE + DVT :69 (62.2) | •Unprovoked: 90 (81.1)  •Provoked: 21 (18.9) | NR | NR | NR | NR | 9 (8.1) |
|  | Heparin/ fondaparinux | 80 (15.9) | 68.6 (17.5) | NR | •DVT: 20 (25)  •PE: 19 (23.8)  •PE + DVT :41 (51.2) | •Unprovoked: 37 (46.2)  •Provoked: 43 (53.8) | NR | NR | NR | NR | 58 (72.5) |
| Goldhaber, 2020[26] | VKA | NR | NR | NR | NR | NR | NR | NR | NR | NR | NR |
|  | DAB | NR | NR | NR | NR | NR | NR | NR | NR | NR | NR |
| Gollamudi, 2018[27] | API | NR | NR | NR | NR | NR | NR | NR | NR | NR | NR |
|  | WARF | NR | NR | NR | NR | NR | NR | NR | NR | NR | NR |
| Guo, 2020[28] | API | 3,951 (44.6) | 52.6 (12.9) | NR | •DVT:4,947 (55.8)  •PE +/- DVT: 3,911 (44.2)  •PE with DVT 1,457 (16.4)  •PE without DVT 2,454 (27.7) | •Provoked: 2,709 (30.6)  •Unprovoked: 6,149 (69.4) | NR | NR | NR | NR | 0 (0) |
|  | WARF | 3,948 (44.6) | 52.5 (12.9) | NR | •DVT:4,879 (55.1)  •PE +/- DVT 3,979 (44.9)  •PE with DVT 1,338 (15.1)  •PE without DVT 2,641 (29.8) | •Provoked:2,741 (30.9)  •Unprovoked: 6,117 (69.1) | NR | NR | NR | NR | 0 (0) |
| Hlavacek, 2019[29] | WARF | 7167 (63.1) | 77.6 | 8.1 | •DVT: 6162 (54.2)  •PE+DVT: 1783 (15.7)  •PE without DVT: 3418 (30.1) | •Provoked:3651 (32.1)  •Unprovoked: 7712 (67.9) | NR | NR | NR | NR | NR |
|  | API | 7,101 (62.5) | 77.6 | 8.1 | •DVT:6138 (54)  •PE+DVT: 1772 (15.6)  •PE without DVT: 3453 (30.4) | •Provoked: 3680 (32.4)  •Unprovoked:7683 (67.6) | NR | NR | NR | NR | NR |
| Huang, 2020[30] | RIV | 45 (52.33) | 58.08 (14.52) | NR | •PE without DVT: 48 (55.81)  •PE with DVT: 38 (44.19) | NR | 24.28 (3.61) | NR | 64.84 (12.09) | NR | 18 (18.6) |
|  | WARF | 22 (52.38) | 52.55 (14.08) | NR | •PE without DVT: 21 (50)  •PE with DVT: 21 (50) | NR | 23.22 (3.37) | NR | 61.76 (12.06) | NR | NR |
| Kohn, 2019[31] | RIV | 4,835 (46.1) | 56 (NR) | NR | PE ± DVT 4898 (46.7) | NR | NR | NR | NR | NR | NR |
|  | WARF | 12,760 (48.4) | 56 (NR) | NR | PE ± DVT 12 655 (48) | NR | NR | NR | NR | NR | NR |
| Kreutz, 2019[32] | RIV | 662 (51.5) | 59.6 (17.1) | NR | •DVT: 882 (68.6)  •PE+DVT: 238 (18.5)  •PE only: 165 (12.8) | •Provoked: 480 (37.4)  •Unprovoked: 805 (62.6) | NR | NR | 71.6 (16.9) | 89.8 (42.4) | 216 (16.8) |
|  | LMWH/ fondaparinux +VKA | 222 (55.2) | 58 (18) | NR | •DVT: 238 (59.2)  •PE+DVT: 80 (19.9)  •PE only: 84 (20.9) | •Provoked: 192 (47.8)  •Unprovoked: 210 (52.2) | NR | NR | 73.5 (17.6) | 89.8 (61.7) | 69 (17.2) |
| Krivoshchekov, 2016[33] | RIV | NR | NR | NR | NR | •Genetic mutations, resulting in thrombophilia: 59 (47.6)  •Unprovoked: 50 (40.4) | NR | NR | NR | NR | 15 (12) |
|  | LMWH / VKA | NR | NR | NR | NR | NR | NR | NR | NR | NR |  |
| Kucher, 2016[34] | RIV | 183 (43.9) | 56 (17) | NR | NR | NR | NR | NR | NR | NR | 40 (9.6) |
|  | •LMWH  •Heparin /fondaparinux  • VKA | 188 (45.1) | 57 (18) | NR | NR | NR | NR | NR | NR | NR | 46 (11) |
| Kushnir, 2019[35] | API | 35 (75) | 53.3 (13.9) | NR | NR | NR | NR | 43.3  (41.2–49.4) | NR | NR | NR |
|  | RIV | 100 (66) | 52.4 (14.7) | NR | NR | NR | NR | 43.7 (41.1–48.8) | NR | NR | NR |
|  | WARF | 118 (71) | 52.6 (14.5) | NR | NR | NR | NR | 45.3 (41.4–52.5) | NR | NR | NR |
| Lai, 2016[36] | WARF | 78 (56.9) | 62.33 (1.44) | NR | NR | NR | NR | NR | NR | NR | NR |
|  | RIV | 45 (47.9) | 63.32 (1.6) | NR | NR | NR | NR | NR | NR | NR | NR |
| Larsen, 2017[37] | RIV | 793 (45) | 62.6 (17.4) | NR | NR | Unprovoked: 1751 (100) | NR | NR | NR | NR | NR |
|  | WARF | 1473 (45) | 62.6 (17) | NR | NR | Unprovoked: 3253 (100) | NR | NR | NR | NR | NR |
| Lopez-Nunez, 2019[38] | NOAC (Initial Therapy) | 297 (63) | 78 (11) | NR | PE: 242 (51) | NR | NR | NR | 72 (14) | 60 (21) | 66 (14) |
|  | LMWH (Initial Therapy) | 5,286 (62) | 80 (10) | NR | PE: 4932 (58) | NR | NR | NR | 71 (14) | 55 (24) | 2,168 (25) |
|  | NOAC (Long Term Therapy) | 817 (63) | 79 (9.9) | NR | PE: 795 (61) | NR | NR | NR | 72 (15) | 59 (21) | 157 (12) |
|  | VKA (Long Term Therapy) | 3,121 (62) | 80 (9.6) | NR | PE: 3156 (63) | NR | NR | NR | 72 (14) | 54 (23) | 626 (12) |
| Lutsey, 2018[39] | API | NR | NR | NR | NR | NR | NR | NR | NR | NR | NR |
|  | DAB | NR | NR | NR | NR | NR | NR | NR | NR | NR | NR |
|  | RIV | NR | NR | NR | NR | NR | NR | NR | NR | NR | NR |
|  | WARF | NR | NR | NR | NR | NR | NR | NR | NR | NR | NR |
| Lutsey, 2019[40] | WARF | 23155 (50.1) | 58.4 (15.9) | NR | NR | NR | NR | NR | NR | NR | NR |
|  | API | 3413 (50.3) | 60.4 (16.2) | NR | NR | NR | NR | NR | NR | NR | NR |
|  | RIV | 15,243 (49.2) | 56.4 (15.4) | NR | NR | NR | NR | NR | NR | NR | NR |
| Moustafa, 2018[41] | LMWH | NR | 66 (17) | NR | NR | NR | NR | NR | NR | NR | NR |
|  | RIV | NR | NR | NR | NR | NR | NR | NR | NR | NR | NR |
|  | API | NR | NR | NR | NR | NR | NR | NR | NR | NR | NR |
|  | DAB | NR | NR | NR | NR | NR | NR | NR | NR | NR | NR |
| Nagaoki, 2018[42] | EDO | 7 (35) | NR | 69 (53-74) | NR | NR | NR | NR | NR | NR | NR |
|  | WARF | 13(39) | NR | 67 (24-83) | NR | NR | NR | NR | NR | NR | NR |
| Naymagon, 2020[43] | WARF | 51 (47.2) | 50.4 (14.8) | NR | • Main PV only:28 (25.9)  •Left or right PV only: 23 (21.3)  •Main PV + additional SVT: 57 (52.8)  •Occlusive: 68 (63)  •Nonocclusive: 40 (37) | NR | NR | NR | NR | NR | NR |
|  | Enoxaparin | 43 (61.4) | 51.4 (16.9) | NR | •Main PV only 15 (21.4)  •Left or right PV only19 (27.1)  •Main PV + additional SVT36 (51.4)  •Occlusive: 36 (51.4)  •Nonocclusive: 34 (48.6) | NR | NR | NR | NR | NR | NR |
|  | NOAC | 46 (49.5) | 47.1 (15.2) | NR | • Main PV only: 22 (23.7)  •Left or right PV only: 23 (24.7)  •Main PV + additional SVT: 48 (51.6)  •Occlusive: 53 (57)  •Nonocclusive: 40 (43) | NR | NR | NR | NR | NR | NR |
| Outler, 2018[44] | RIV | NR | NR | NR | NR | NR | NR | NR | NR | NR | NR |
|  | WARF | NR | NR | NR | NR | NR | NR | NR | NR | NR | NR |
| Ouyang, 2019[45] | RIV | NR | NR | NR | NR | NR | NR | NR | NR | NR | NR |
|  | API | NR | NR | NR | NR | NR | NR | NR | NR | NR | NR |
|  | Unfractionated heparin | NR | NR | NR | NR | NR | NR | NR | NR | NR | NR |
|  | LMWH | NR | NR | NR | NR | NR | NR | NR | NR | NR | NR |
| Patel, 2020[46] | RIV | 88 (37) | 51.4 (14.7) | NR | • DVT: 132 (56)  • PE: 100 (42) | NR | NR | NR | NR | NR | NR |
|  | WARF | 31 (40) | 54.3 (13.2) | NR | • DVT: 47 (60)  • PE: 24 (31) | NR | NR | NR | NR | NR | NR |
| Perales, 2020[47] | RIV | 40 (48) | 56 (14) | NR | • DVT: 28 (33)  • PE: 26 (31) | NR | NR | 45 (41-51) | 133  (121-150) ¥ | 0.9 (0.8-1) ¥ | 3 (2) |
|  | WARF | 41 (45) | 55 (15) | NR | • DVT: 30 (33)  • PE: 38 (41) | NR | NR | 44 (41-50) | 134 (118-154) ¥ | 0.9 (0.8-1.3) ¥ | 7 (4) |
| Petrikov, 2017[48] | DAB | 85 (46.4) | 53.6 (4.2) | NR | NR | NR | NR | NR | NR | NR | NR |
|  | WARF |  | 49.6 (4.1) | NR | NR | NR | NR | NR | NR | NR | NR |
| Poli, 2020[49] | NOAC | 65 (39.2) | NR | 76.7(10-95) | VTE: 45 (27.1) | NR | NR | NR | NR | NR | 6 (3.7) |
|  | VKA | 68 (38.2) | NR | 76.6(70-82) | VTE: 53 (30.1) | NR | NR | NR | NR | NR | 9 (5) |
| Roetker, 2018[50] | WARF | 18,459 (51.7) | 64 (16) | NR | PE: 15,174 (42.5) | NR | NR | NR | NR | NR | 6,605 (18.5) |
|  | RIV | 10,385 (49.3) | 59 (16) | NR | PE: 8,699 (41.3) | NR | NR | NR | NR | NR | 3,454 (16.4) |
|  | WARF | 8,525 (52.4) | 65 (16) | NR | PE: 7,093 (43.6) | NR | NR | NR | NR | NR | 2,896 (17.8) |
|  | API | 2,962 (52.3) | 64 (16) | NR | PE: 2,701 (47.7) | NR | NR | NR | NR | NR | 980 (17.3) |
| Sebastian, 2018[51] | RIV | 42 (58) | 49 (21) | NR | NR | Provoked: 50 (68) | 26.6 (5.9) | NR | NR | NR | 3 (4) |
|  | VKA | 28 (74) | 40 (18) | NR | NR | Provoked: 28 (74) | 25 (3.9) | NR | NR | NR | 1 (3) |
| Sena, 2020[52] | RIV | 254 (50.7) | 53.54 (15.02) | NR | NR | NR | 28.15 (5.78) | NR | NR | NR | NR |
|  | WARF |  |  |  |  |  |  |  |  |  |  |
| Sharifi, 2015 [53] | NOAC | 77 (48) | 67 (7) | NR | •Bilateral PE: 132 (83)  •Concomitant: DVT 2 (58) | NR | 31 (6) | NR | NR | NR | 32 (14) |
| Sindet-Pedersen, 2017[54] | RIV | 2490 (46) | NR | 66 (52-76) | •PE: 2,214 (40.9)  •DVT: 3,197 (59.1) | NR | NR | NR | NR | NR | NR |
|  | VKA | 3161 (45.8) | NR | 66 (52-77) | •PE: 2,605 (37.7)  •DVT: 4,302 (62.3) | NR | NR | NR | NR | NR | NR |
| Søgaard, 2018[55] | WARF | 5,504 (48.3) | 64.2 (17.2) | NR | •DVT: 6,231 (54.7)  •PE: 5,159 (45.3) | Provoked: 2,747 (24.1) | NR | NR | NR | NR | NR |
|  | RIV | 4,000 (46.7) | 64.4 (17) | NR | •DVT: 4,654 (54.3)  •PE: 3,913 (45.7) | Provoked: 1,670 (19.5) | NR | NR | NR | NR | NR |
| Spyropoulos, 2019[56] | RIV | 1749 (60.5) | 53.3 (12.9) | NR | NR | NR | NR | NR | NR | NR | NR |
|  | WARF | 1740 (60.2) | 53.1 (13.1) | NR | NR | NR | NR | NR | NR | NR | NR |
| Trutyak, 2016[57] | RIV | 28 (39.4) | NR | NR | NR | NR | NR | NR | NR | NR | NR |
|  | WARF |  | NR | NR | NR | NR | NR | NR | NR | NR | NR |
| Utne, 2018[58] | RIV | 52 (32.3) | 60 (14) | NR | • Proximal DVT:101 (63)  • Recurrent ipsilateral DVT:4 (3)  • Recurrent contralateral DVT: 2 (1) | • Provoked DVT: 74 (46)  • Orthopedic surgery: 31 (19)  • Other extensive surgery: 6 (4)  •Trauma/hospitalization: 22 (14)  • Long-haul flight: 15 (9)  • Childbirth: 0 (0) | NR | NR | NR | NR | NR |
|  | WARF | 64 (43.24) | 63 (14) | NR | • Proximal DVT:92 (62)  • Recurrent ipsilateral DVT:7(5)  • Recurrent contralateral DVT:5 (3) | • Provoked DVT: 72 (49)  • Orthopedic surgery: 23 (16)  • Other extensive surgery: 6 (4)  • Trauma/hospitalization: 23 (16)  • Long-haul flight: 15 (10)  • Childbirth: 5 (3) | NR | NR | NR | NR | NR |
| Wang, 2017[59] | RIV | 12 (5.8) | 65 (13.9) | 66 | Hospitalized DVT: 58 (27.9) | NR | 32.2 (13.5) | NR | NR | 1.9 (2) | 76 (36.5) |
|  | LMWH, unfractionated heparin, and WARF | 239 (5.1) | 66.9 (15.2) | 66 | Hospitalized DVT: 1433 (30.9) | NR | 30.5 (8.7) | NR | NR | 2.2 (2.4) | 1600 (34.5) |
| Weeda, 2016[60] | RIV | 86 (55.1) | NR | NR | NR | NR | NR | NR | NR | NR | 8 (2.6) |
|  | WARF | 173 (55.4) | NR | NR | NR | NR | NR | NR | NR | NR | 5 (1.6) |
| Weycker, 2018[61] | API | NR | NR | NR | NR | NR | NR | NR | NR | NR | NR |
|  | WARF | NR | NR | NR | NR | NR | NR | NR | NR | NR | NR |
| Weycker, 2018a[62] | API | 8,595 (48.1) | 60 (16) | NR | •PE +/- DVT: 7,322 (41)  •PE with DVT: 1,655 (22.6)  •PE without DVT: 5,667 (77.4)  •DVT only: 10,556 (59) | •Provoked: 4,069 (22.8)  •Unprovoked: 13,809 (77.2) | NR | NR | NR | NR | NR |
|  | WARF | 8,599 (48.1) | 60 (16) | NR | •PE +/-DVT: 7,322 (41)  •PE with DVT:1,635 (22.3)  •PE without DVT:5,687 (77.7)  •DVT only: 10,556 (59) | •Provoked:4,069 (22.8)  •Unprovoked: 13,809 (77.2) | NR | NR | NR | NR | NR |
| Wysokinski, 2018[63] | API | 110 (39.1) | 63 (14.1) | NR | NR | NR | NR | NR | NR | NR | 135 (48) |
|  | RIV | 141 (48.1) | 58.3 (14.2) | NR | NR | NR | NR | NR | NR | NR | 120 (41) |
|  | LMWH | 101 (40.2) | 62.5 (12.9) | NR | NR | NR | NR | NR | NR | NR | 214 (85.3) |
|  | VKA | 28 (39.4) | 62.5 (14.5) | NR | NR | NR | NR | NR | NR | NR | 18 (25.4) |
| Yuko, 2018[64] | WARF | NR | NR | NR | PVT: 30 (100) | NR | NR | NR | NR | NR | NR |
|  | EDO | NR | NR | NR | PVT: 35 (100) | NR | NR | NR | NR | NR | NR |
| Zakai, 2019[65] | RIV | NR | NR | NR | NR | NR | NR | NR | NR | NR | NR |
|  | API | NR | NR | NR | NR | NR | NR | NR | NR | NR | NR |
|  | WARF | NR | NR | NR | NR | NR | NR | NR | NR | NR | NR |

AC – Anticoagulation; API – Apixaban; BMI – Body mass index; CrCl – Creatinine Clearance; NOAC – Direct Oral Anticoagulant; DAB – Dabigatran; DVT – Deep Vein Thrombosis; EDO – Edoxaban; IQR – interquartile; RIV – Rivaroxaban; LMWH – Low Molecular Weight Heparin; NOAC – Non-Vitamin K Antagonist Oral Anticoagulants; NR – Not Reported; PE – Pulmonary Embolism; PVT – Portal vein thrombosis; SD – Standard Deviation; VKA – Vitamin K Antagonist; VTE – Venous Thromboembolism; WARF – Warfarin; ¥ – median (interquartile).

Supplementary table 3 Modified Downs and Black questionnaire

| **Question no.** | **Score** |
| --- | --- |
| **REPORTING** | **Yes/No/Partially** |
| Q1. Is the objective of the study clear? | Yes = 1, No = 0 |
| Q2. Are the main outcomes clearly described in the Introduction or Methods? | Yes = 1, No = 0 |
| Q3. Are characteristics of the patients included in the study clearly described? | Yes = 1, No = 0 |
| Q4. Are the interventions clearly described? | Yes = 1, No = 0 |
| Q5. Are the distributions of principal confounders in each group of subjects clearly described? | Yes = 2, Partially = 1, No = 0 |
| Q6. Are the main findings of the study clearly described? | Yes = 1, No = 0 |
| Q7. Does the study estimate random variability in data for main outcomes? | Yes = 1, No = 0 |
| Q8. Have all the important adverse events consequential to the intervention been reported? | Yes = 1, No = 0 |
| Q9. Have characteristics of patients lost to follow-up been described? | Yes = 1, No = 0 |
| Q10. Have actual probability values been reported for the main outcomes except probability < 0.001? | Yes = 1, No = 0 |
| **EXTERNAL VALIDITY** | **Yes/No/Unclear** |
| Q11. Were subjects who were asked to participate in the study representative of the entire population recruited? | Yes = 1, No = 0, Unclear = 0 |
| Q12. Were those subjects who were prepared to participate representative of the recruited population? | Yes = 1, No = 0, Unclear = 0 |
| Q13. Were staff, places, and facilities where patients were treated representative of treatment most received? | Yes = 1, No = 0, Unclear = 0 |
| **INTERNAL VALIDITY** | **Yes/No/Unclear** |
| Q14. Was an attempt made to blind study subjects to the intervention? | Yes = 1, No = 0, Unclear = 0 |
| Q15. Was an attempt made to blind those measuring the main outcomes? | Yes = 1, No = 0, Unclear = 0 |
| Q16. If any of the results of the study were based on data dredging was this made clear? | Yes = 1, No = 0, Unclear = 0 |
| Q17. Was the time period between intervention and outcome the same for intervention and control groups or adjusted for? | Yes = 1, No = 0, Unclear = 0 |
| Q18. Were the statistical tests used to assess main outcomes appropriate? | Yes = 1, No = 0, Unclear = 0 |
| Q19. Was compliance with the interventions reliable? | Yes = 1, No = 0, Unclear = 0 |
| **INTERNAL VALIDITY (continued)** | **Yes/No/Unclear** |
| Q20. Were main outcome measures used accurate? (valid and reliable) | Yes = 1, No = 0, Unclear = 0 |
| **INTERNAL VALIDITY-CONFOUNDING (SELECTION BIAS)** | **Yes/No/Unclear** |
| Q21. Were patients in different intervention groups recruited from the same population? | Yes = 1, No = 0, Unclear = 0 |
| Q22. Were study subjects in different intervention groups recruited over the same period of time? | Yes = 1, No = 0, Unclear = 0 |
| Q23. Were study subjects randomized to intervention groups? | Yes = 1, No = 0, Unclear = 0 |
| Q24. Was the randomized intervention assignment concealed from patients and staff until recruitment was complete? | Yes = 1, No = 0, Unclear = 0 |
| Q25. Was there adequate adjustment for confounding in the analyses from which main findings were drawn? | Yes = 1, No = 0, Unclear = 0 |
| Q26. Were losses of patients to follow-up taken into account? | Yes = 1, No = 0, Unclear = 0 |
| **POWER** | **Yes/No** |
| Q27. Was the study sufficiently powered to detect clinically important effects where probability value for a difference due to chance is < 5%? | Yes = 1, No = 0 |

Qx = question number

Supplementary table 4 Downs and Black questionnaire appraisal of SLR-identified studies: Reporting subscore

| **Study** | ***D&B checklist: Reporting subscore*** | | | | | | | | | | |
| --- | --- | --- | --- | --- | --- | --- | --- | --- | --- | --- | --- |
|  | **Q1** | **Q2** | **Q3** | **Q4** | **Q5** | **Q6** | **Q7** | **Q8** | **Q9** | **Q10** | **Q1-Q10 subscore (max 11)** |
| Ageno, 2016[1] | YES | YES | YES | YES | YES | YES | YES | YES | NO | YES | ***10*** |
| Badreldin, 2018[2] | YES | YES | YES | YES | NO | YES | NO | YES | YES | NO | ***7*** |
| Bouget, 2020[3] | YES | YES | YES | YES | YES | YES | YES | YES | UTD | YES | ***10*** |
| Bounameaux, 2020[4] | YES | YES | YES | YES | YES | YES | YES | YES | YES | NO | ***10*** |
| Bryk, 2016[5] | YES | YES | YES | YES | YES | YES | YES | YES | NO | YES | ***10*** |
| Bui, 2019[6] | YES | YES | YES | YES | NO | YES | YES | YES | YES | YES | ***9*** |
| Carroll, 2018[7] | YES | YES | YES | YES | NO | NO | YES | NO | NO | UTD | ***5*** |
| Chaudhari, 2019[8] | YES | YES | NO | YES | NO | YES | NO | YES | YES | NO | ***6*** |
| Chu, 2017[9] | YES | YES | YES | YES | YES | YES | YES | YES | YES | NO | ***10*** |
| Coleman, 2017[10] | YES | YES | YES | YES | YES | YES | YES | YES | NO | NO | ***9*** |
| Coleman, 2017a [11] | YES | YES | YES | YES | YES | YES | YES | YES | YES | NO | ***10*** |
| Coleman, 2018b[12] | YES | YES | YES | YES | YES | YES | YES | YES | YES | YES | ***11*** |
| Coleman, 2018[13] | YES | YES | YES | YES | YES | YES | YES | YES | YES | YES | ***11*** |
| Coleman, 2018c[14] | YES | YES | YES | YES | YES | YES | YES | YES | YES | NO | ***10*** |
| Coleman, 2018d[15] | YES | YES | YES | YES | YES | YES | YES | YES | YES | YES | ***11*** |
| Coleman, 2018a[16] | YES | YES | YES | YES | YES | YES | YES | YES | NO | YES | ***10*** |
| Costa , 2020[17] | YES | YES | YES | YES | YES | YES | YES | YES | YES | NO | ***10*** |
| Costa , 2020a[18] | YES | YES | YES | YES | YES | YES | YES | YES | YES | NO | ***10*** |
| Dawwas, 2018[19] | YES | YES | YES | YES | NO | YES | YES | NO | NO | NO | ***6*** |
| Dawwas, 2020[20] | YES | YES | YES | YES | YES | YES | YES | YES | YES | NO | ***10*** |
| De Crema, 2015[21] | YES | YES | YES | YES | NA | YES | NO | YES | NO | YES | ***7*** |
| Desai, 2016[22] | YES | YES | YES | YES | NO | YES | YES | YES | NO | YES | ***8*** |
| Ferreira, 2020[23] | YES | YES | YES | YES | YES | YES | YES | YES | YES | YES | ***11*** |
| Fung, 2019[24] | YES | YES | YES | YES | YES | YES | YES | YES | YES | YES | ***11*** |
| Gaertner, 2017[25] | YES | YES | YES | YES | YES | YES | YES | YES | YES | YES | ***11*** |
| Goldhaber, 2020[26] | YES | YES | YES | YES | YES | YES | YES | YES | YES | NO | ***10*** |
| Gollamudi, 2018[27] | YES | YES | YES | YES | YES | YES | YES | YES | NO | YES | ***10*** |
| Guo, 2020[28] | YES | YES | YES | YES | YES | YES | YES | YES | UTD | YES | ***10*** |
| Hlavacek, 2019[29] | YES | YES | YES | YES | YES | YES | YES | YES | YES | YES | ***11*** |
| Huang, 2020[30] | YES | YES | YES | YES | YES | YES | YES | YES | YES | YES | ***11*** |
| Kohn, 2019[31] | YES | YES | YES | YES | YES | YES | YES | YES | YES | YES | ***11*** |
| Kreutz, 2019[32] | YES | YES | YES | YES | YES | YES | YES | YES | YES | YES | ***11*** |
| Krivoshchekov, 2016[33] | YES | YES | YES | YES | NO | YES | NO | YES | NO | NO | ***6*** |
| Kucher, 2016[34] | YES | YES | YES | YES | NO | YES | YES | YES | NO | YES | ***8*** |
| Kushnir, 2019[35] | YES | YES | YES | YES | YES | YES | YES | YES | YES | YES | ***11*** |
| Lai, 2016[36] | YES | YES | YES | YES | YES | YES | YES | YES | YES | YES | ***11*** |
| Larsen, 2017[37] | YES | YES | YES | YES | YES | YES | YES | YES | NO | YES | ***10*** |
| Lopez-Nunez, 2019[38] | YES | YES | YES | YES | YES | YES | YES | YES | YES | YES | ***11*** |
| Lutsey, 2018[39] | YES | YES | YES | YES | YES | YES | YES | YES | NO | NO | ***9*** |
| Lutsey, 2019[40] | YES | YES | YES | YES | YES | YES | YES | YES | YES | YES | ***11*** |
| Moustafa, 2018[41] | YES | YES | YES | YES | YES | YES | YES | YES | YES | NO | ***10*** |
| Nagaoki, 2018[42] | YES | YES | YES | YES | YES | YES | YES | YES | YES | YES | ***11*** |
| Naymagon, 2020[43] | YES | YES | YES | YES | YES | YES | YES | YES | YES | YES | ***11*** |
| Outler, 2018[44] | YES | YES | NO | YES | NO | YES | YES | YES | YES | YES | ***8*** |
| Ouyang, 2019[45] | YES | YES | NO | YES | NO | YES | YES | YES | YES | YES | ***8*** |
| Patel, 2020[46] | YES | YES | YES | YES | NO | YES | YES | YES | YES | YES | ***9*** |
| Perales, 2020[47] | YES | YES | YES | YES | YES | YES | YES | YES | YES | YES | ***11*** |
| Petrikov, 2017[48] | YES | YES | YES | YES | NO | YES | YES | YES | NO | YES | ***8*** |
| Poli, 2020[49] | YES | YES | YES | YES | YES | YES | YES | YES | YES | YES | ***11*** |
| Roetker, 2018[50] | YES | YES | YES | YES | YES | YES | YES | YES | YES | YES | ***11*** |
| Sebastian, 2018[51] | YES | YES | YES | YES | YES | YES | YES | YES | YES | YES | ***11*** |
| Sena, 2020[52] | YES | YES | YES | YES | YES | YES | YES | YES | NO | NO | ***9*** |
| Sharifi, 2015 [53] | YES | YES | YES | YES | NO | YES | YES | YES | NO | UTD | ***7*** |
| Sindet-Pedersen, 2017[54] | YES | YES | YES | YES | YES | YES | YES | YES | YES | YES | ***11*** |
| Søgaard, 2018[55] | YES | YES | YES | YES | YES | YES | YES | NO | YES | NO | ***9*** |
| Spyropoulos, 2019[56] | YES | YES | YES | YES | YES | YES | YES | YES | YES | NO | ***10*** |
| Trutyak, 2016[57] | YES | YES | YES | YES | NO | YES | YES | YES | NO | YES | ***8*** |
| Utne, 2018[58] | YES | YES | YES | YES | YES | YES | YES | NO | NO | YES | ***9*** |
| Wang, 2017[59] | YES | YES | YES | YES | YES | YES | YES | YES | YES | YES | ***11*** |
| Weeda, 2016[60] | YES | YES | YES | YES | YES | YES | YES | YES | YES | YES | ***11*** |
| Weycker, 2018[61] | YES | YES | NO | YES | YES | YES | YES | YES | NO | YES | ***9*** |
| Weycker, 2018a[62] | YES | YES | YES | YES | YES | YES | YES | YES | YES | NO | ***10*** |
| Wysokinski, 2018[63] | YES | YES | YES | YES | NA | YES | YES | YES | NO | YES | ***8*** |
| Yuko, 2018[64] | YES | YES | YES | YES | NA | YES | YES | NO | NO | YES | ***7*** |
| Zakai, 2019[65] | YES | YES | NO | YES | NO | YES | YES | YES | YES | YES | ***8*** |

Qx = question number; UTD = unable to determine; NA = not available

Supplementary table 5 Downs and Black questionnaire appraisal of SLR-identified studies: External validity subscore

| **Study** | ***D&B checklist: External validity subscore*** | | | |
| --- | --- | --- | --- | --- |
|  | **Q11** | **Q12** | **Q13** | **Q11-Q13 Subscore (max 3)** |
| Ageno, 2016[1] | YES | YES | YES | ***3*** |
| Badreldin, 2018[2] | YES | UTD | YES | ***2*** |
| Bouget, 2020[3] | YES | YES | YES | ***3*** |
| Bounameaux, 2020[4] | YES | UTD | YES | ***2*** |
| Bryk, 2016[5] | YES | NO | YES | ***2*** |
| Bui, 2019[6] | YES | UTD | YES | ***2*** |
| Carroll, 2018[7] | YES | UTD | YES | ***2*** |
| Chaudhari, 2019[8] | UTD | UTD | UTD | ***0*** |
| Chu, 2017[9] | YES | UTD | YES | ***2*** |
| Coleman, 2017[10] | YES | UTD | YES | ***2*** |
| Coleman, 2017a [11] | YES | UTD | YES | ***2*** |
| Coleman, 2018b[12] | YES | UTD | YES | ***2*** |
| Coleman, 2018[13] | YES | YES | YES | ***3*** |
| Coleman, 2018c[14] | YES | UTD | UTD | ***1*** |
| Coleman, 2018d[15] | YES | UTD | YES | ***2*** |
| Coleman, 2018a[16] | YES | YES | UTD | ***2*** |
| Costa , 2020[17] | YES | UTD | YES | ***2*** |
| Costa , 2020a[18] | YES | UTD | YES | ***2*** |
| Dawwas, 2018[19] | YES | YES | YES | ***3*** |
| Dawwas, 2020[20] | YES | UTD | YES | ***2*** |
| De Crema, 2015[21] | YES | YES | YES | ***3*** |
| Desai, 2016[22] | YES | UTD | YES | ***2*** |
| Ferreira, 2020[23] | YES | UTD | YES | ***2*** |
| Fung, 2019[24] | YES | UTD | YES | ***2*** |
| Gaertner, 2017[25] | YES | YES | YES | ***3*** |
| Goldhaber, 2020[26] | YES | UTD | YES | ***2*** |
| Gollamudi, 2018[27] | YES | UTD | YES | ***2*** |
| Guo, 2020[28] | YES | YES | YES | ***3*** |
| Hlavacek, 2019[29] | YES | UTD | YES | ***2*** |
| Huang, 2020[30] | YES | UTD | YES | ***2*** |
| Kohn, 2019[31] | YES | YES | YES | ***3*** |
| Kreutz, 2019[32] | YES | UTD | YES | ***2*** |
| Krivoshchekov, 2016[33] | YES | YES | YES | ***3*** |
| Kucher, 2016[34] | YES | UTD | YES | ***2*** |
| Kushnir, 2019[35] | YES | UTD | YES | ***2*** |
| Lai, 2016[36] | YES | UTD | YES | ***2*** |
| Larsen, 2017[37] | YES | UTD | UTD | ***1*** |
| Lopez-Nunez, 2019[38] | YES | UTD | YES | ***2*** |
| Lutsey, 2018[39] | YES | YES | YES | ***3*** |
| Lutsey, 2019[40] | YES | UTD | YES | ***2*** |
| Moustafa, 2018[41] | YES | UTD | UTD | ***1*** |
| Nagaoki, 2018[42] | YES | UTD | YES | ***2*** |
| Naymagon, 2020[43] | YES | UTD | YES | ***2*** |
| Outler, 2018[44] | UTD | UTD | UTD | ***0*** |
| Ouyang, 2019[45] | UTD | UTD | UTD | ***0*** |
| Patel, 2020[46] | YES | UTD | YES | ***2*** |
| Perales, 2020[47] | YES | UTD | YES | ***2*** |
| Petrikov, 2017[48] | NO | YES | UTD | ***1*** |
| Poli, 2020[49] | YES | UTD | YES | ***2*** |
| Roetker, 2018[50] | YES | UTD | UTD | ***1*** |
| Sebastian, 2018[51] | YES | UTD | YES | ***2*** |
| Sena, 2020[52] | YES | UTD | YES | ***2*** |
| Sharifi, 2015 [53] | NO | UTD | NO | ***0*** |
| Sindet-Pedersen, 2017[54] | YES | UTD | UTD | ***1*** |
| Søgaard, 2018[55] | YES | UTD | UTD | ***1*** |
| Spyropoulos, 2019[56] | YES | UTD | YES | ***2*** |
| Trutyak, 2016[57] | UTD | UTD | UTD | ***0*** |
| Utne, 2018[58] | UTD | YES | YES | ***2*** |
| Wang, 2017[59] | NO | UTD | UTD | ***0*** |
| Weeda, 2016[60] | YES | UTD | UTD | ***1*** |
| Weycker, 2018[61] | YES | YES | YES | ***3*** |
| Weycker, 2018a[62] | YES | UTD | YES | ***2*** |
| Wysokinski, 2018[63] | YES | YES | YES | ***3*** |
| Yuko, 2018[64] | UTD | UTD | UTD | ***0*** |
| Zakai, 2019[65] | UTD | UTD | UTD | ***0*** |

Qx = question number; UTD = unable to determine

Supplementary table 6 Downs and Black questionnaire appraisal of SLR-identified studies: Internal validity - bias subscore

| **Study** | ***D&B checklist: Internal validity - bias subscore*** | | | | | | | |
| --- | --- | --- | --- | --- | --- | --- | --- | --- |
|  | **Q14** | **Q15** | **Q16** | **Q17** | **Q18** | **Q19** | **Q20** | **Q14-Q20 subscore (max 7)** |
| Ageno, 2016[1] | NA | NA | YES | UTD | YES | YES | YES | ***4*** |
| Badreldin, 2018[2] | NA | NA | UTD | UTD | NO | YES | YES | ***2*** |
| Bouget, 2020[3] | NA | NA | UTD | NO | YES | YES | YES | ***3*** |
| Bounameaux, 2020[4] | NA | NA | UTD | NO | YES | YES | YES | ***3*** |
| Bryk, 2016[5] | NA | NA | YES | YES | YES | YES | YES | ***5*** |
| Bui, 2019[6] | NA | NA | UTD | UTD | UTD | YES | YES | ***2*** |
| Carroll, 2018[7] | NA | NA | NO | YES | YES | YES | YES | ***4*** |
| Chaudhari, 2019[8] | NA | NA | YES | UTD | YES | YES | YES | ***4*** |
| Chu, 2017[9] | NA | NA | UTD | UTD | YES | YES | YES | ***3*** |
| Coleman, 2017[10] | NA | NA | NO | UTD | YES | YES | YES | ***3*** |
| Coleman, 2017a [11] | NA | NA | UTD | UTD | YES | YES | YES | ***3*** |
| Coleman, 2018b[12] | NA | NA | UTD | UTD | YES | YES | YES | ***3*** |
| Coleman, 2018[13] | NA | NA | UTD | YES | YES | YES | YES | ***4*** |
| Coleman, 2018c[14] | NA | NA | YES | NO | YES | NO | YES | ***3*** |
| Coleman, 2018d[15] | NA | NA | UTD | UTD | YES | YES | YES | ***3*** |
| Coleman, 2018a[16] | NA | NA | YES | YES | YES | YES | YES | ***5*** |
| Costa , 2020[17] | NA | NA | UTD | UTD | YES | YES | YES | ***3*** |
| Costa , 2020a[18] | NA | NA | UTD | UTD | YES | YES | YES | ***3*** |
| Dawwas, 2018[19] | NA | NA | UTD | YES | YES | YES | YES | ***4*** |
| Dawwas, 2020[20] | NA | NA | UTD | UTD | YES | YES | YES | ***3*** |
| De Crema, 2015[21] | NA | NA | UTD | NO | YES | YES | YES | ***3*** |
| Desai, 2016[22] | NA | NA | YES | UTD | YES | YES | YES | ***4*** |
| Ferreira, 2020[23] | NA | NA | UTD | UTD | YES | YES | YES | ***3*** |
| Fung, 2019[24] | NA | NA | UTD | UTD | YES | YES | YES | ***3*** |
| Gaertner, 2017[25] | NA | NA | NO | YES | YES | YES | YES | ***4*** |
| Goldhaber, 2020[26] | NA | NA | UTD | UTD | YES | YES | YES | ***3*** |
| Gollamudi, 2018[27] | NA | NA | NO | YES | YES | YES | YES | ***4*** |
| Guo, 2020[28] | NA | NA | UTD | NO | YES | YES | YES | ***3*** |
| Hlavacek, 2019[29] | NA | NA | UTD | UTD | YES | YES | YES | ***3*** |
| Huang, 2020[30] | NA | NA | UTD | NO | YES | YES | YES | ***3*** |
| Kohn, 2019[31] | NA | NA | UTD | UTD | YES | YES | YES | ***3*** |
| Kreutz, 2019[32] | NA | NA | UTD | UTD | NO | YES | YES | ***2*** |
| Krivoshchekov, 2016[33] | NA | NA | NO | YES | YES | YES | YES | ***4*** |
| Kucher, 2016[34] | NA | NA | YES | YES | YES | YES | YES | ***5*** |
| Kushnir, 2019[35] | NA | NA | UTD | UTD | YES | YES | YES | ***3*** |
| Lai, 2016[36] | NA | NA | UTD | UTD | YES | YES | YES | ***3*** |
| Larsen, 2017[37] | NA | NA | YES | UTD | YES | YES | YES | ***4*** |
| Lopez-Nunez, 2019[38] | NA | NA | UTD | UTD | NO | YES | YES | ***2*** |
| Lutsey, 2018[39] | NA | NA | UTD | NO | YES | YES | YES | ***3*** |
| Lutsey, 2019[40] | NA | NA | UTD | UTD | YES | YES | YES | ***3*** |
| Moustafa, 2018[41] | NA | NA | YES | YES | YES | YES | YES | ***5*** |
| Nagaoki, 2018[42] | NA | NA | UTD | UTD | YES | YES | YES | ***3*** |
| Naymagon, 2020[43] | NA | NA | UTD | UTD | YES | YES | YES | ***3*** |
| Outler, 2018[44] | NA | NA | YES | UTD | YES | YES | YES | ***4*** |
| Ouyang, 2019[45] | NA | NA | YES | UTD | YES | YES | YES | ***4*** |
| Patel, 2020[46] | NA | NA | UTD | UTD | YES | YES | YES | ***3*** |
| Perales, 2020[47] | NA | NA | UTD | UTD | NO | NO | YES | ***1*** |
| Petrikov, 2017[48] | NA | NA | YES | YES | YES | YES | YES | ***5*** |
| Poli, 2020[49] | NA | NA | UTD | UTD | YES | YES | YES | ***3*** |
| Roetker, 2018[50] | NA | NA | UTD | YES | YES | YES | YES | ***4*** |
| Sebastian, 2018[51] | NA | NA | UTD | UTD | YES | YES | YES | ***3*** |
| Sena, 2020[52] | NA | NA | UTD | UTD | NO | YES | YES | ***2*** |
| Sharifi, 2015 [53] | NA | NA | NO | NO | YES | YES | YES | ***3*** |
| Sindet-Pedersen, 2017[54] | NA | NA | YES | YES | YES | NO | YES | ***4*** |
| Søgaard, 2018[55] | NA | NA | YES | YES | YES | YES | YES | ***5*** |
| Spyropoulos, 2019[56] | NA | NA | UTD | UTD | YES | YES | YES | ***3*** |
| Trutyak, 2016[57] | NA | NA | YES | YES | UTD | YES | YES | ***4*** |
| Utne, 2018[58] | NA | NA | YES | NO | YES | YES | YES | ***4*** |
| Wang, 2017[59] | NA | NA | YES | UTD | YES | YES | YES | ***4*** |
| Weeda, 2016[60] | NA | NA | YES | UTD | YES | YES | YES | ***4*** |
| Weycker, 2018[61] | NA | NA | UTD | NO | YES | YES | YES | ***3*** |
| Weycker, 2018a[62] | NA | NA | UTD | UTD | YES | YES | YES | ***3*** |
| Wysokinski, 2018[63] | NA | NA | UTD | NO | UTD | YES | YES | ***2*** |
| Yuko, 2018[64] | NA | NA | UTD | NO | YES | YES | YES | ***3*** |
| Zakai, 2019[65] | NA | NA | YES | UTD | YES | YES | YES | ***4*** |

Qx = question number; UTD = unable to determine; NA = not available

Supplementary table 7 Downs and Black questionnaire appraisal of SLR-identified studies: Internal Validity–Confounding (selection bias) subscore

| **Study** | ***D&B checklist: Internal Validity–Confounding (selection bias) subscore*** | | | | | | |
| --- | --- | --- | --- | --- | --- | --- | --- |
|  | **Q21** | **Q22** | **Q23** | **Q24** | **Q25** | **Q26** | **Q21-Q26** **subscore (max 6)** |
| Ageno, 2016[1] | NO | YES | NA | NA | YES | UTD | ***2*** |
| Badreldin, 2018[2] | UTD | YES | NA | NA | YES | UTD | ***2*** |
| Bouget, 2020[3] | YES | YES | NA | NA | YES | YES | ***4*** |
| Bounameaux, 2020[4] | UTD | YES | NA | NA | YES | UTD | ***2*** |
| Bryk, 2016[5] | YES | YES | NA | NA | YES | UTD | ***3*** |
| Bui, 2019[6] | YES | YES | NA | NA | UTD | UTD | ***2*** |
| Carroll, 2018[7] | YES | YES | NA | NA | NO | UTD | ***2*** |
| Chaudhari, 2019[8] | UTD | YES | NA | NA | NO | YES | ***2*** |
| Chu, 2017[9] | UTD | YES | NA | NA | YES | UTD | ***2*** |
| Coleman, 2017[10] | YES | YES | NA | NA | YES | UTD | ***3*** |
| Coleman, 2017a [11] | YES | YES | NA | NA | YES | YES | ***4*** |
| Coleman, 2018b[12] | UTD | YES | NA | NA | YES | UTD | ***2*** |
| Coleman, 2018[13] | YES | YES | NA | NA | YES | UTD | ***3*** |
| Coleman, 2018c[14] | YES | YES | NA | NA | YES | UTD | ***3*** |
| Coleman, 2018d[15] | UTD | YES | NA | NA | YES | UTD | ***2*** |
| Coleman, 2018a[16] | YES | YES | NO | NA | YES | NO | ***3*** |
| Costa , 2020[17] | UTD | YES | NA | NA | YES | UTD | ***2*** |
| Costa , 2020a[18] | UTD | YES | NA | NA | YES | UTD | ***2*** |
| Dawwas, 2018[19] | YES | YES | NA | NA | UTD | UTD | ***2*** |
| Dawwas, 2020[20] | UTD | YES | NA | NA | YES | UTD | ***2*** |
| De Crema, 2015[21] | YES | YES | NA | NA | UTD | UTD | ***2*** |
| Desai, 2016[22] | YES | YES | NA | NA | YES | UTD | ***3*** |
| Ferreira, 2020[23] | NO | YES | NA | NA | YES | YES | ***3*** |
| Fung, 2019[24] | UTD | YES | NA | NA | YES | UTD | ***2*** |
| Gaertner, 2017[25] | NO | NO | NA | NA | YES | YES | ***2*** |
| Goldhaber, 2020[26] | UTD | YES | NA | NA | YES | UTD | ***2*** |
| Gollamudi, 2018[27] | NO | UTD | NA | NA | YES | UTD | ***1*** |
| Guo, 2020[28] | YES | YES | NA | NA | YES | UTD | ***3*** |
| Hlavacek, 2019[29] | UTD | YES | NA | NA | YES | UTD | ***2*** |
| Huang, 2020[30] | YES | YES | YES | NA | UTD | YES | ***4*** |
| Kohn, 2019[31] | YES | YES | NA | NA | YES | UTD | ***3*** |
| Kreutz, 2019[32] | UTD | YES | NA | NA | YES | UTD | ***2*** |
| Krivoshchekov, 2016[33] | UTD | YES | NA | NA | NO | UTD | ***1*** |
| Kucher, 2016[34] | YES | YES | NA | NA | YES | UTD | ***3*** |
| Kushnir, 2019[35] | UTD | YES | NA | NA | YES | UTD | ***2*** |
| Lai, 2016[36] | UTD | YES | NA | NA | YES | UTD | ***2*** |
| Larsen, 2017[37] | YES | YES | NA | NA | YES | YES | ***4*** |
| Lopez-Nunez, 2019[38] | UTD | YES | NA | NA | YES | UTD | ***2*** |
| Lutsey, 2018[39] | YES | YES | NA | NA | UTD | UTD | ***2*** |
| Lutsey, 2019[40] | UTD | YES | NA | NA | YES | UTD | ***2*** |
| Moustafa, 2018[41] | NO | YES | NA | NA | YES | YES | ***3*** |
| Nagaoki, 2018[42] | UTD | YES | NA | NA | YES | UTD | ***2*** |
| Naymagon, 2020[43] | UTD | YES | NA | NA | YES | UTD | ***2*** |
| Outler, 2018[44] | UTD | YES | NA | NA | NO | YES | ***2*** |
| Ouyang, 2019[45] | UTD | YES | NA | NA | NO | YES | ***2*** |
| Patel, 2020[46] | YES | YES | NA | NA | YES | YES | ***4*** |
| Perales, 2020[47] | UTD | YES | NA | NA | NO | UTD | ***1*** |
| Petrikov, 2017[48] | YES | YES | NA | NA | NO | UTD | ***2*** |
| Poli, 2020[49] | UTD | YES | NA | NA | YES | UTD | ***2*** |
| Roetker, 2018[50] | YES | YES | NA | NA | YES | UTD | ***3*** |
| Sebastian, 2018[51] | UTD | YES | NA | NA | YES | UTD | ***2*** |
| Sena, 2020[52] | UTD | YES | NA | NA | NO | UTD | ***1*** |
| Sharifi, 2015 [53] | YES | YES | NA | NA | NO | UTD | ***2*** |
| Sindet-Pedersen, 2017[54] | YES | YES | NA | NA | YES | YES | ***4*** |
| Søgaard, 2018[55] | YES | YES | NA | NA | YES | YES | ***4*** |
| Spyropoulos, 2019[56] | UTD | YES | NA | NA | YES | UTD | ***2*** |
| Trutyak, 2016[57] | UTD | UTD | NA | NA | UTD | UTD | ***0*** |
| Utne, 2018[58] | NO | YES | NA | NA | YES | UTD | ***2*** |
| Wang, 2017[59] | YES | YES | NA | NA | YES | YES | ***4*** |
| Weeda, 2016[60] | YES | YES | NA | NA | YES | YES | ***4*** |
| Weycker, 2018[61] | YES | YES | NA | NA | UTD | UTD | ***2*** |
| Weycker, 2018a[62] | UTD | YES | NA | NA | YES | UTD | ***2*** |
| Wysokinski, 2018[63] | YES | YES | NA | NA | UTD | UTD | ***2*** |
| Yuko, 2018[64] | YES | UTD | NA | NA | UTD | UTD | ***1*** |
| Zakai, 2019[65] | UTD | YES | NA | NA | YES | YES | ***3*** |

Qx = question number; UTD = unable to determine; NA = not available

Supplementary table 8 Downs and Black questionnaire appraisal of SLR-identified studies: Power subscore and total score

| **Study** | ***D&B checklist*** | |
| --- | --- | --- |
|  | **Q27 Power** | **Q1-Q27 Total score (max 28)** |
| Ageno, 2016[1] | NA | ***19*** |
| Badreldin, 2018[2] | NA | ***13*** |
| Bouget, 2020[3] | YES | ***21*** |
| Bounameaux, 2020[4] | NA | ***17*** |
| Bryk, 2016[5] | NA | ***20*** |
| Bui, 2019[6] | NA | ***15*** |
| Carroll, 2018[7] | NA | ***13*** |
| Chaudhari, 2019[8] | NA | ***12*** |
| Chu, 2017[9] | NA | ***17*** |
| Coleman, 2017[10] | NA | ***17*** |
| Coleman, 2017a [11] | NA | ***19*** |
| Coleman, 2018b[12] | NA | ***18*** |
| Coleman, 2018[13] | NA | ***21*** |
| Coleman, 2018c[14] | NA | ***17*** |
| Coleman, 2018d[15] | NA | ***18*** |
| Coleman, 2018a[16] | NA | ***20*** |
| Costa , 2020[17] | NA | ***17*** |
| Costa , 2020a[18] | NA | ***17*** |
| Dawwas, 2018[19] | UTD | ***15*** |
| Dawwas, 2020[20] | NA | ***17*** |
| De Crema, 2015[21] | YES | ***16*** |
| Desai, 2016[22] | NA | ***17*** |
| Ferreira, 2020[23] | NA | ***19*** |
| Fung, 2019[24] | NA | ***18*** |
| Gaertner, 2017[25] | NA | ***20*** |
| Goldhaber, 2020[26] | NA | ***17*** |
| Gollamudi, 2018[27] | NA | ***17*** |
| Guo, 2020[28] | NA | ***19*** |
| Hlavacek, 2019[29] | NA | ***18*** |
| Huang, 2020[30] | UTD | ***20*** |
| Kohn, 2019[31] | NA | ***20*** |
| Kreutz, 2019[32] | NA | ***17*** |
| Krivoshchekov, 2016[33] | NA | ***14*** |
| Kucher, 2016[34] | NA | ***18*** |
| Kushnir, 2019[35] | NA | ***18*** |
| Lai, 2016[36] | NA | ***18*** |
| Larsen, 2017[37] | NA | ***19*** |
| Lopez-Nunez, 2019[38] | NA | ***17*** |
| Lutsey, 2018[39] | UTD | ***17*** |
| Lutsey, 2019[40] | NA | ***18*** |
| Moustafa, 2018[41] | NA | ***19*** |
| Nagaoki, 2018[42] | NA | ***18*** |
| Naymagon, 2020[43] | NA | ***18*** |
| Outler, 2018[44] | NA | ***14*** |
| Ouyang, 2019[45] | NA | ***14*** |
| Patel, 2020[46] | NA | ***18*** |
| Perales, 2020[47] | NA | ***15*** |
| Petrikov, 2017[48] | NA | ***16*** |
| Poli, 2020[49] | NA | ***18*** |
| Roetker, 2018[50] | NA | ***19*** |
| Sebastian, 2018[51] | NA | ***18*** |
| Sena, 2020[52] | NA | ***14*** |
| Sharifi, 2015 [53] | NA | ***12*** |
| Sindet-Pedersen, 2017[54] | NA | ***20*** |
| Søgaard, 2018[55] | NA | ***19*** |
| Spyropoulos, 2019[56] | NA | ***17*** |
| Trutyak, 2016[57] | NA | ***12*** |
| Utne, 2018[58] | NA | ***17*** |
| Wang, 2017[59] | NA | ***19*** |
| Weeda, 2016[60] | NA | ***20*** |
| Weycker, 2018[61] | UTD | ***17*** |
| Weycker, 2018a[62] | NA | ***17*** |
| Wysokinski, 2018[63] | UTD | ***15*** |
| Yuko, 2018[64] | UTD | ***11*** |
| Zakai, 2019[65] | NA | ***15*** |

Qx = question number; UTD = unable to determine; NA = not available

Supplementary figure 3 Summary of Downs and Black questionnaire appraisal of SLR-identified studies.


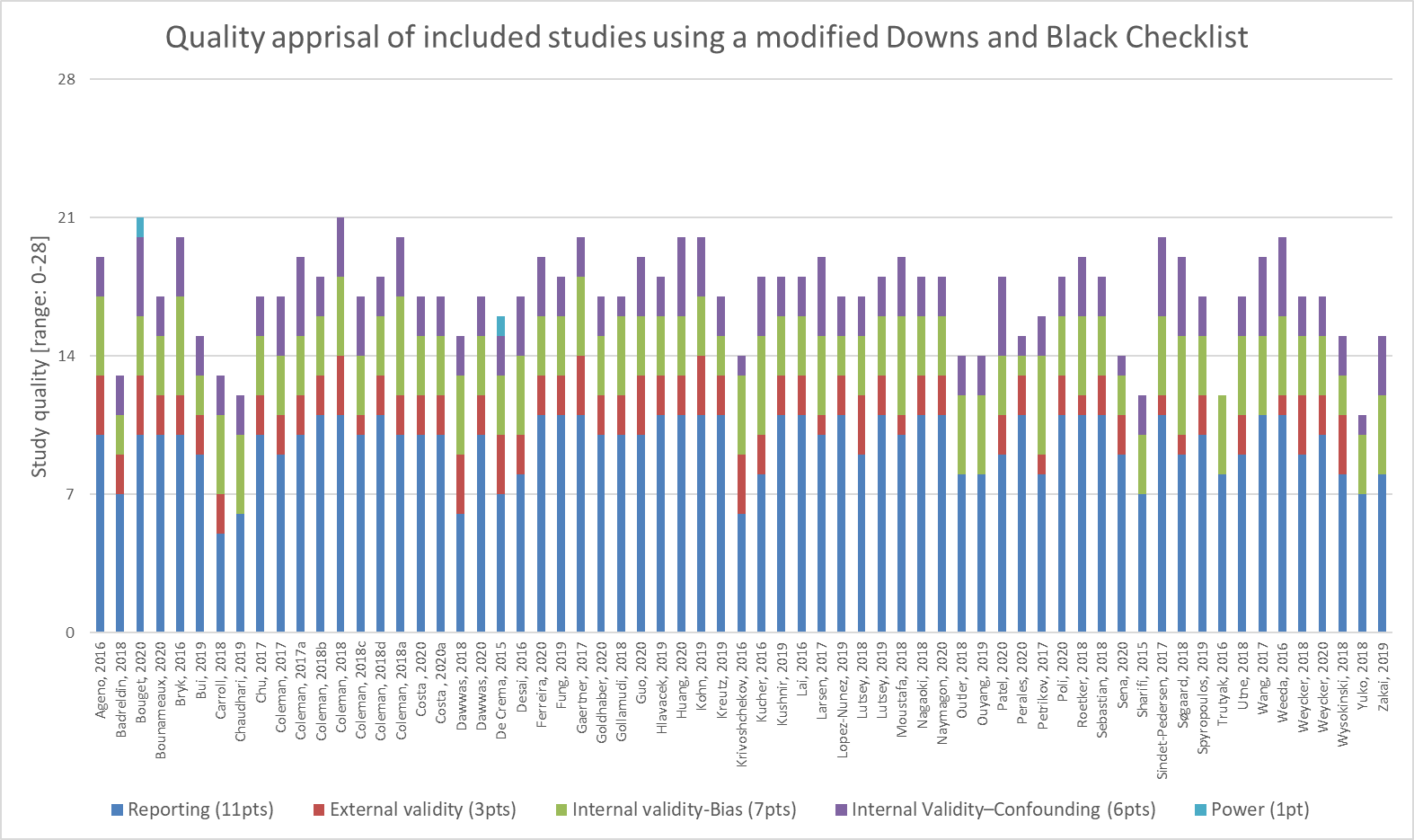


Supplementary table 9 Bias assessment of studies selected after database overlapping analysis

| **Study** | **HR reported/ imputed** | **HR adjustment method** | **Imputation method** |
| --- | --- | --- | --- |
| Ageno, 2016[1] | rep./imp. | PSM | N events with equal FU time |
| Badreldin, 2018[2] | imp. |  | N events with equal FU time |
| Bouget, 2020[3] | rep. | Cox proportional hazard regression models unadjusted and adjusted on known patient characteristics |  |
| Bryk, 2016[5] | imp. |  | N events with equal FU time |
| Bui, 2019[6] | imp. |  | N events with equal FU time |
| Carroll, 2018[7] | imp. |  | N events with equal FU time |
| Chaudhari, 2019[8] | imp. |  | N events with equal FU time |
| Coleman, 2017[10] | rep. | Cox proportion hazards regression models with IPTW adjustment |  |
| Coleman, 2018[13] | rep. | PSM |  |
| Coleman, 2018a [16] | rep. | PSM |  |
| Costa , 2020[17] | rep. | PSM |  |
| Costa , 2020a[18] | rep. | PSM |  |
| Dawwas, 2020[20] | rep. | PSM |  |
| Desai, 2016[22] | imp. |  | N events with equal FU time; correction for zero events |
| Fung, 2019[24] | imp. |  | N events with equal FU time |
| Gaertner, 2017[25] | rep./imp. | PSM | N events with equal FU time |
| Hlavacek, 2019[29] | rep. | PSM |  |
| Huang, 2020[30] | imp. |  | N events with equal FU time (Missing FU assumption: 6mo) |
| Kohn, 2019[31] | rep. | PSM |  |
| Kreutz, 2019[32] | imp. |  | N events with unequal FU (FU estimated from median, IQR) |
| Krivoshchekov, 2016[33] | imp. |  | N events with equal FU time |
| Kucher, 2016[34] | rep. | PSM |  |
| Kushnir, 2019[35] | imp. |  | N events with unequal FU (FU estimated from median, IQR) |
| Lai, 2016[36] | imp. |  | N events with unequal FU mean |
| Larsen, 2017[37] | rep. | PSM |  |
| Moustafa, 2018[41] | rep./imp. | Adjusted multivariate model | HR from rate 100 person-years, assuming observation horizon based on Mean days of therapy, and FU >6 months based on Median days (IQR) |
| Patel, 2020[46] | imp. |  | N events with equal FU time |
| Perales, 2020[47] | imp. |  | N events with equal FU time |
| Roetker, 2018[50] | rep. | IPTW MSM |  |
| Sebastian, 2018[51] | imp. |  | N events with unequal FU mean |
| Sena, 2020[52] | rep. | ― |  |
| Sindet-Pedersen, 2017[54] | rep. | Adjusted Cox-regression |  |
| Søgaard, 2018[55] | rep. | PSM |  |
| Trutyak, 2016[57] | imp. |  | N events with equal FU time; correction for zero events |
| Wang, 2017[59] | imp. |  | N events with equal FU time |
| Weycker, 2018a[62] | rep. | PSM |  |
| Wysokinski, 2018[63] | rep. | NA |  |
| Zakai, 2019[65] | rep. | PSM |  |

Rep. = reported; imp. = imputed; FU = follow-up; PSM = propensity score matching; IPTW = inverse probability treatment weighting; MSM = marginal structural model; NA = not available

Supplementary table 10 Meta-analysis inputs for recurrent venous thromboembolism for RIV in VTE

| **Study** | **RIV** | | | **Comparator** | | | | **Database** | **HR [95% CI]** | **Scenario** | | | | | | |
| --- | --- | --- | --- | --- | --- | --- | --- | --- | --- | --- | --- | --- | --- | --- | --- | --- |
|  | **Events (n)** | **N** | **FU (months)** | **Class** | **Events (n)** | **N** | **FU (months)** |  |  | **BC** | **S1** | **S2** | **S3** | **S4** | **S5** | **S6** |
| Coleman, 2018a [16] |  | 10,489 | 6 | VKA± Hep |  | 26,364 | 6 | MarketScan | **0.60 [0.54, 0.67]** | **✓** | **✓** | **✓** |  | **✓** |  |  |
| Coleman, 2018a [16] |  | 10,489 | 3 | VKA± Hep |  | 26,364 | 3 | MarketScan | **0.61 [0.54, 0.68]** |  |  |  |  |  |  |  |
| Coleman, 2018a [16] |  | 10,489 | 12 (5 ^a^) | VKA± Hep |  | 26,364 | 12 (5 ^a^) | MarketScan | **0.53 [0.47, 0.61]** |  |  |  |  |  |  |  |
| Costa, 2021 [17] |  | 6,755 | 12 | VKA± Hep |  | 6,755 | 12 | Optum | **0.63 [0.54, 0.74]** | **✓** | **✓** | **✓** |  |  | **✓** | **✓** |
| Costa, 2021 [17] |  | 6,755 | 6 | VKA± Hep |  | 6,755 | 6 | Optum | **0.65 [0.55, 0.77]** |  |  |  |  | **✓** |  |  |
| Coleman, 2018 [13] |  | 4,454 | 6 | VKA± Hep |  | 13,164 | 6 | MarketScan | **0.71 [0.60, 0.84]** | **✓** | **✓** | **✓** |  | **✓** |  |  |
| Costa, 2021 [17] |  | 6,755 | 3 | VKA± Hep |  | 6,755 | 3 | Optum | **0.61 [0.51, 0.72]** |  |  |  |  |  |  |  |
| Coleman, 2018 [13] |  | 4,454 | 3 | VKA± Hep |  | 13,164 | 3 | MarketScan | **0.70 [0.59, 0.84]** |  |  |  |  |  |  |  |
| Coleman, 2018 [13] |  | 4,454 | 12 (4.6 ^a^) | VKA± Hep |  | 13,164 | 12 (4.6 ^a^) | MarketScan | **0.68 [0.56, 0.82]** |  |  |  |  |  |  |  |
| Sogaard, 2018 [55] |  | 8,567 | 6 | VKA± Hep |  | 11,390 | 6 | Danish nationwide registries | 0.90 [0.74, 1.09] | **✓** | **✓** | **✓** |  | **✓** |  |  |
| Sogaard, 2018 [55] |  | 8,567 | 36 | VKA± Hep |  | 11,390 | 36 | Danish nationwide registries | 0.96 [0.83, 1.23] |  |  |  |  |  | **✓** | **✓** |
| Moustafa, 2018 [41] | 22 | 2,348 | 6 | VKA± Hep | 97 | 8,059 | 6 | RIETE | 0.78 [0.49, 1.24] |  |  | **✓** | **✓** |  |  | **✓** |
| Ageno, 2016 [1] | 36 | 2,505 | ≥12 (5.9 ^a^) | VKA± Hep | 47 | 2,010 | ≥12 (5.9 ^a^) | XALIA trial | 0.91 [0.54, 1.54] | **✓** | **✓** | **✓** | **✓** |  |  |  |
| Sena, 2020 [52] |  | 134 | 108 | VKA± Hep | 42 | 312 | 108 | Single-centre (Istanbul) | 0.83 [0.45, 1.56] | **✓** | **✓** | **✓** |  |  | **✓** | **✓** |
| Kreutz, 2019 [32] | 18 | 1,285 | 7 (5 ^a^) | VKA± Hep | 16 | 402 | 7(5 ^a^) | XALIA-LEA trial | **0.33 [0.17, 0.64]#** | **✓** |  | **✓** | **✓** |  |  |  |
| Wang, 2017 [59] | 6 | 203 | 3 | VKA± Hep | 32 | 609 | 3 | US Dept. VA | 0.56 [0.24, 1.35]# | **✓** |  | **✓** | **✓** | **✓** |  |  |
| Sebastian, 2018 [51] | 8 | 73 | 22 (5.9 ^a^) | VKA± Hep | 7 | 38 | 31 (5.9 ^a^) | Swiss Venous Stent registry | 0.84 [0.30, 2.31]# | **✓** |  | **✓** | **✓** |  |  |  |
| Kucher, 2016 [34] |  | 417 | 3 | VKA± Hep |  | 417 | 3 | SWIVTER | 0.55 [0.18, 1.65] | **✓** | **✓** | **✓** | **✓** | **✓** |  |  |
| Bryk, 2016 [5] | 8 | 76 | 13 | VKA± Hep | 3 | 45 | 13 | John Paul II Hospital (Cracow) | 1.58 [0.42, 5.95]# | **✓** |  | **✓** |  |  | **✓** | **✓** |
| Chaudhari, 2019 [8] | 3 | 58 | 6 | VKA± Hep | 5 | 81 | 6 | Single-centre (Bangalore) | 0.84 [0.20, 3.51]# | **✓** |  | **✓** | **✓** | **✓** |  |  |
| Bui, 2019 [6] | 3 | 83 | 6 | VKA± Hep | 5 | 104 | 6 | Cho Ray Hospital | 0.75 [0.18, 3.15]# | **✓** |  | **✓** | **✓** | **✓** |  |  |
| Gaertner, 2017 [25] | 4 | 280 | 6 | VKA± Hep | 3 | 96 | 6 | REMOTEV | 0.46 [0.10, 2.04]# |  |  | **✓** | **✓** |  |  |  |
| Moustafa, 2018 [41] | 1 | 151 | 5 | VKA± Hep | 23 | 939 | 5 | RIETE | 0.39 [0.06, 1.40] |  |  | **✓** | **✓** |  |  |  |
| Fung, 2019 [24] | 2 | 84 | 12 | VKA± Hep | 6 | 82 | 12 | Single-centre (Hong Kong) | 0.33 [0.07, 1.61]# | **✓** |  | **✓** | **✓** |  | **✓** | **✓** |
| Perales, 2020 [47] | 2 | 47 | 12 | VKA± Hep | 4 | 62 | 12 | Two medical centres (Arizona) | 0.66 [0.12, 3.60]# | **✓** |  | **✓** |  |  | **✓** | **✓** |
| Kushnir, 2019 [35] | 3 | 152 | 9 | VKA± Hep | 2 | 167 | 8 | Clinical Looking Glass | 1.61 [0.27, 9.62]# | **✓** |  | **✓** |  |  | **✓** | **✓** |
| Badreldin, 2018 [2] | 1 | 92 | 6 | VKA± Hep | 5 | 180 | 6 | Single-centre (Boston) | 0.39 [0.05, 3.35]# | **✓** |  | **✓** |  | **✓** |  |  |
| Krivoshchekov, 2016 [33] | 1 | 46 | 3 | VKA± Hep | 3 | 78 | 3 | Single-centre (Samara) | 0.57 [0.06, 5.43]# | **✓** |  | **✓** | **✓** | **✓** |  |  |
| Moustafa, 2018 [41] | 22 | 2,348 | 6 | Hep | 72 | 3,384 | 6 | RIETE | **0.44 [0.27, 0.71]** |  |  |  |  |  |  |  |
| Moustafa, 2018 [41] | 22 | 2,348 | 6 | Hep | 182 | 11,443 | 6 | RIETE | 0.59 [0.30, 1.17]$ | **✓** |  |  |  |  | **✓** |  |
| Gaertner, 2017 [25] | 4 | 280 | 6 | Hep | 8 | 69 | 6 | REMOTEV | **0.12 [0.04, 0.41]#** |  |  |  |  |  |  |  |
| Moustafa, 2018 [41] | 1 | 151 | 5 | Hep | 79 | 2,071 | 5 | RIETE | **0.25 [0.04, 0.84]** |  |  |  |  |  |  |  |
| Moustafa, 2018 [41] | 1 | 151 | 5 | Hep | 93 | 3,010 | 5 | RIETE | 0.30 [0.06, 1.45]$ | **✓** |  |  |  | **✓** |  |  |
| Gaertner, 2017 [25] | 4 | 280 | 6 | Hep | 11 | 165 | 6 | REMOTEV | 0.22 [0.04, 1.09]$ | **✓** |  |  |  | **✓** |  |  |

BC = base-case scenario; CI = confidence intervals; FU = follow-up; HR = hazard ratio; Hep = heparin; N = number of patients; RIV = rivaroxaban; S1 = scenario 1 (only HRs reported); S2 = scenario 2 (only VKA±Hep); S3 = scenario 3 (only VKA+Hep at any stage); S4 = scenario 4 (FU ≤6mo); S5 = scenario 5 (FU >6mo); S6 = scenario 6 (only VKA±Hep with FU >6mo); VKA±Hep = vitamin K antagonist ±heparins; VTE = Venous thromboembolism. Green colour indicates result favourable for rivaroxaban. Magenta indicates result favourable for comparator. **Bold** means that the result is significant.

^a^ – mean treatment duration

# - HR [95%CI] estimated based on available information about incidence rates or binary data

$ - HR [95%CI] estimated in two-step MA method approach for 3-arm studies

Supplementary figure 4 Meta-analysis results for recurrent venous thromboembolism for RIV in VTE


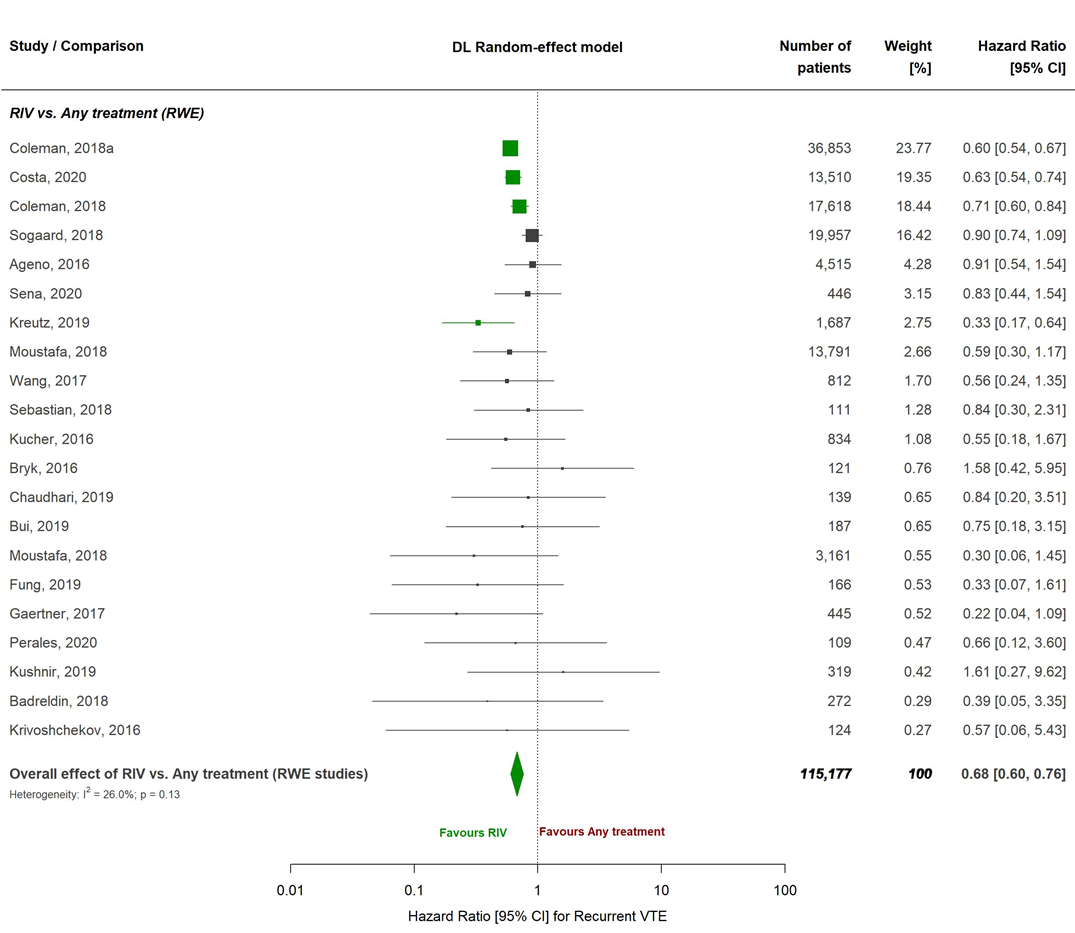


CI = confidence intervals; DL random effect model = DerSimonian-Laird random effects model; *I^2^* = I square statistic; p = p-value; RIV = rivaroxaban; RWE = real world evidence; VTE = Venous thromboembolism.

Supplementary table 11 Meta-analysis inputs for recurrent pulmonary embolism for RIV in VTE

| **Study** | **Rivaroxaban** | | | **Comparator** | | | | **Database** | **HR [95% CI]** | **Scenario** | | | | | | |
| --- | --- | --- | --- | --- | --- | --- | --- | --- | --- | --- | --- | --- | --- | --- | --- | --- |
|  | **Events (n)** | **N** | **FU**  **(months)** | **Class** | **Events (n)** | **N** | **FU**  **(months)** |  |  | **BC** | **S1** | **S2** | **S3** | **S4** | **S5** | **S6** |
| Sindet-Pedersen, 2017 [54] | 94 | 7,572 | 6 | VKA± Hep | 177 | 10,844 | 6 | Danish nationwide registries | 0.82 [0.62, 1.08] | **✓** | **✓** | **✓** |  | **✓** |  |  |
| Ageno, 2016 [1] | 19 | 2,619 | 16 (5.9 ^a^) | VKA± Hep | 22 | 2,149 | 16 (5.9 ^a^) | XALIA trial | 0.71 [0.38, 1.31]# | **✓** |  | **✓** | **✓** |  |  |  |
| Kreutz, 2019 [32] | 4 | 1,285 | 7 (5 ^a^) | VKA± Hep | 12 | 402 | 7 (5 ^a^) | XALIA-LEA trial | **0.10 [0.03, 0.30]#** | **✓** |  | **✓** | **✓** |  |  |  |
| Gaertner, 2017 [25] | 3 | 280 | 6 | VKA± Hep | 1 | 96 | 6 | REMOTEV | 1.03 [0.11, 9.89]# |  |  | **✓** | **✓** |  |  |  |
| Bui, 2019 [6] | 1 | 83 | 6 | VKA± Hep | 1 | 104 | 6 | Cho Ray Hospital | 1.25 [0.08, 20.03]# | **✓** |  | **✓** | **✓** | **✓** |  |  |
| Gaertner, 2017 [25] | 3 | 280 | 6 | Hep | 7 | 69 | 6 | REMOTEV | **0.11 [0.03, 0.41]#** |  |  |  |  |  |  |  |
| Gaertner, 2017 [25] | 3 | 280 | 6 | Hep | 6 | 165 | 6 | REMOTEV | 0.27 [0.02, 3.24]$ | **✓** |  |  |  | **✓** |  |  |

BC = base-case scenario; CI = confidence intervals; FU = follow-up; HR = hazard ratio; Hep = heparin; N = number of patients; RIV = rivaroxaban; S1 = scenario 1 (only HRs reported); S2 = scenario 2 (only VKA±Hep); S3 = scenario 3 (only VKA+Hep at any stage); S4 = scenario 4 (FU ≤6mo); S5 = scenario 5 (FU >6mo); S6 = scenario 6 (only VKA±Hep with FU >6mo); VKA±Hep = vitamin K antagonist ±heparins; VTE = Venous thromboembolism.

Green colour indicates result favourable for rivaroxaban. Magenta indicates result favourable for comparator. **Bold** means that the result is significant.

^a^ – mean treatment duration

# - HR [95%CI] estimated based on available information about incidence rates or binary data

$ - HR [95%CI] estimated in two-step MA method approach for 3-arm studies

Supplementary figure 5 Meta-analysis results for recurrent pulmonary embolism for RIV in VTE

**
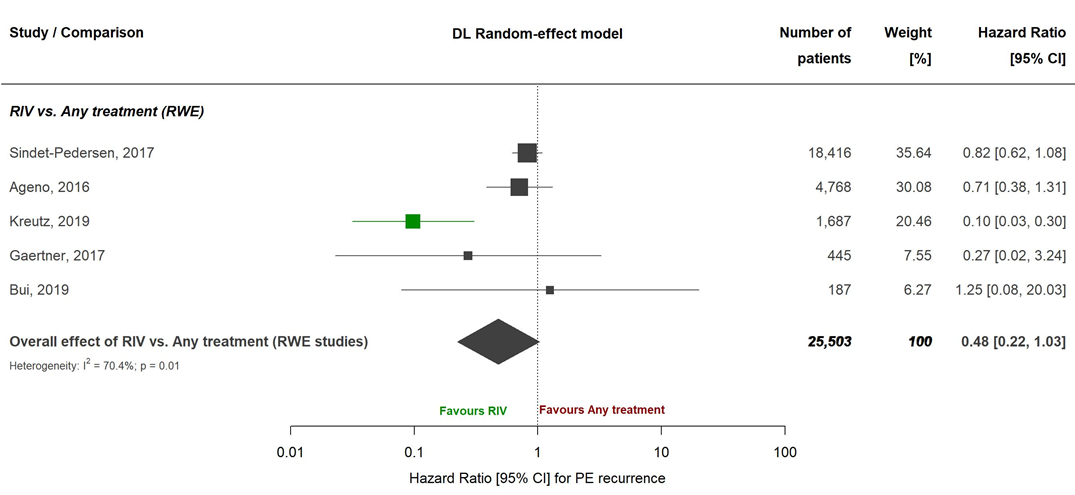
**

Abbreviations: RWE – real world evidence, DL random effect model – DerSimonian-Laird random effects model, *I^2^ –* I square statistic, CI – confidence intervals, RIV – rivaroxaban, p - p-value.

Supplementary table 12 Meta-analysis inputs for recurrent deep vein thrombosis for RIV in VTE

| **Study** | **Rivaroxaban** | | | **Comparator** | | | | **Database** | **HR [95% CI]** | **Scenario** | | | | | | |
| --- | --- | --- | --- | --- | --- | --- | --- | --- | --- | --- | --- | --- | --- | --- | --- | --- |
|  | **Events (n)** | **N** | **FU (months)** | **Class** | **Events (n)** | **N** | **FU (months)** |  |  | **BC** | **S1** | **S2** | **S3** | **S4** | **S5** | **S6** |
| Sindet-Pedersen, 2017 [54] | 113 | 7,572 | 6 | VKA± Hep | 224 | 10,844 | 6 | Danish nationwide registries | 0.98 [0.78, 1.26] | **✓** | **✓** | **✓** |  | **✓** |  |  |
| Ageno, 2016 [1] | 14 | 2,619 | 16 (5.9 ^a^) | VKA± Hep | 34 | 2,149 | 16 (5.9 ^a^) | XALIA trial | **0.34 [0.18, 0.63]#** | **✓** |  | **✓** | **✓** |  |  |  |
| Kreutz, 2019 [32] | 14 | 1,285 | 7 (5 ^a^) | VKA± Hep | 4 | 402 | 7 (5 ^a^) | XALIA-LEA trial | 1.03 [0.34, 3.12]# | **✓** |  | **✓** | **✓** |  |  |  |
| Fung, 2019 [24] | 2 | 84 | 12 | VKA± Hep | 6 | 82 | 12 | Single-center (Hong Kong) | 0.33 [0.07, 1.61]# | **✓** |  | **✓** | **✓** |  | **✓** | **✓** |
| Bui, 2019 [6] | 2 | 83 | 6 | VKA± Hep | 3 | 104 | 6 | Cho Ray Hospital | 0.84 [0.14, 5.00]# | **✓** |  | **✓** | **✓** | **✓** |  |  |
| Gaertner, 2017 [25] | 1 | 280 | 6 | VKA± Hep | 2 | 96 | 6 | REMOTEV | 0.17 [0.02, 1.89]# |  |  | **✓** | **✓** |  |  |  |
| Gaertner, 2017 [25] | 1 | 280 | 6 | Hep | 3 | 165 | 6 | REMOTEV | 0.19 [0.02, 1.86]$ | **✓** |  |  |  | **✓** |  |  |
| Gaertner, 2017 [25] | 1 | 280 | 6 | Hep | 1 | 69 | 6 | REMOTEV | 0.25 [0.02, 3.94]# |  |  |  |  |  |  |  |

BC = base-case scenario; CI = confidence intervals; FU = follow-up; HR = hazard ratio; Hep = heparin; N = number of patients; RIV = rivaroxaban; S1 = scenario 1 (only HRs reported); S2 = scenario 2 (only VKA±Hep); S3 = scenario 3 (only VKA+Hep at any stage); S4 = scenario 4 (FU ≤6mo); S5 = scenario 5 (FU >6mo); S6 = scenario 6 (only VKA±Hep with FU >6mo); VKA±Hep = vitamin K antagonist ±heparins; VTE = Venous thromboembolism. Green colour indicates result favourable for rivaroxaban. Magenta indicates result favourable for comparator. **Bold** means that the result is significant.

^a^ – mean treatment duration

# - HR [95%CI] estimated based on available information about incidence rates or binary data

$ - HR [95%CI] estimated in two-step MA method approach for 3-arm studies

Supplementary figure 6 Meta-analysis results for recurrent deep vein thrombosis for RIV in VTE

**
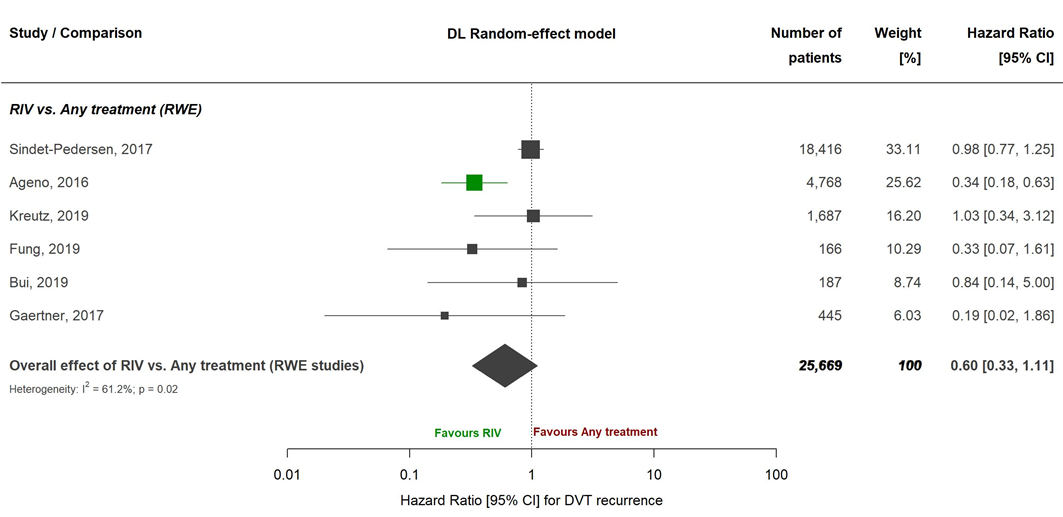
**

CI = confidence intervals; DL random effect model = DerSimonian-Laird random effects model; I2 = I square statistic; p = p-value; RIV = rivaroxaban; RWE = real world evidence; VTE = Venous thromboembolism; Green marks represent results indicating statistically lower event rate in the RIV group.

Supplementary table 13 Meta-analysis inputs for all-cause mortality for RIV in VTE

| **Study** | **RIV** | | | **Comparator** | | | | **Database** | **HR [95% CI]** | **Scenario** | | | | | | |
| --- | --- | --- | --- | --- | --- | --- | --- | --- | --- | --- | --- | --- | --- | --- | --- | --- |
|  | **Events (n)** | **N** | **FU (months)** | **Class** | **Events (n)** | **N** | **FU (months)** |  |  | **BC** | **S1** | **S2** | **S3** | **S4** | **S5** | **S6** |
| Roetker, 2018 [50] | 428 | 21,064 | 6 | VKA± Hep | 1,208 | 35,704 | 6 | OptumLabs | 0.97 [0.84, 1.11] | **✓** | **✓** | **✓** |  | **✓** |  |  |
| Larsen, 2017 [37] |  | 1,734 | 6 | VKA± Hep |  | 2,945 | 6 | Danish nationwide registries | 1.03 [0.72, 1.49] | **✓** | **✓** | **✓** | **✓** | **✓** |  |  |
| Kreutz, 2019 [32] | 29 | 1,285 | 7 (5^a^) | VKA± Hep | 29 | 402 | 7 (5 ^a^) | XALIA-LEA trial | **0.29 [0.18, 0.49]#** | **✓** |  | **✓** | **✓** |  |  |  |
| Sena, 2020 [52] | 13 | 134 | 108 | VKA± Hep | 57 | 312 | 108 | Single-centre (Istanbul) | 0.62 [0.33, 1.12] | **✓** | **✓** | **✓** |  |  | **✓** | **✓** |
| Ageno, 2016 [1] | 11 | 2,505 | ≥12 (5.9 ^a^) | VKA± Hep | 69 | 2,010 | ≥12 (5.9 ^a^) | XALIA trial | 0.51 [0.24, 1.07] | **✓** | **✓** | **✓** | **✓** |  |  |  |
| Wang, 2017 [59] | 5 | 203 | 3 | VKA± Hep | 25 | 609 | 3 | US Dept. VA | 0.60 [0.23, 1.57]# | **✓** |  | **✓** | **✓** | **✓** |  |  |
| Gaertner, 2017 [25] | 5 | 280 | 6 | VKA± Hep | 5 | 96 | 6 | REMOTEV | **0.21 [0.06, 0.66]** |  |  | **✓** | **✓** |  |  |  |
| Huang, 2020 [30] | 3 | 86 | 6 | VKA± Hep | 4 | 42 | 6 | Single-centre (Guangzhou) | 0.37 [0.08, 1.64]# | **✓** |  | **✓** | **✓** | **✓** |  |  |
| Perales, 2020 [47] | 2 | 47 | 12 | VKA± Hep | 5 | 62 | 12 | Two medical centres (Arizona) | 0.53 [0.10, 2.72]# | **✓** |  | **✓** |  |  | **✓** | **✓** |
| Bui, 2019 [6] | 2 | 83 | 6 | VKA± Hep | 4 | 104 | 6 | Cho Ray Hospital | 0.63 [0.11, 3.42]# | **✓** |  | **✓** | **✓** | **✓** |  |  |
| Wysokinski, 2018 [63] | 28 | 293 | N.R. | Hep | 47 | 251 | N.R. | Mayo TCA Registry | **0.39 [0.24, 0.62]** | **✓** | **✓** |  |  |  |  |  |
| Gaertner, 2017 [25] | 5 | 280 | 6 | Hep | 14 | 69 | 6 | REMOTEV | **0.09 [0.03, 0.24]#** |  |  |  |  |  |  |  |
| Gaertner, 2017 [25] | 5 | 280 | 6 | Hep | 18 | 165 | 6 | REMOTEV | **0.17 [0.03, 0.81]$** | **✓** |  |  |  | **✓** |  |  |

BC = base-case scenario; CI = confidence intervals; FU = follow-up; HR = hazard ratio; Hep = heparin; N = number of patients; RIV = rivaroxaban; S1 = scenario 1 (only HRs reported); S2 = scenario 2 (only VKA±Hep); S3 = scenario 3 (only VKA+Hep at any stage); S4 = scenario 4 (FU ≤6mo); S5 = scenario 5 (FU >6mo); S6 = scenario 6 (only VKA±Hep with FU >6mo); VKA±Hep = vitamin K antagonist ±heparins; VTE = Venous thromboembolism.

Green colour indicates result favourable for rivaroxaban. Magenta indicates result favourable for comparator. **Bold** means that the result is significant.

^a^ – mean treatment duration

# - HR [95%CI] estimated based on available information about incidence rates or binary data

$ - HR [95%CI] estimated in two-step MA method approach for 3-arm studies

Supplementary figure 7 Meta-analysis results for all-cause mortality for RIV in VTE


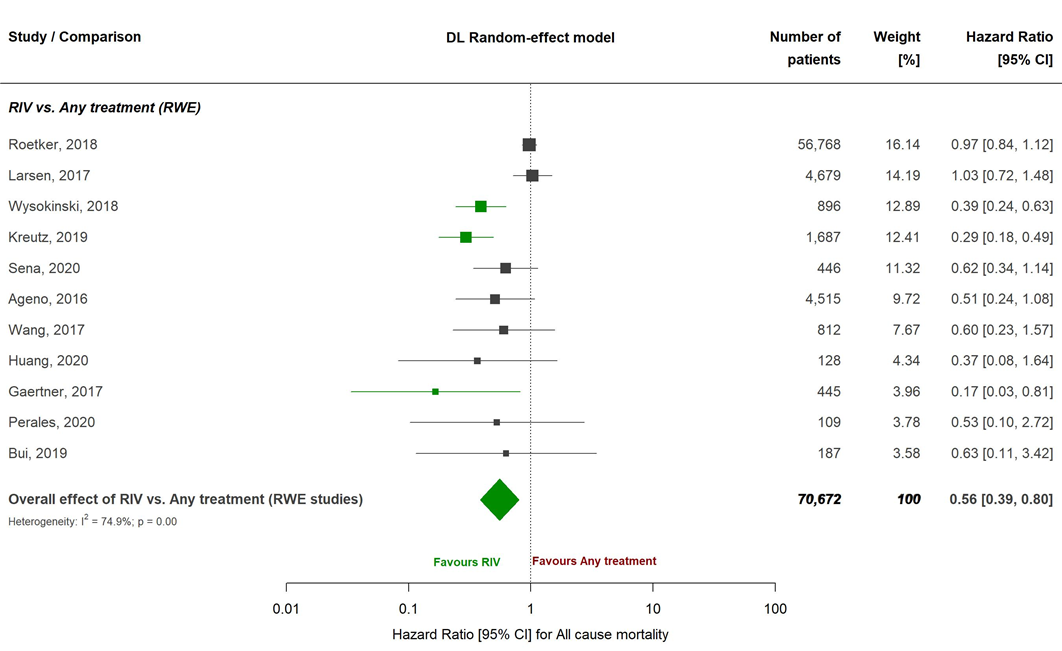


CI = confidence intervals; DL random effect model = DerSimonian-Laird random effects model; I2 = I square statistic; p = p-value; RIV = rivaroxaban; RWE = real world evidence; VTE = Venous thromboembolism; Green marks represent results indicating statistically lower event rate in the RIV group.

Supplementary table 14 Meta-analysis inputs for major bleeding for RIV in VTE

| **Study** | **RIV** | | | **Comparator** | | | | **Database** | **HR [95% CI]** | **Scenario** | | | | | | |
| --- | --- | --- | --- | --- | --- | --- | --- | --- | --- | --- | --- | --- | --- | --- | --- | --- |
|  | **Events (n)** | **N** | **FU (months)** | **Class** | **Events (n)** | **N** | **FU (months)** |  |  | **BC** | **S1** | **S2** | **S3** | **S4** | **S5** | **S6** |
| Kohn, 2019 [31] | 69 | 10,489 | 12 (5.2 ^a^) | VKA± Hep | 248 | 26,364 | 12 (5.2 ^a^) | MarketScan | **0.73 [0.66, 0.93]** | **✓** | **✓** | **✓** |  |  |  |  |
| Coleman, 2018a [16] |  | 10,489 | 6 | VKA± Hep |  | 26,364 | 6 | MarketScan | **0.80 [0.66, 0.98]** |  |  |  |  | **✓** |  |  |
| Coleman, 2018a [16] |  | 10,489 | 3 | VKA± Hep |  | 26,364 | 3 | MarketScan | **0.77 [0.60, 0.98]** |  |  |  |  |  |  |  |
| Coleman, 2018 [13] |  | 4,454 | 6 | VKA± Hep |  | 13,164 | 6 | MarketScan | **0.68 [0.53, 0.88]** | **✓** | **✓** | **✓** |  | **✓** |  |  |
| Coleman, 2018 [13] |  | 4,454 | 12 (4.6 ^a^) | VKA± Hep |  | 13,164 | 12 (4.6^a^) | MarketScan | **0.67 [0.50, 0.90]** |  |  |  |  |  |  |  |
| Coleman, 2018 [13] |  | 4,454 | 3 | VKA± Hep |  | 13,164 | 3 | MarketScan | 0.77 [0.57, 1.06] |  |  |  |  |  |  |  |
| Costa, 2021 [17] |  | 6,755 | 12 | VKA± Hep |  | 6,755 | 12 | Optum | 1.00 [0.73, 1.36] | **✓** | **✓** | **✓** |  |  | **✓** | **✓** |
| Costa, 2021 [17] |  | 6,755 | 6 | VKA± Hep |  | 6,755 | 6 | Optum | 0.90 [0.64, 1.26] |  |  |  |  | **✓** |  |  |
| Costa, 2021 [17] |  | 6,755 | 3 | VKA± Hep |  | 6,755 | 3 | Optum | 0.99 [0.68, 1.44] |  |  |  |  |  |  |  |
| Moustafa, 2018 [41] | 3 | 151 | 5 | VKA± Hep | 26 | 939 | 5 | RIETE | 0.78 [0.49, 1.21] |  |  | **✓** | **✓** |  |  |  |
| Moustafa, 2018 [41] | 19 | 2,348 | 6 | VKA± Hep | 97 | 8,059 | 6 | RIETE | 0.68 [0.41, 1.11]# |  |  | **✓** | **✓** |  |  | **✓** |
| Larsen, 2017 [37] |  | 1,734 | 6 | VKA± Hep |  | 2,945 | 6 | Danish nationwide registries | 1.19 [0.66, 2.13] | **✓** | **✓** | **✓** | **✓** | **✓** |  |  |
| Sena, 2020 [52] | 12 | 134 | 108 | VKA± Hep | 61 | 312 | 108 | Single-centre (Istanbul) | **0.52 [0.28, 0.95]** | **✓** | **✓** | **✓** |  |  | **✓** | **✓** |
| Ageno, 2016 [1] | 19 | 2,505 | ≥12 (5.9 ^a^) | VKA± Hep | 43 | 2,010 | ≥12 (5.9 ^a^) | XALIA trial | 0.77 [0.40, 1.50] | **✓** | **✓** | **✓** | **✓** |  |  |  |
| Kreutz, 2019 [32] | 21 | 1,285 | 7 (5 ^a^) | VKA± Hep | 15 | 402 | 7 (5 ^a^) | XALIA-LEA trial | **0.40 [0.21, 0.78]#** | **✓** |  | **✓** | **✓** |  |  |  |
| Wang, 2017 [59] | 4 | 203 | 3 | VKA± Hep | 16 | 609 | 3 | US Dept. VA | 0.75 [0.25, 2.24]# | **✓** |  | **✓** | **✓** | **✓** |  |  |
| Gaertner, 2017 [25] | 3 | 280 | 6 | VKA± Hep | 3 | 96 | 6 | REMOTEV | 0.34 [0.07, 1.70]# |  |  | **✓** | **✓** |  |  |  |
| Kushnir, 2019 [35] | 2 | 152 | 9 | VKA± Hep | 4 | 167 | 8 | Clinical Looking Glass | 0.54 [0.10, 2.92]# | **✓** |  | **✓** |  |  | **✓** | **✓** |
| Kucher, 2016 [34] |  | 417 | 3 | VKA± Hep |  | 417 | 3 | SWIVTER | 1.00 [0.14, 7.07] | **✓** | **✓** | **✓** | **✓** | **✓** |  |  |
| Bui, 2019 [6] | 1 | 83 | 6 | VKA± Hep | 4 | 104 | 6 | Cho Ray Hospital | 0.31 [0.04, 2.80]# | **✓** |  | **✓** | **✓** | **✓** |  |  |
| Fung, 2019 [24] | 2 | 84 | 12 | VKA± Hep | 1 | 82 | 12 | Single-centre (Hong Kong) | 1.95 [0.18, 21.53]# | **✓** |  | **✓** | **✓** |  | **✓** | **✓** |
| Huang, 2020 [30] | 1 | 86 | 6 | VKA± Hep | 2 | 42 | 6 | Single-centre (Guangzhou) | 0.24 [0.02, 2.69]# | **✓** |  | **✓** | **✓** | **✓** |  |  |
| Sebastian, 2018 [51] | 1 | 73 | 12 (5.9 ^a^) | VKA± Hep | 1 | 38 | 12 (5.9 ^a^) | Swiss Venous Stent registry | 0.52 [0.03, 8.32]# | **✓** |  | **✓** | **✓** |  |  |  |
| Chaudhari, 2019 [8] | 0 | 58 | 6 | VKA± Hep | 4 | 81 | 6 | Single-centre (Bangalore) | 0.13 [0.01, 3.03]# | **✓** |  | **✓** | **✓** | **✓** |  |  |
| Desai, 2016 [22] | 0 | 72 | 6 | VKA± Hep | 1 | 203 | 6 | Winthrop-University Hospital | 0.42 [0.01, 25.57]# | **✓** |  | **✓** |  | **✓** |  |  |
| Moustafa, 2018 [41] | 19 | 2,348 | 6 | Hep | 77 | 3,384 | 6 | RIETE | **0.57 [0.34, 0.92]** |  |  |  |  |  |  |  |
| Moustafa, 2018 [41] | 19 | 2,348 | 6 | Hep | 188 | 11,443 | 6 | RIETE | 0.49 [0.23, 1.06]$ | **✓** |  |  |  |  | **✓** |  |
| Moustafa, 2018 [41] | 3 | 151 | 5 | Hep | 105 | 2,071 | 5 | RIETE | 0.41 [0.15, 1.15] |  |  |  |  |  |  |  |
| Moustafa, 2018 [41] | 3 | 151 | 5 | Hep | 115 | 3,010 | 5 | RIETE | 0.50 [0.15, 1.60]$ | **✓** |  |  |  | **✓** |  |  |
| Gaertner, 2017 [25] | 3 | 280 | 6 | Hep | 6 | 165 | 6 | REMOTEV | 0.29 [0.07, 1.16]$ | **✓** |  |  |  | **✓** |  |  |
| Gaertner, 2017 [25] | 3 | 280 | 6 | Hep | 3 | 69 | 6 | REMOTEV | 0.25 [0.05, 1.22]# |  |  |  |  |  |  |  |

BC = base-case scenario; CI = confidence intervals; FU = follow-up; HR = hazard ratio; Hep = heparin; N = number of patients; RIV = rivaroxaban; S1 = scenario 1 (only HRs reported); S2 = scenario 2 (only VKA±Hep); S3 = scenario 3 (only VKA+Hep at any stage); S4 = scenario 4 (FU ≤6mo); S5 = scenario 5 (FU >6mo); S6 = scenario 6 (only VKA±Hep with FU >6mo); VKA±Hep = vitamin K antagonist ±heparins; VTE = Venous thromboembolism. Green colour indicates result favourable for rivaroxaban. Magenta indicates result favourable for comparator. **Bold** means that the result is significant.

^a^ – mean treatment duration

# - HR [95%CI] estimated based on available information about incidence rates or binary data

$ - HR [95%CI] estimated in two-step MA method approach for 3-arm studies

Supplementary figure 8 Meta-analysis results for major bleeding for RIV in VTE

**
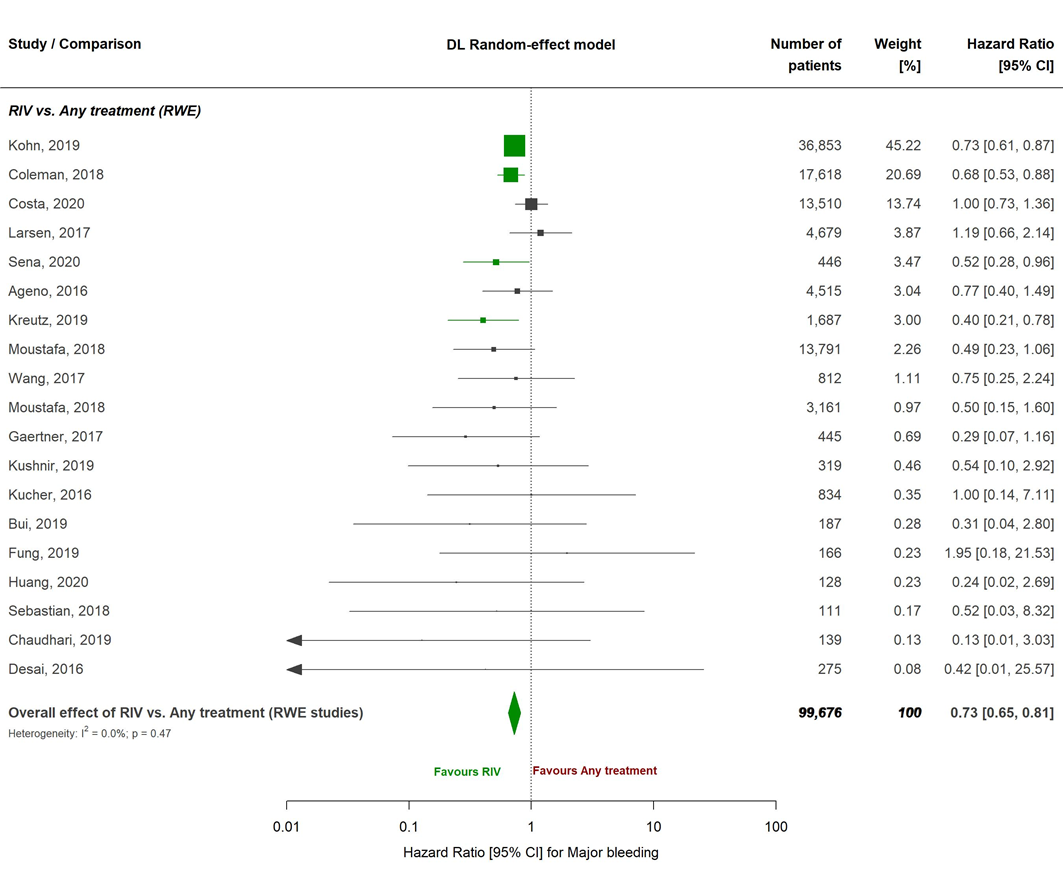
**

CI = confidence intervals; DL random effect model = DerSimonian-Laird random effects model; I2 = I square statistic; p = p-value; RIV = rivaroxaban; RWE = real world evidence; VTE = Venous thromboembolism; Green marks represent results indicating statistically lower event rate in the RIV group.

Supplementary table 15 Meta-analysis inputs for clinically relevant non-major bleeding for RIV in VTE

| **Study** | **RIV** | | | **Comparator** | | | | **Database** | **HR [95% CI]** | **Scenario** | | | | | | |
| --- | --- | --- | --- | --- | --- | --- | --- | --- | --- | --- | --- | --- | --- | --- | --- | --- |
|  | **Events (n)** | **N** | **FU (months)** | **Class** | **Events (n)** | **N** | **FU (months)** |  |  | **BC** | **S1** | **S2** | **S3** | **S4** | **S5** | **S6** |
| Sena, 2020 [52] | 24 | 134 | 108 | VKA± Hep | 51 | 312 | 108 | Single-centre (Istanbul) | 0.77 [0.47, 1.25] | **✓** | **✓** | **✓** |  |  | **✓** | **✓** |
| Bui, 2019 [6] | 9 | 83 | 6 | VKA± Hep | 11 | 104 | 6 | Cho Ray Hospital | 1.03 [0.42, 2.47]# | **✓** |  | **✓** | **✓** | **✓** |  |  |
| Fung, 2019 [24] | 4 | 84 | 12 | VKA± Hep | 10 | 82 | 12 | Single-centre (Hong Kong) | 0.39 [0.12, 1.25]# | **✓** |  | **✓** | **✓** |  | **✓** | **✓** |
| Wysokinski, 2018 [63] | 23 | 293 | N.R. | Hep | 8 | 251 | N.R. | Mayo TCA Registry | **2.31 [1.03, 5.16]** | **✓** | **✓** |  |  |  |  |  |

BC = base-case scenario; CI = confidence intervals; FU = follow-up; HR = hazard ratio; Hep = heparin; N = number of patients; RIV = rivaroxaban; S1 = scenario 1 (only HRs reported); S2 = scenario 2 (only VKA±Hep); S3 = scenario 3 (only VKA+Hep at any stage); S4 = scenario 4 (FU ≤6mo); S5 = scenario 5 (FU >6mo); S6 = scenario 6 (only VKA±Hep with FU >6mo); VKA±Hep = vitamin K antagonist ±heparins; VTE = Venous thromboembolism. Green colour indicates result favourable for rivaroxaban. Magenta indicates result favourable for comparator. **Bold** means that the result is significant.

^a^ – mean treatment duration

# - HR [95%CI] estimated based on available information about incidence rates or binary data

Supplementary figure 9 Meta-analysis results for clinically relevant non-major bleeding for RIV in VTE

**
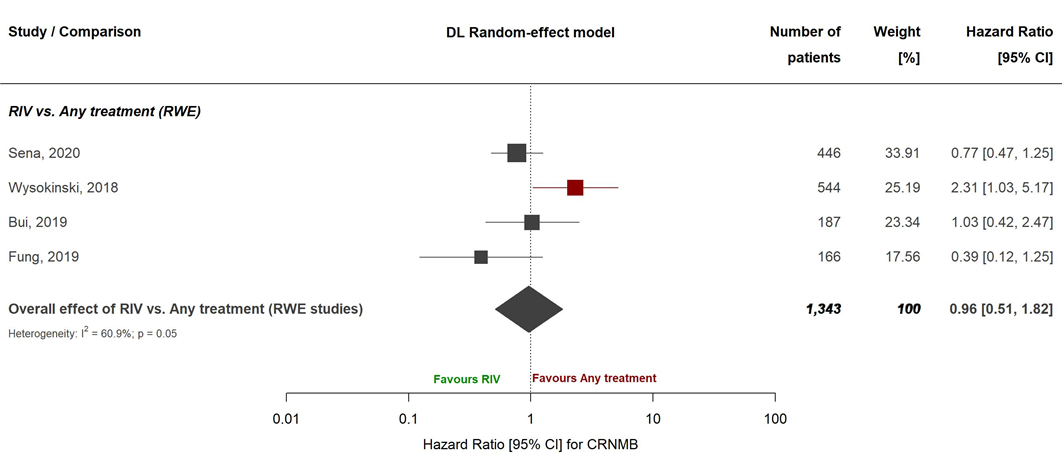
**

CI = confidence intervals; DL random effect model = DerSimonian-Laird random effects model; I2 = I square statistic; p = p-value; RIV = rivaroxaban; RWE = real world evidence; VTE = Venous thromboembolism; statistically significant results indicating inferiority of RIV were presented with green marks.

Supplementary table 16 Meta-analysis inputs for gastrointestinal bleeding for RIV in VTE

| **Study** | **RIV** | | | **Comparator** | | | | **Database** | **HR [95% CI]** | **Scenario** | | | | | | |
| --- | --- | --- | --- | --- | --- | --- | --- | --- | --- | --- | --- | --- | --- | --- | --- | --- |
|  | **Events (n)** | **N** | **FU (months)** | **Class** | **Events (n)** | **N** | **FU (months)** |  |  | **BC** | **S1** | **S2** | **S3** | **S4** | **S5** | **S6** |
| Coleman, 2017 [10] |  | 13,609 | 12 | VKA± Hep | 233 | 32,244 | 12 | MarketScan | **0.72 [0.57, 0.91]** | **✓** | **✓** | **✓** |  |  | **✓** | **✓** |
| Costa, 2020a [18] |  | 2,097 | 12 | VKA± Hep | 47 | 2,842 | 12 | Optum | 0.80 [0.47, 1.37] | **✓** | **✓** | **✓** |  |  | **✓** | **✓** |
| Sindet-Pedersen, 2017 [54] | 59 | 7,572 | 6 | VKA± Hep | 101 | 10,844 | 6 | Danish nationwide registries | 0.84 [0.47, 1.48] | **✓** | **✓** | **✓** |  | **✓** |  |  |
| Costa, 2020a [18] |  | 2,097 | 6 | VKA± Hep | 37 | 2,842 | 6 | Optum | 0.84 [0.47, 1.51] |  |  |  |  | **✓** |  |  |
| Costa, 2020a [18] |  | 2,097 | 3 | VKA± Hep | 24 | 2,842 | 3 | Optum | 1.16 [0.61, 2.21] |  |  |  |  |  |  |  |
| Kreutz, 2019 [32] | 11 | 1,285 | 7 (5 ^a^) | VKA± Hep | 6 | 402 | 7 (5 ^a^) | XALIA-LEA trial | 0.54 [0.20, 1.46]# | **✓** |  | **✓** | **✓** |  |  |  |
| Ageno, 2016 [1] | 3 | 2,619 | 16 (5.9 ^a^) | VKA± Hep | 18 | 2,149 | 16 (5.9 ^a^) | XALIA trial | **0.14 [0.04, 0.46]#** | **✓** |  | **✓** | **✓** |  |  |  |
| Gaertner, 2017 [25] | 1 | 280 | 6 | VKA± Hep | 0 | 96 | 6 | REMOTEV | 2.34 [0.04, 149.07]# |  |  | **✓** | **✓** |  |  |  |
| Gaertner, 2017 [25] | 1 | 280 | 6 | Hep | 0 | 165 | 6 | REMOTEV | 2.59 [0.07, 91.62]$ | **✓** |  |  |  | **✓** |  |  |
| Gaertner, 2017 [25] | 1 | 280 | 6 | Hep | 0 | 69 | 6 | REMOTEV | 2.25 [0.02, 233.40]# |  |  |  |  |  |  |  |

BC = base-case scenario; CI = confidence intervals; FU = follow-up; HR = hazard ratio; Hep = heparin; N = number of patients; RIV = rivaroxaban; S1 = scenario 1 (only HRs reported); S2 = scenario 2 (only VKA±Hep); S3 = scenario 3 (only VKA+Hep at any stage); S4 = scenario 4 (FU ≤6mo); S5 = scenario 5 (FU >6mo); S6 = scenario 6 (only VKA±Hep with FU >6mo); VKA±Hep = vitamin K antagonist ±heparins; VTE = Venous thromboembolism. Green colour indicates result favourable for rivaroxaban. Magenta indicates result favourable for comparator. **Bold** means that the result is significant.

^a^ – mean treatment duration

# - HR [95%CI] estimated based on available information about incidence rates or binary data

$ - HR [95%CI] estimated in two-step MA method approach for 3-arm studies

Supplementary figure 10 Meta-analysis results for gastrointestinal bleeding for RIV in VTE


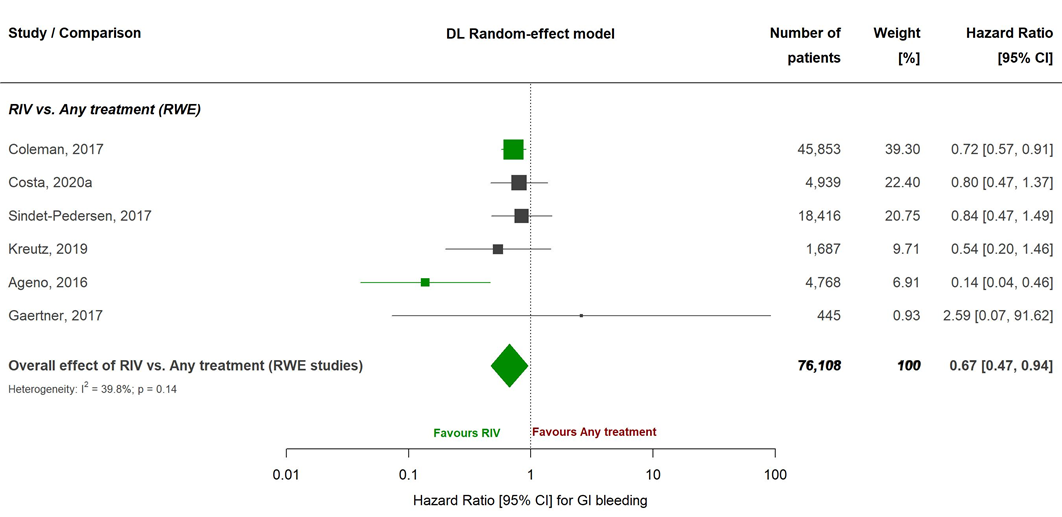


CI = confidence intervals; DL random effect model = DerSimonian-Laird random effects model; I2 = I square statistic; p = p-value; RIV = rivaroxaban; RWE = real world evidence; VTE = Venous thromboembolism; Green marks represent results indicating statistically lower event rate in the RIV group.

Supplementary table 17 Meta-analysis inputs for intracranial haemorrhage for RIV in VTE

| **Study** | **RIV** | | | **Comparator** | | | | **Database** | **HR [95% CI]** | **Scenario** | | | | | | |
| --- | --- | --- | --- | --- | --- | --- | --- | --- | --- | --- | --- | --- | --- | --- | --- | --- |
|  | **Events (n)** | **N** | **FU (months)** | **Class** | **Events (n)** | **N** | **FU (months)** |  |  | **BC** | **S1** | **S2** | **S3** | **S4** | **S5** | **S6** |
| Sindet-Pedersen, 2017 [54] | 22 | 7,572 | 6 | VKA± Hep | 42 | 10,844 | 6 | Danish nationwide registries | 0.97 [0.68, 1.39] | **✓** | **✓** | **✓** |  | **✓** |  |  |
| Coleman, 2017 [10] | 7 | 13,609 | 12 | VKA± Hep | 43 | 32,244 | 12 | MarketScan | **0.40 [0.21, 0.78]** | **✓** | **✓** | **✓** |  |  | **✓** | **✓** |
| Costa, 2021 [17] |  | 6,755 | 12 | VKA± Hep |  | 6,755 | 12 | Optum | 0.44 [0.14, 1.40] | **✓** | **✓** | **✓** |  |  | **✓** | **✓** |
| Costa, 2021 [17] |  | 6,755 | 6 | VKA± Hep |  | 6,755 | 6 | Optum | 0.62 [0.15, 2.58] |  |  |  |  | **✓** |  |  |
| Costa, 2021 [17] |  | 6,755 | 3 | VKA± Hep |  | 6,755 | 3 | Optum | 0.77 [0.17, 3.44] |  |  |  |  |  |  |  |

BC = base-case scenario; CI = confidence intervals; FU = follow-up; HR = hazard ratio; Hep = heparin; N = number of patients; RIV = rivaroxaban; S1 = scenario 1 (only HRs reported); S2 = scenario 2 (only VKA±Hep); S3 = scenario 3 (only VKA+Hep at any stage); S4 = scenario 4 (FU ≤6mo); S5 = scenario 5 (FU >6mo); S6 = scenario 6 (only VKA±Hep with FU >6mo); VKA±Hep = vitamin K antagonist ±heparins; VTE = Venous thromboembolism. Green colour indicates result favourable for rivaroxaban. **Bold** means that the result is significant.

Supplementary figure 11 Meta-analysis results for intracranial haemorrhage for RIV in VTE


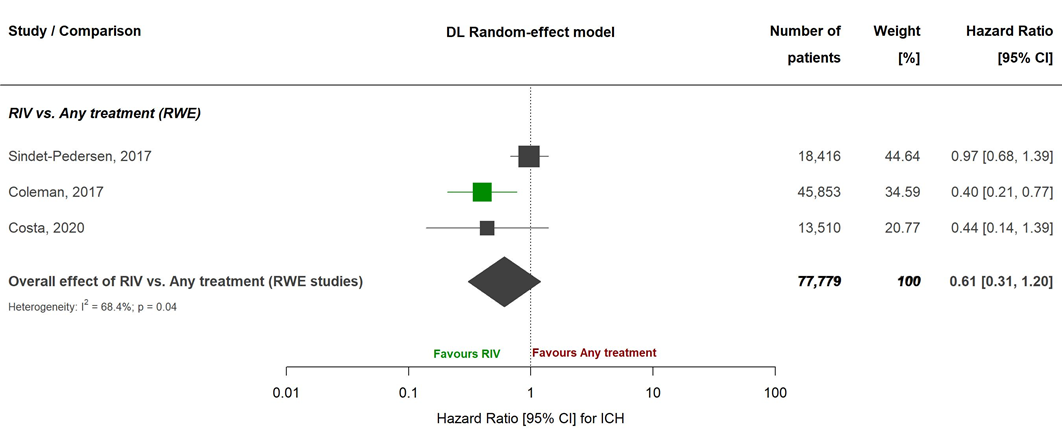


CI = confidence intervals; DL random effect model = DerSimonian-Laird random effects model; I2 = I square statistic; p = p-value; RIV = rivaroxaban; RWE = real world evidence; VTE = Venous thromboembolism; Green marks represent results indicating statistically lower event rate in the RIV group.

Supplementary table 18 Meta-analysis inputs for recurrent venous thromboembolism for API in VTE

| **Study** | **API** | | | **Comparator** | | | | **Database** | **HR [95% CI]** | **Scenario** | | | | | | |
| --- | --- | --- | --- | --- | --- | --- | --- | --- | --- | --- | --- | --- | --- | --- | --- | --- |
|  | **Events (n)** | **N** | **FU (months)** | **Class** | **Events (n)** | **N** | **FU (months)** |  |  | **BC** | **S1** | **S2** | **S3** | **S4** | **S5** | **S6** |
| Weycker, 2018 [62] | 403 | 17,878 | 6 | VKA± Hep | 521 | 17,878 | 6 | MarketScan/PharMetrics/  Optum/Humana | **0.80 [0.70, 0.91]** | **✓** | **✓** | **✓** | **✓** | **✓** |  |  |
| Hlavacek, 2019 [29] |  | 11,363 | 6 | VKA± Hep |  | 11,363 | 6 | Medicare | 1.04 [0.75, 1.43] | **✓** | **✓** | **✓** | **✓** | **✓** |  |  |
| Moustafa, 2018 [41] | 2 | 43 | 5 | VKA± Hep | 23 | 939 | 5 | RIETE | 1.69 [0.36, 7.88]# |  |  | **✓** | **✓** |  |  |  |
| Moustafa, 2018 [41] | 1 | 358 | 6 | VKA± Hep | 97 | 8,059 | 6 | RIETE | 0.27 [0.04, 1.67]# |  |  | **✓** | **✓** |  |  | **✓** |
| Badreldin, 2018 [2] | 1 | 83 | 6 | VKA± Hep | 5 | 180 | 6 | Single-centre (Boston) | 0.43 [0.05, 3.71]# | **✓** |  | **✓** |  | **✓** |  |  |
| Badreldin, 2018 [2] | 1 | 83 | 3 | VKA± Hep | 3 | 180 | 3 | Single-centre (Boston) | 0.72 [0.08, 6.95]# |  |  |  |  |  |  |  |
| Kushnir, 2019 [35] | 1 | 47 | 5 | VKA± Hep | 2 | 167 | 8 | Clinical Looking Glass | 2.78 [0.25, 30.67]# | **✓** |  | **✓** |  |  |  |  |
| Wysokinski, 2018 [63] |  | 281 | N.R. | Hep |  | 251 | N.R. | Mayo TCA Registry | 0.61 [0.23, 1.60] | **✓** | **✓** |  |  |  |  |  |
| Moustafa, 2018 [41] | 2 | 43 | 5 | Hep | 79 | 2,071 | 5 | RIETE | 1.06 [0.24, 4.75]# |  |  |  |  |  |  |  |
| Moustafa, 2018 [41] | 2 | 43 | 5 | Hep | 93 | 3,010 | 5 | RIETE | 1.31 [0.28, 6.22]$ | **✓** |  |  |  | **✓** |  |  |
| Moustafa, 2018 [41] | 1 | 358 | 6 | Hep | 72 | 3,384 | 6 | RIETE | **0.15 [0.02, 0.96]#** |  |  |  |  |  |  |  |
| Moustafa, 2018 [41] | 1 | 358 | 6 | Hep | 182 | 11,443 | 6 | RIETE | 0.21 [0.02, 2.27]$ | **✓** |  |  |  |  | **✓** |  |

API = apixaban; BC = base-case scenario; CI = confidence intervals; FU = follow-up; HR = hazard ratio; Hep = heparin; N = number of patients; S1 = scenario 1 (only HRs reported); S2 = scenario 2 (only VKA±Hep); S3 = scenario 3 (only VKA+Hep at any stage); S4 = scenario 4 (FU ≤6mo); S5 = scenario 5 (FU >6mo); S6 = scenario 6 (only VKA±Hep with FU >6mo); VKA±Hep = vitamin K antagonist ±heparins; VTE = Venous thromboembolism.

Green colour indicates result favourable for apixaban. Magenta indicates result favourable for comparator. **Bold** means that the result is significant.

# - HR [95%CI] estimated based on available information about incidence rates or binary data

$ - HR [95%CI] estimated in two-step MA method approach for 3-arm studies

Supplementary figure 12 Meta-analysis results for recurrent VTE for API in VTE


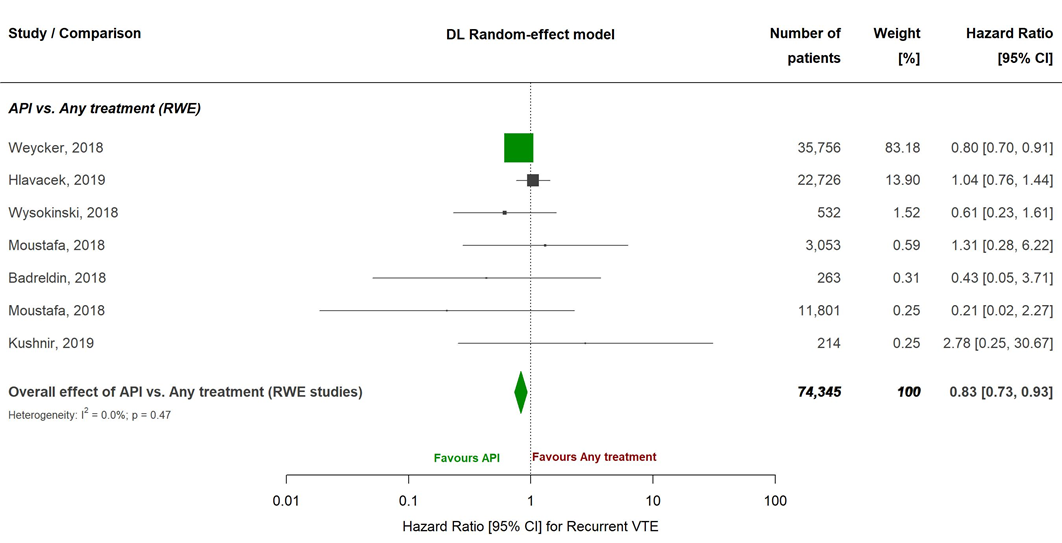


CI = confidence intervals; DL random effect model = DerSimonian-Laird random effects model; I2 = I square statistic; p = p-value; RIV = rivaroxaban; RWE = real world evidence; VTE = Venous thromboembolism; Green marks represent results indicating statistically lower event rate in the API group.

Supplementary table 19 Meta-analysis inputs for recurrent pulmonary embolism for API in VTE

| **Study** | **API** | | | **Comparator** | | | | **Database** | **HR [95% CI]** | **Scenario** | | | | | | |
| --- | --- | --- | --- | --- | --- | --- | --- | --- | --- | --- | --- | --- | --- | --- | --- | --- |
|  | **Events (n)** | **N** | **FU (months)** | **Class** | **Events (n)** | **N** | **FU (months)** |  |  | **BC** | **S1** | **S2** | **S3** | **S4** | **S5** | **S6** |
| Dawwas, 2020 [20] | 129 | 8,094 | 12 | VKA± Hep | 1,026 | 28,813 | 12 | MarketScan | **0.54 [0.45, 0.65]** | **✓** | **✓** | **✓** |  |  | **✓** | **✓** |

API = apixaban; BC = base-case scenario; CI = confidence intervals; FU = follow-up; HR = hazard ratio; N = number of patients; S1 = scenario 1 (only HRs reported); S2 = scenario 2 (only VKA±Hep); S3 = scenario 3 (only VKA+Hep at any stage); S4 = scenario 4 (FU ≤6mo); S5 = scenario 5 (FU >6mo); S6 = scenario 6 (only VKA±Hep with FU >6mo); VKA±Hep = vitamin K antagonist ±heparins; VTE = Venous thromboembolism. Green colour indicates result favourable for apixaban. **Bold** means that the result is significant.

Supplementary table 20 Meta-analysis inputs for recurrent deep vein thrombosis for API in VTE

| **Study** | **API** | | | **Comparator** | | | | **Database** | **HR [95% CI]** | **Scenario** | | | | | | |
| --- | --- | --- | --- | --- | --- | --- | --- | --- | --- | --- | --- | --- | --- | --- | --- | --- |
|  | **Events (n)** | **N** | **FU (months)** | **Class** | **Events (n)** | **N** | **FU (months)** |  |  | **BC** | **S1** | **S2** | **S3** | **S4** | **S5** | **S6** |
| Dawwas, 2020 [20] | 116 | 8,094 | 12 | VKA± Hep | 638 | 28,813 | 12 | MarketScan | **0.79 [0.65, 0.97]** | **✓** | **✓** | **✓** |  |  | **✓** | **✓** |

API = apixaban; BC = base-case scenario; CI = confidence intervals; FU = follow-up; HR = hazard ratio; N = number of patients; S1 = scenario 1 (only HRs reported); S2 = scenario 2 (only VKA±Hep); S3 = scenario 3 (only VKA+Hep at any stage); S4 = scenario 4 (FU ≤6mo); S5 = scenario 5 (FU >6mo); S6 = scenario 6 (only VKA±Hep with FU >6mo); VKA±Hep = vitamin K antagonist ±heparins; VTE = Venous thromboembolism. Green colour indicates result favourable for apixaban. **Bold** means that the result is significant.

Supplementary table 21 Meta-analysis inputs for all-cause mortality for API in VTE

| **Study** | **API** | | | **Comparator** | | | | **Database** | **HR [95% CI]** | **Scenario** | | | | | | |
| --- | --- | --- | --- | --- | --- | --- | --- | --- | --- | --- | --- | --- | --- | --- | --- | --- |
|  | **Events (n)** | **N** | **FU (months)** | **Class** | **Events (n)** | **N** | **FU (months)** |  |  | **BC** | **S1** | **S2** | **S3** | **S4** | **S5** | **S6** |
| Roetker, 2018 [50] | 155 | 5,663 | 6 | VKA± Hep | 545 | 16,269 | 6 | OptumLabs | 0.91 [0.72, 1.15] | **✓** | **✓** | **✓** |  | **✓** |  |  |
| Bouget, 2020 [3] |  | 3,460 | 36 (5.4 ^a^) | VKA± Hep |  | 20,205 | 36 (7.7 ^a^) | SNIIRAM | 1.15 [0.37, 3.57] | **✓** | **✓** | **✓** |  |  |  |  |
| Wysokinski, 2018 [63] |  | 281 | N.R. | Hep |  | 251 | N.R. | Mayo TCA Registry | **0.32 [0.19, 0.56]** | **✓** | **✓** |  |  |  |  |  |

API = apixaban; BC = base-case scenario; CI = confidence intervals; FU = follow-up; HR = hazard ratio; Hep = heparin; N = number of patients; S1 = scenario 1 (only HRs reported); S2 = scenario 2 (only VKA±Hep); S3 = scenario 3 (only VKA+Hep at any stage); S4 = scenario 4 (FU ≤6mo); S5 = scenario 5 (FU >6mo); S6 = scenario 6 (only VKA±Hep with FU >6mo); VKA±Hep = vitamin K antagonist ±heparins; VTE = Venous thromboembolism. Green colour indicates result favourable for apixaban. Magenta indicates result favourable for comparator. **Bold** means that the result is significant.

^a^ – median treatment duration

Supplementary figure 13 Meta-analysis results for all-cause mortality for API in VTE


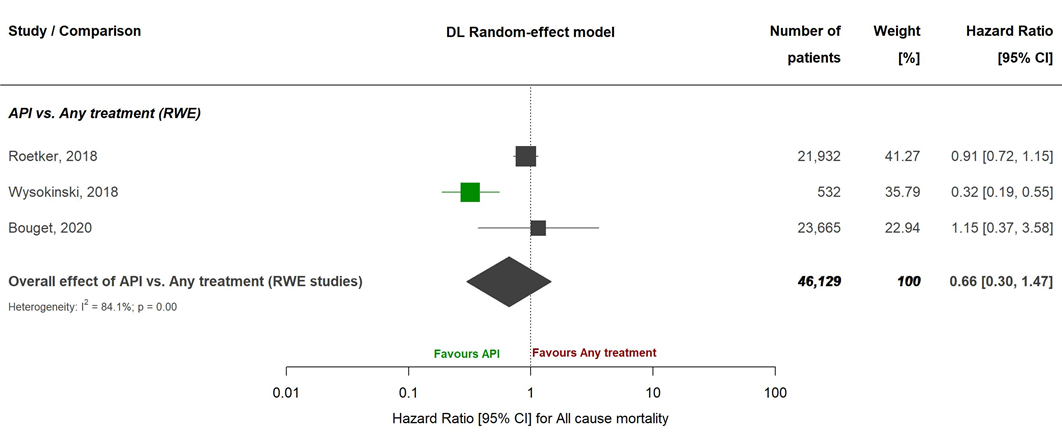


API = apixaban; CI = confidence intervals; DL random effect model = DerSimonian-Laird random effects model; I2 = I square statistic; p = p-value; RWE = real world evidence; VTE = Venous thromboembolism; Green marks represent results indicating statistically lower event rate in the API group.

Supplementary table 22 Meta-analysis inputs for major bleeding for API in VTE

| **Study** | **API** | | | **Comparator** | | | | **Database** | **HR [95% CI]** | **Scenario** | | | | | | |
| --- | --- | --- | --- | --- | --- | --- | --- | --- | --- | --- | --- | --- | --- | --- | --- | --- |
|  | **Events (n)** | **N** | **FU (months)** | **Class** | **Events (n)** | **N** | **FU (months)** |  |  | **BC** | **S1** | **S2** | **S3** | **S4** | **S5** | **S6** |
| Weycker, 2018 [62] |  | 17,878 | 6 | VKA± Hep | 412 | 17,878 | 6 | MarketScan/PharMetrics/  Optum/Humana | **0.75 [0.64, 0.87]** | **✓** | **✓** | **✓** | **✓** | **✓** |  |  |
| Hlavacek, 2019 [29] |  | 11,363 | 6 | VKA± Hep |  | 11,363 | 6 | Medicare | **0.76 [0.64, 0.91]** | **✓** | **✓** | **✓** | **✓** | **✓** |  |  |
| Moustafa, 2018 [41] | 6 | 358 | 6 | VKA± Hep | 97 | 8,059 | 6 | RIETE | 1.39 [0.61, 3.17]# |  |  | **✓** | **✓** |  |  | **✓** |
| Moustafa, 2018 [41] | 1 | 43 | 5 | VKA± Hep | 26 | 939 | 5 | RIETE | 0.70 [0.08, 6.16]# |  |  | **✓** | **✓** |  |  |  |
| Kushnir, 2019 [35] | 1 | 47 | 5 | VKA± Hep | 4 | 167 | 8 | Clinical Looking Glass | 1.39 [0.16, 12.44]# | **✓** |  | **✓** |  |  |  |  |
| Moustafa, 2018 [41] | 6 | 358 | 6 | Hep | 77 | 3,384 | 6 | RIETE | 0.73 [0.32, 1.68]# |  |  |  |  |  |  |  |
| Wysokinski, 2018 [63] |  | 281 | N.R. | Hep |  | 251 | N.R. | Mayo TCA Registry | 0.88 [0.37, 2.12] | **✓** | **✓** |  |  |  |  |  |
| Moustafa, 2018 [41] | 6 | 358 | 6 | Hep | 188 | 11,443 | 6 | RIETE | 1.01 [0.34, 3.02]$ | **✓** |  |  |  |  | **✓** |  |
| Moustafa, 2018 [41] | 1 | 43 | 5 | Hep | 105 | 2,071 | 5 | RIETE | 0.39 [0.05, 3.31]# |  |  |  |  |  |  |  |
| Moustafa, 2018 [41] | 1 | 43 | 5 | Hep | 115 | 3,010 | 5 | RIETE | 0.51 [0.05, 5.46]$ | **✓** |  |  |  | **✓** |  |  |

API = apixaban; BC = base-case scenario; CI = confidence intervals; FU = follow-up; HR = hazard ratio; Hep = heparin; N = number of patients; S1 = scenario 1 (only HRs reported); S2 = scenario 2 (only VKA±Hep); S3 = scenario 3 (only VKA+Hep at any stage); S4 = scenario 4 (FU ≤6mo); S5 = scenario 5 (FU >6mo); S6 = scenario 6 (only VKA±Hep with FU >6mo); VKA±Hep = vitamin K antagonist ±heparins; VTE = Venous thromboembolism. Green colour indicates result favourable for apixaban. Magenta indicates result favourable for comparator. **Bold** means that the result is significant.

# - HR [95%CI] estimated based on available information about incidence rates or binary data

$ - HR [95%CI] estimated in two-step MA method approach for 3-arm studies

Supplementary figure 14 Meta-analysis results for major bleeding for API in VTE

**
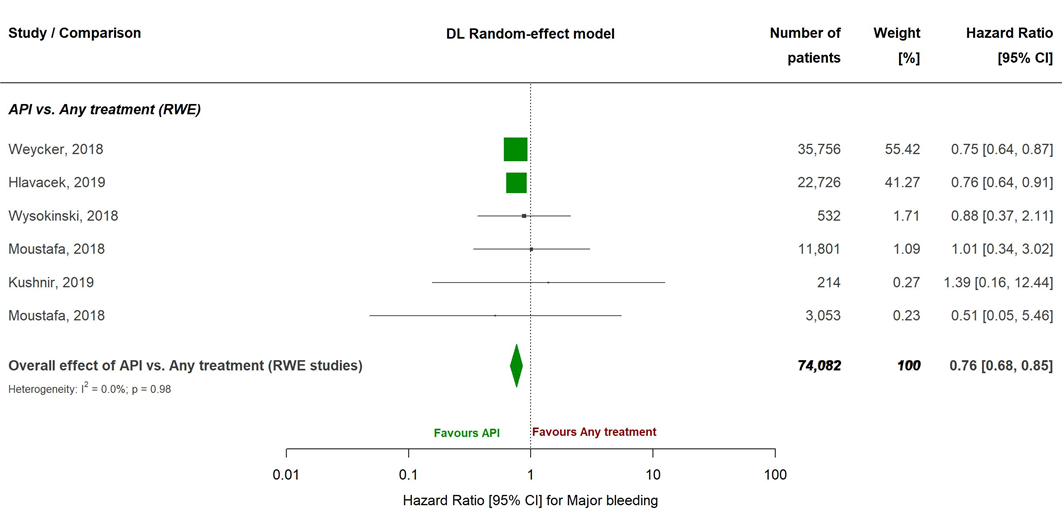
**

API = apixaban; CI = confidence intervals; DL random effect model = DerSimonian-Laird random effects model; I2 = I square statistic; p = p-value; RWE = real world evidence; VTE = Venous thromboembolism; Green marks represent results indicating statistically lower event rate in the API group.

Supplementary table 23 Meta-analysis inputs for clinically relevant non-major bleeding for API in VTE

| **Study** | **API** | | | **Comparator** | | | | **Database** | **HR [95% CI]** | **Scenario** | | | | | | |
| --- | --- | --- | --- | --- | --- | --- | --- | --- | --- | --- | --- | --- | --- | --- | --- | --- |
|  | **Events (n)** | **N** | **FU (months)** | **Class** | **Events (n)** | **N** | **FU (months)** |  |  | **BC** | **S1** | **S2** | **S3** | **S4** | **S5** | **S6** |
| Weycker, 2018 [62] |  | 17,878 | 6 | VKA± Hep | 1,688 | 17,878 | 6 | MarketScan/PharMetrics/  Optum/Humana | **0.77 [0.71, 0.83]** | **✓** | **✓** | **✓** | **✓** | **✓** |  |  |
| Hlavacek, 2019 [29] |  | 11,363 | 6 | VKA± Hep |  | 11,363 | 6 | Medicare | **0.76 [0.70, 0.84]** | **✓** | **✓** | **✓** | **✓** | **✓** |  |  |
| Wysokinski, 2018 [63] | 3 | 281 | N.R. | Hep | 8 | 251 | N.R. | Mayo TCA Registry | 0.34 [0.09, 1.29] | **✓** | **✓** |  |  |  |  |  |

API = apixaban; BC = base-case scenario; CI = confidence intervals; FU = follow-up; HR = hazard ratio; Hep = heparin; N = number of patients; S1 = scenario 1 (only HRs reported); S2 = scenario 2 (only VKA±Hep); S3 = scenario 3 (only VKA+Hep at any stage); S4 = scenario 4 (FU ≤6mo); S5 = scenario 5 (FU >6mo); S6 = scenario 6 (only VKA±Hep with FU >6mo); VKA±Hep = vitamin K antagonist ±heparins; VTE = Venous thromboembolism. Green colour indicates result favourable for apixaban. **Bold** means that the result is significant.

Supplementary figure 15 Meta-analysis results for clinically relevant non-major bleeding for API in VTE

**
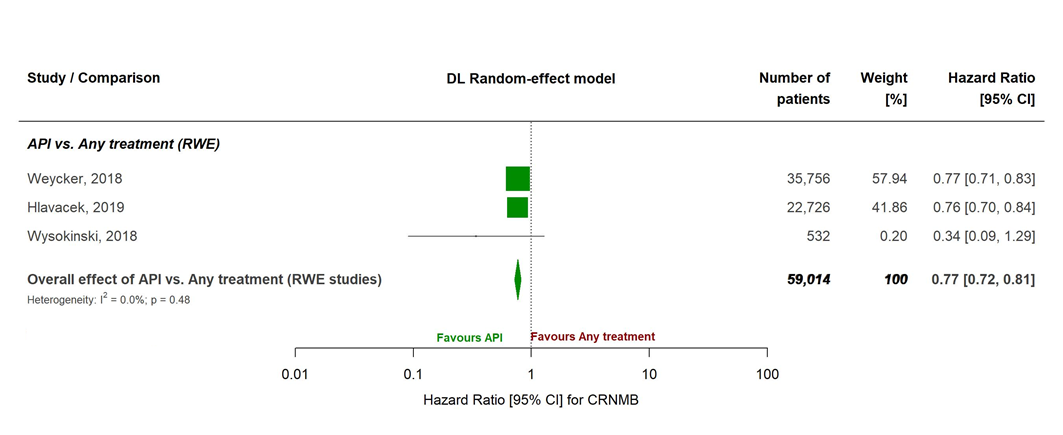
**

API = apixaban; CI = confidence intervals; DL random effect model = DerSimonian-Laird random effects model; I2 = I square statistic; p = p-value; RWE = real world evidence; VTE = Venous thromboembolism; Green marks represent results indicating statistically lower event rate in the API group.

Supplementary table 24 Meta-analysis inputs for gastrointestinal bleeding for API in VTE

| **Study** | **API** | | | **Comparator** | | | | **Database** | **HR [95% CI]** | **Scenario** | | | | | | |
| --- | --- | --- | --- | --- | --- | --- | --- | --- | --- | --- | --- | --- | --- | --- | --- | --- |
|  | **Events (n)** | **N** | **FU (months)** | **Class** | **Events (n)** | **N** | **FU (months)** |  |  | **BC** | **S1** | **S2** | **S3** | **S4** | **S5** | **S6** |
| Weycker, 2018 [62] |  | 17,878 | 5 | VKA± Hep | 160 | 17,878 | 5 | MarketScan/PharMetrics/  Optum/Humana | **0.72 [0.57, 0.92]** | **✓** | **✓** | **✓** | **✓** | **✓** |  |  |
| Hlavacek, 2019 [29] |  | 11,363 | 6 | VKA± Hep |  | 11,363 | 6 | Medicare | 0.97 [0.75, 1.25] | **✓** | **✓** | **✓** | **✓** | **✓** |  |  |

API = apixaban; BC = base-case scenario; CI = confidence intervals; FU = follow-up; HR = hazard ratio; N = number of patients; S1 = scenario 1 (only HRs reported); S2 = scenario 2 (only VKA±Hep); S3 = scenario 3 (only VKA+Hep at any stage); S4 = scenario 4 (FU ≤6mo); S5 = scenario 5 (FU >6mo); S6 = scenario 6 (only VKA±Hep with FU >6mo); VKA±Hep = vitamin K antagonist ±heparins; VTE = Venous thromboembolism. Green colour indicates result favourable for apixaban. **Bold** means that the result is significant.

Supplementary figure 16 Meta-analysis results for gastrointestinal bleeding for API in VTE


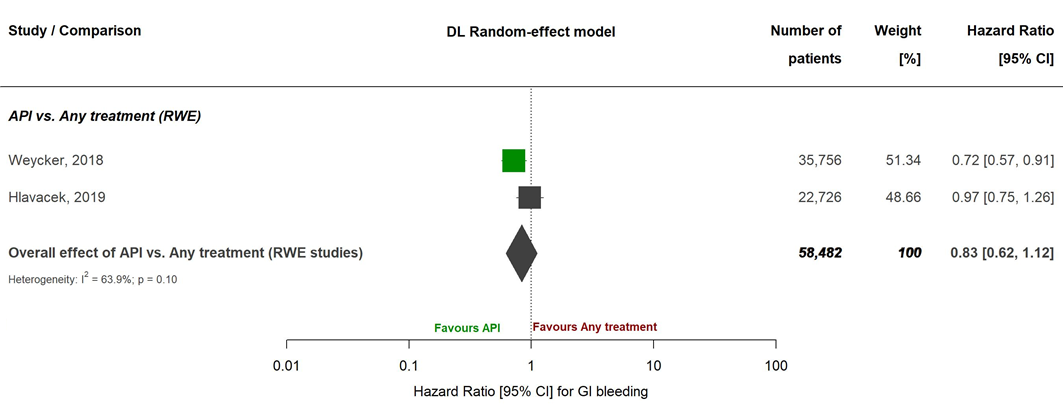


API = apixaban; CI = confidence intervals; DL random effect model = DerSimonian-Laird random effects model; I2 = I square statistic; p = p-value; RWE = real world evidence; VTE = Venous thromboembolism; Green marks represent results indicating statistically lower event rate in the API group.

Supplementary table 25 Meta-analysis inputs for intracranial haemorrhage for API in VTE

| **Study** | **API** | | | **Comparator** | | | | **Database** | **HR [95% CI]** | **Scenario** | | | | | | |
| --- | --- | --- | --- | --- | --- | --- | --- | --- | --- | --- | --- | --- | --- | --- | --- | --- |
|  | **Events (n)** | **N** | **FU (months)** | **Class** | **Events (n)** | **N** | **FU (months)** |  |  | **BC** | **S1** | **S2** | **S3** | **S4** | **S5** | **S6** |
| Hlavacek, 2019 [29] |  | 11,363 | 6 | VKA± Hep |  | 11,363 | 6 | Medicare | 0.62 [0.38, 1.01] | **✓** | **✓** | **✓** | **✓** | **✓** |  |  |
| Weycker, 2018 [62] |  | 17,878 | 6 | VKA± Hep | 26 | 17,878 | 6 | MarketScan/PharMetrics/  Optum/Humana | 0.97 [0.56, 1.69] | **✓** | **✓** | **✓** | **✓** | **✓** |  |  |

API = apixaban; BC = base-case scenario; CI = confidence intervals; FU = follow-up; HR = hazard ratio; N = number of patients; S1 = scenario 1 (only HRs reported); S2 = scenario 2 (only VKA±Hep); S3 = scenario 3 (only VKA+Hep at any stage); S4 = scenario 4 (FU ≤6mo); S5 = scenario 5 (FU >6mo); S6 = scenario 6 (only VKA±Hep with FU >6mo); VKA±Hep = vitamin K antagonist ±heparins; VTE = Venous thromboembolism. Green colour indicates result favourable for apixaban.

Supplementary figure 17 Meta-analysis results for intracranial haemorrhage for API in VTE


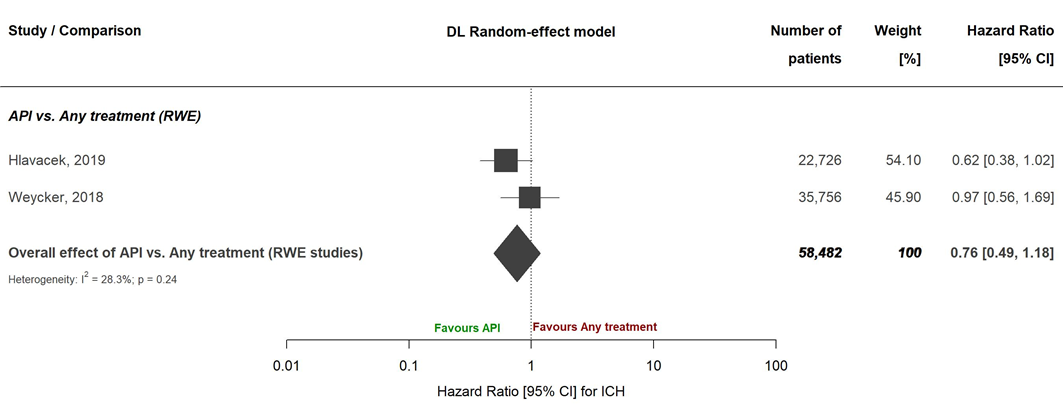


API = apixaban; CI = confidence intervals; DL random effect model = DerSimonian-Laird random effects model; I2 = I square statistic; p = p-value; RWE = real world evidence; VTE = Venous thromboembolism.

Supplementary figure 18 Funnel plot for recurrent venous thromboembolism for RIV in VTE, base scenario


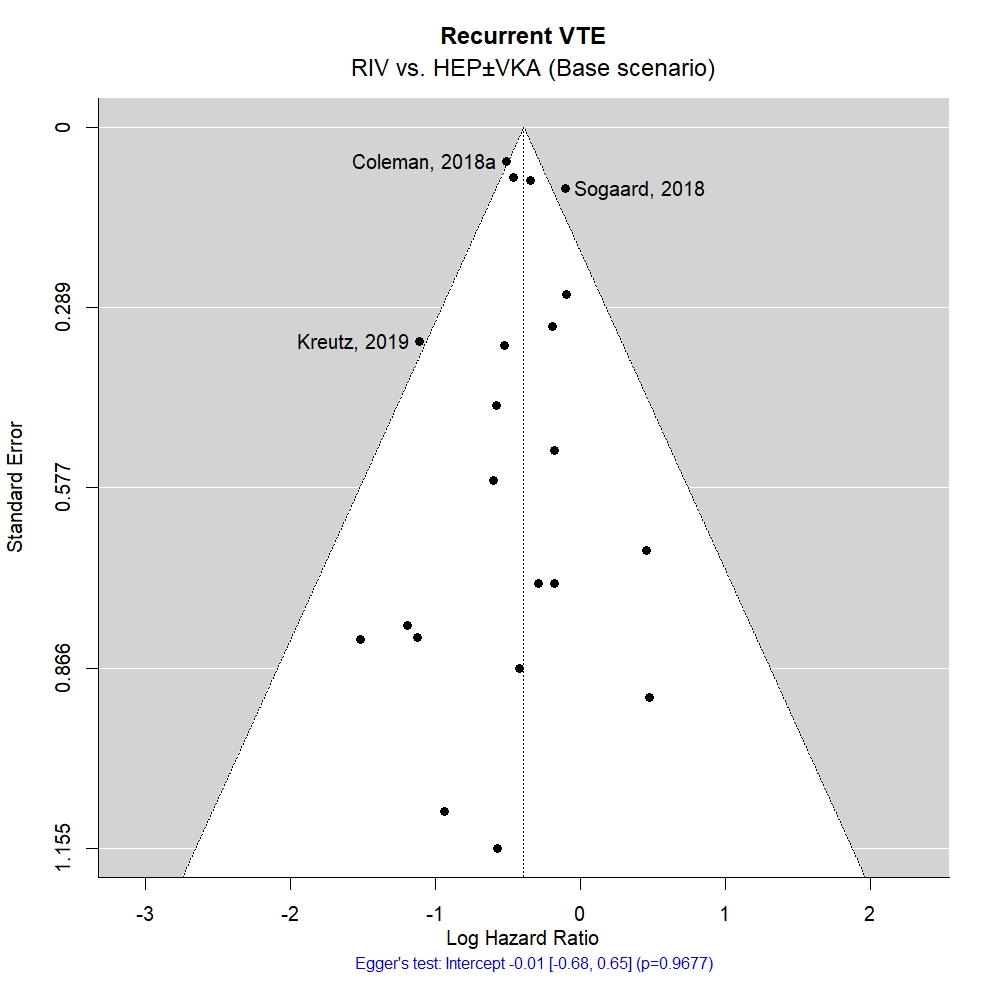


Supplementary figure 19 Funnel plot for recurrent venous thromboembolism for RIV in VTE, Scenario 2: only vs VKA±Hep


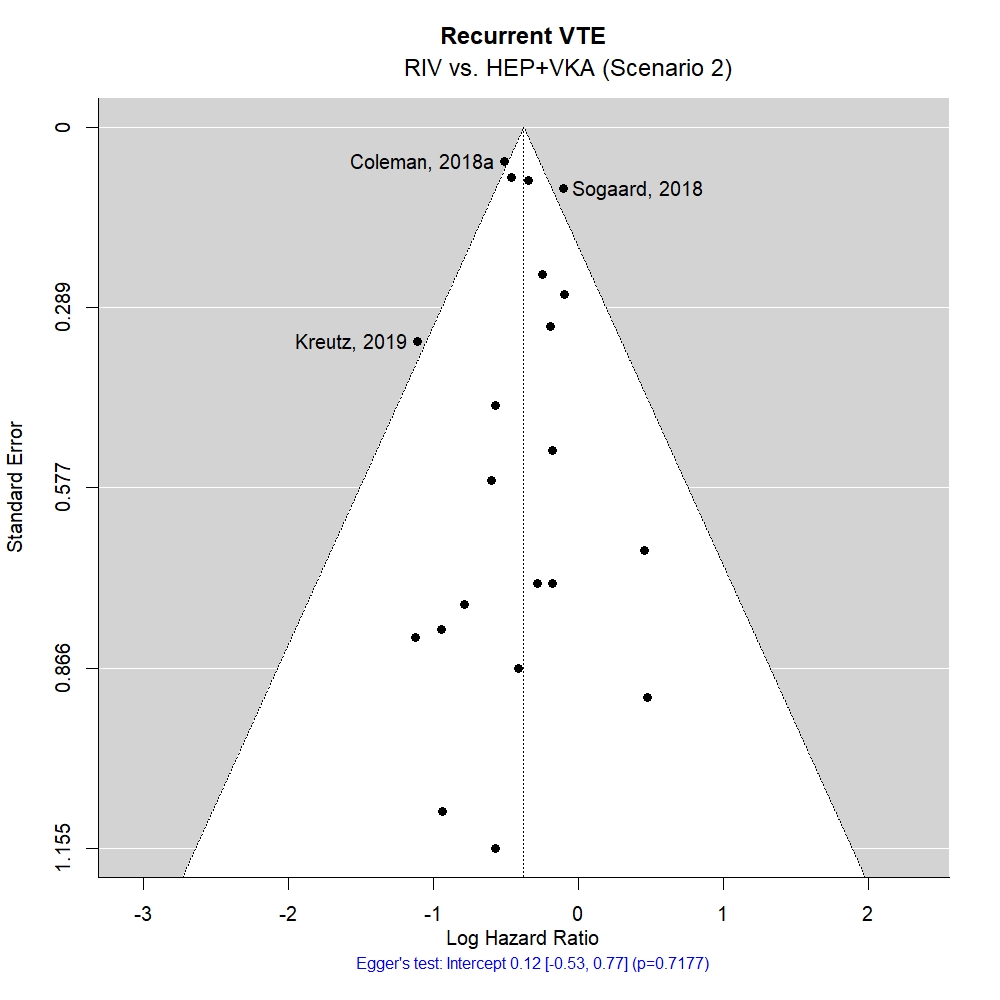


Supplementary figure 20 Funnel plot for recurrent venous thromboembolism for RIV in VTE, Scenario 3: only vs VKA+Hep


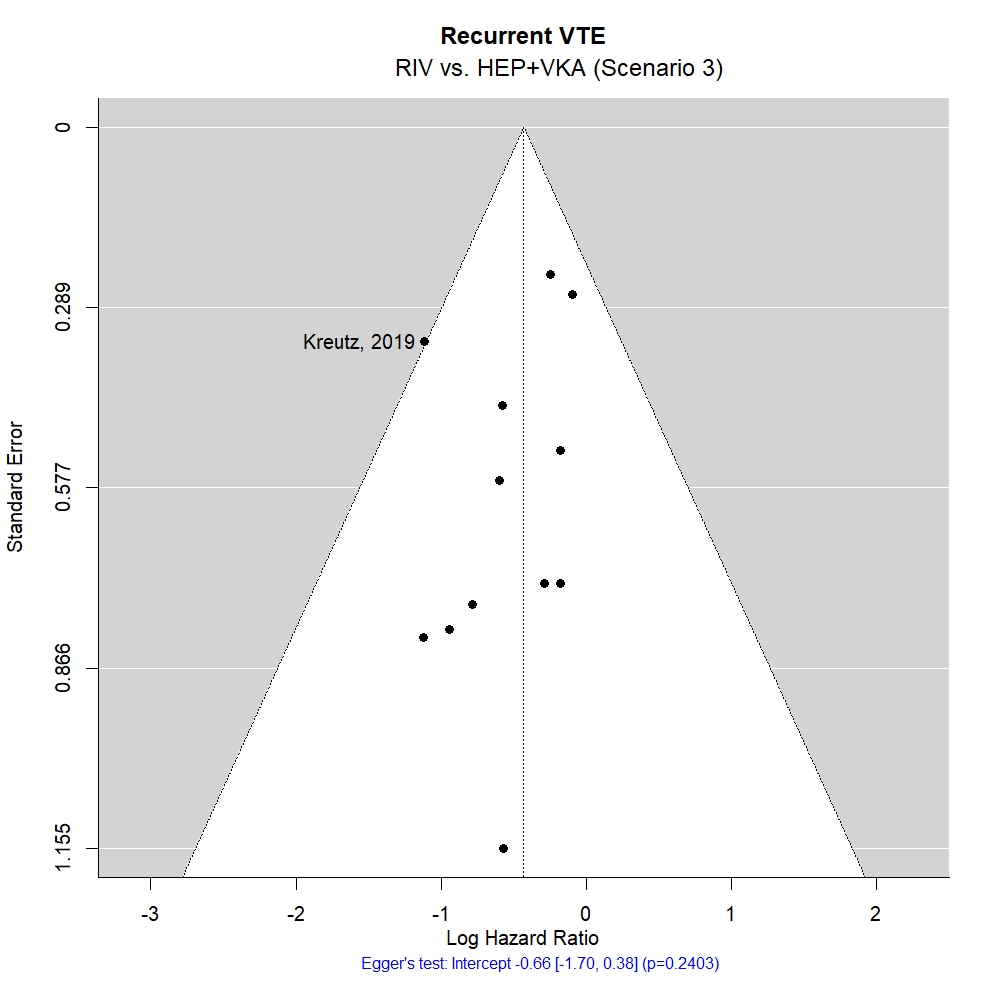


Supplementary figure 21 Funnel plot for recurrent venous thromboembolism for RIV in VTE, Scenario 4: ≤6 months treatment


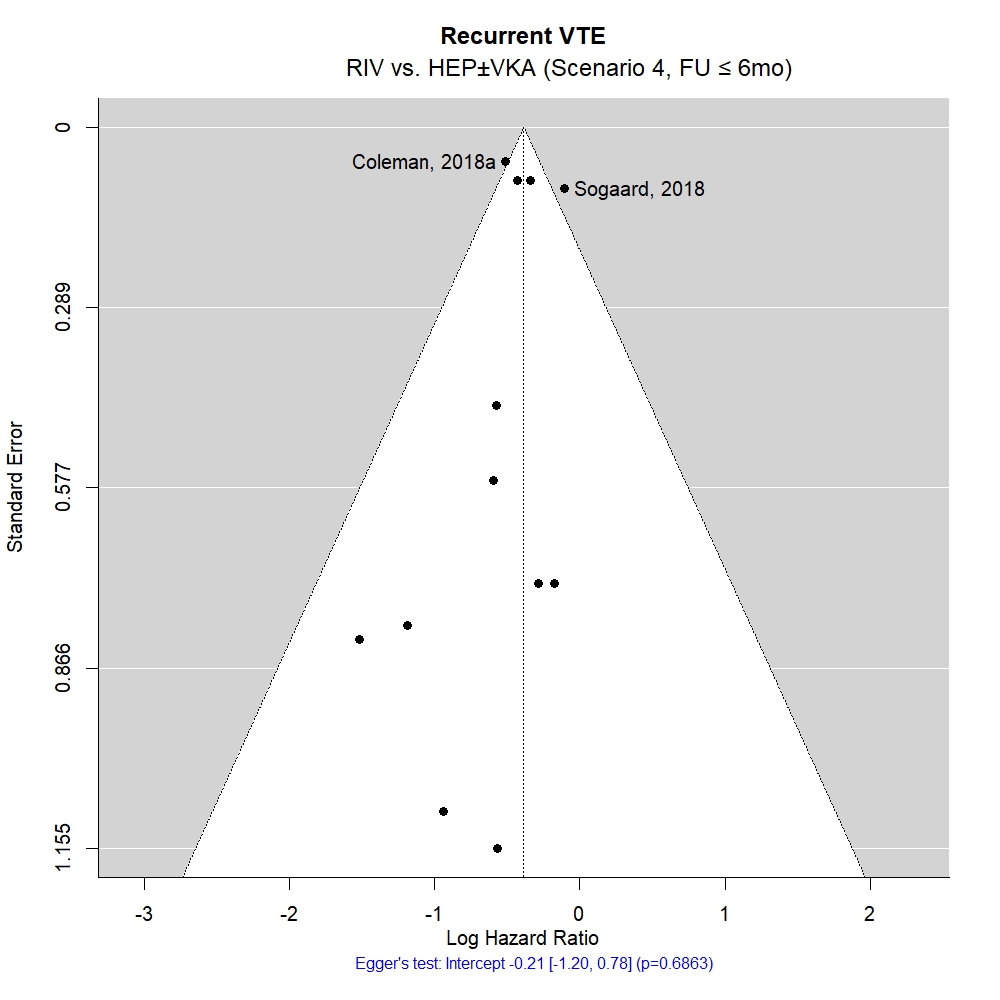


Supplementary figure 22 Funnel plot for all-cause mortality for RIV in VTE, base scenario


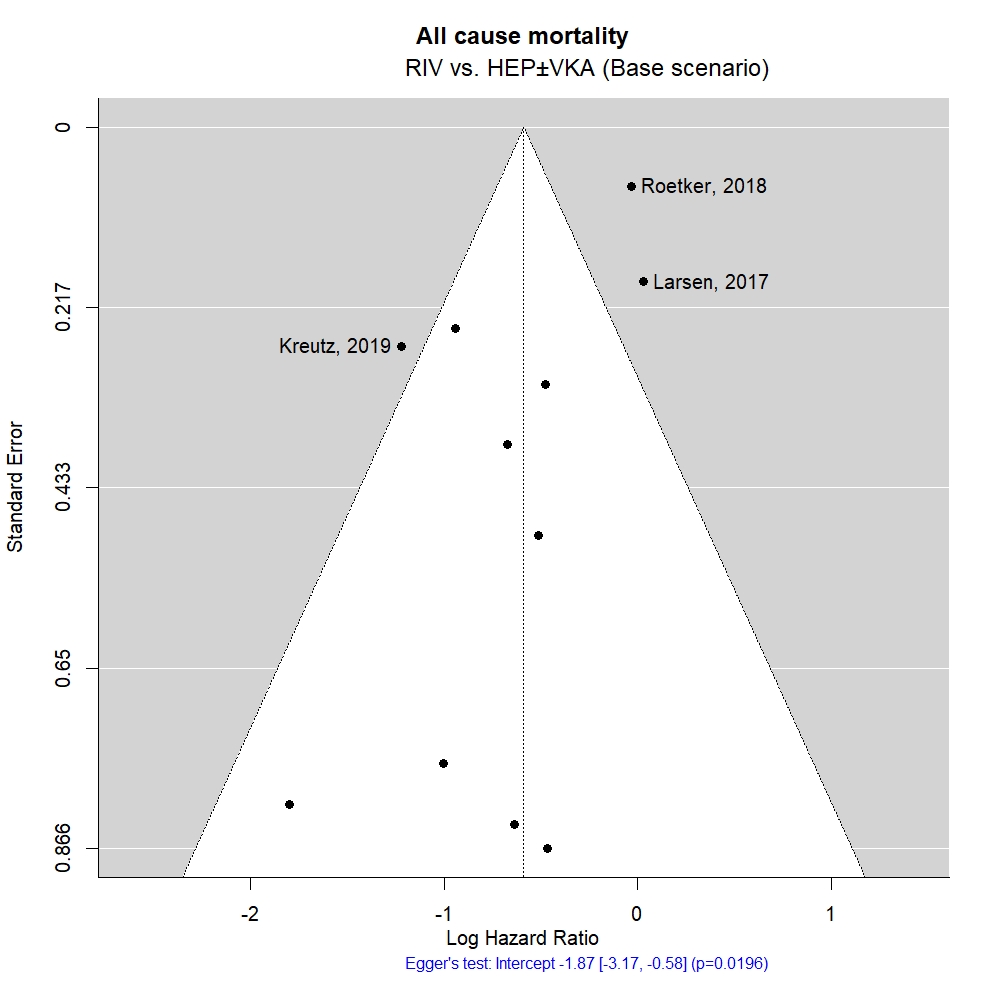


Supplementary figure 23 Funnel plot for all-cause mortality for RIV in VTE, base scenario (without outlier: Roetker, 2018)


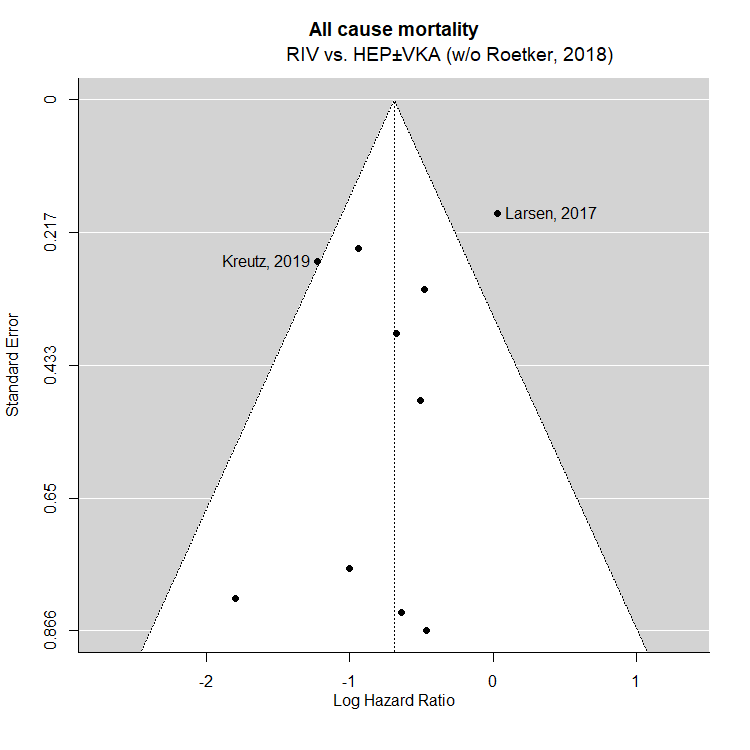


Egger's test: Intercept -1.13 [-3.14, 0.87] (p=0.3004)

Hazard Ratio [95% CI] w/o outlier (Roetker, 2018): 0.50 [0.34, 0.73]

Heterogeneity: I², = 58.7%; p = 0.01

Following outlier removal (Roetker, 2018), Egger’s test was repeated and new overall hazard ratio was estimated.

Supplementary figure 24 Funnel plot for all-cause mortality for RIV in VTE, base scenario (trim and fill)


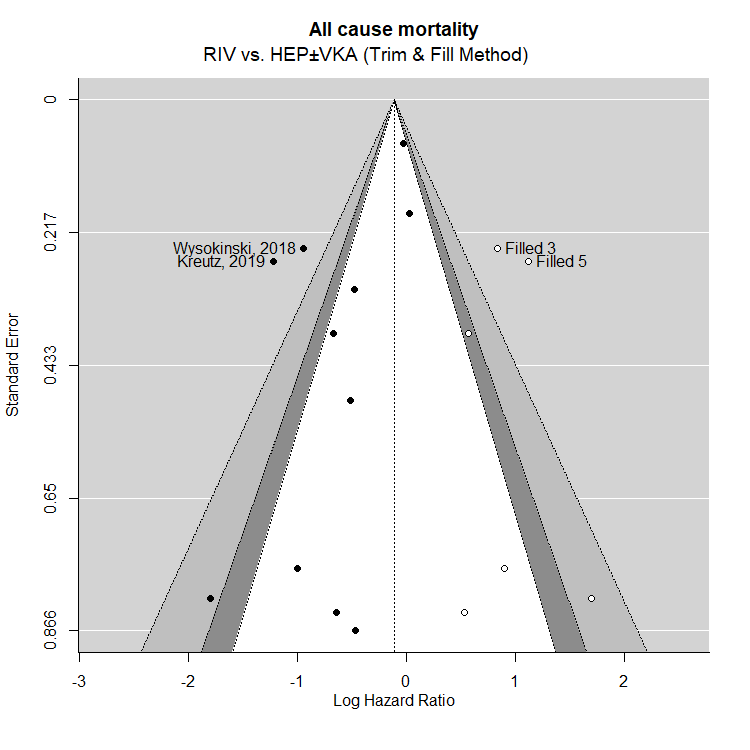


Hazard Ratio [95% CI] (Trim & Fill Method): 0.89 [0.62, 1.28]

Following imputation of ‘missing’ studies using trim & fill method added until reaching the symmetry of funnel plot, the new overall hazard ratio was estimated.

Supplementary figure 25 Funnel plot for all-cause mortality for RIV in VTE, Scenario 2: only vs VKA±Hep


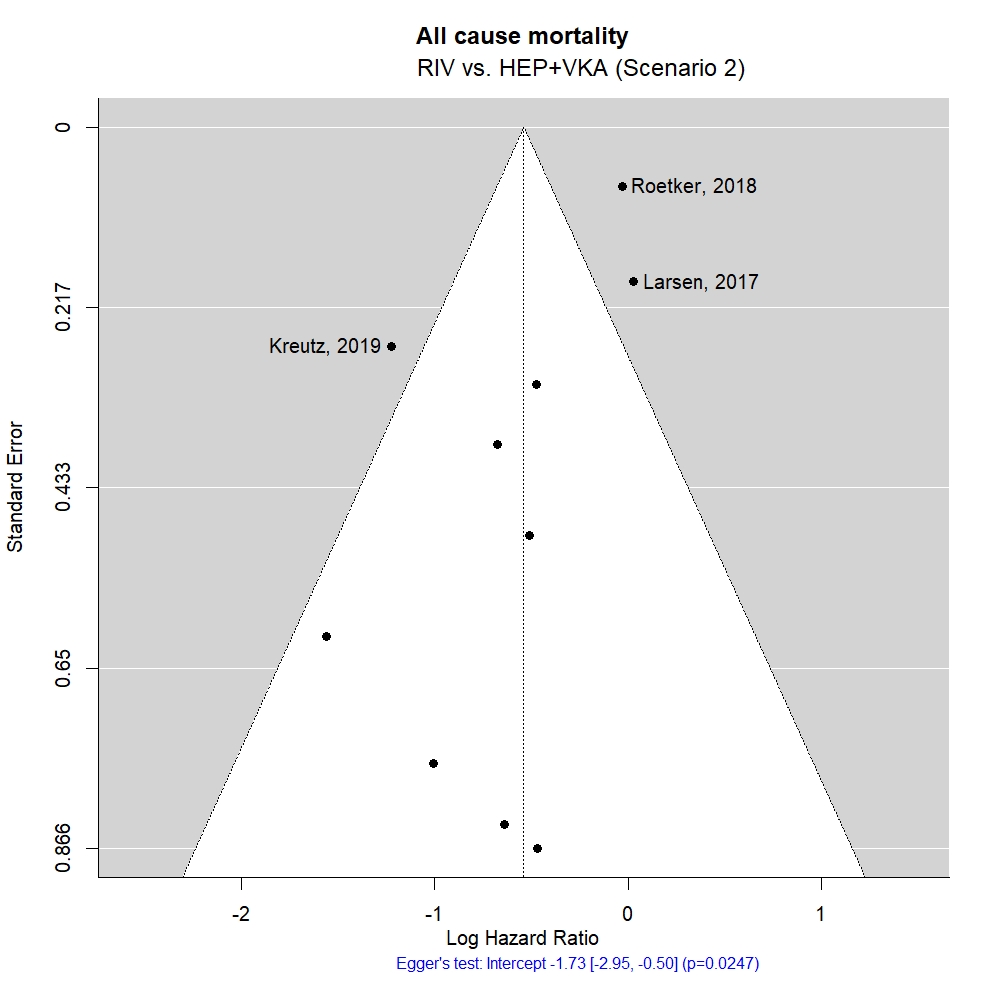


Supplementary figure 26 Funnel plot for all-cause mortality for RIV in VTE, Scenario 2: only vs VKA±Hep (trim and fill)


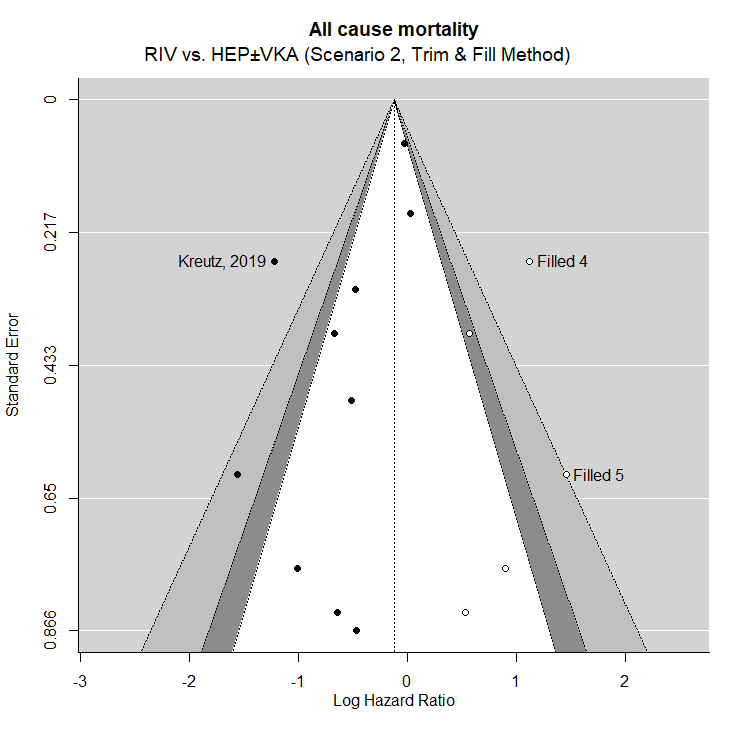


Hazard Ratio [95% CI] (Trim & Fill Method): 0.88 [0.61, 1.28]

Following imputation of ‘missing’ studies using trim & fill method added until reaching the symmetry of funnel plot, the new overall hazard ratio was estimated.

Supplementary figure 27 Funnel plot for major bleeding for RIV in VTE, base scenario


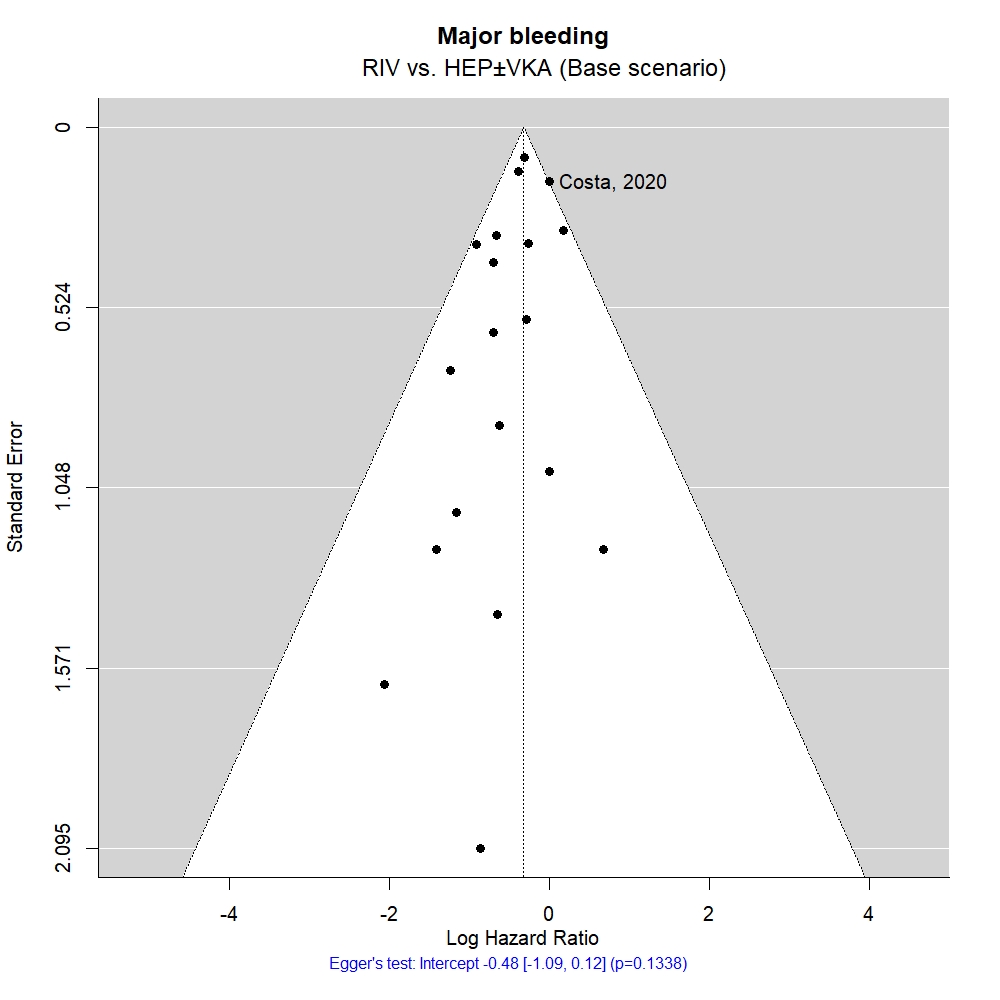


Supplementary figure 28 Funnel plot for major bleeding for RIV in VTE, Scenario 2: only vs VKA±Hep


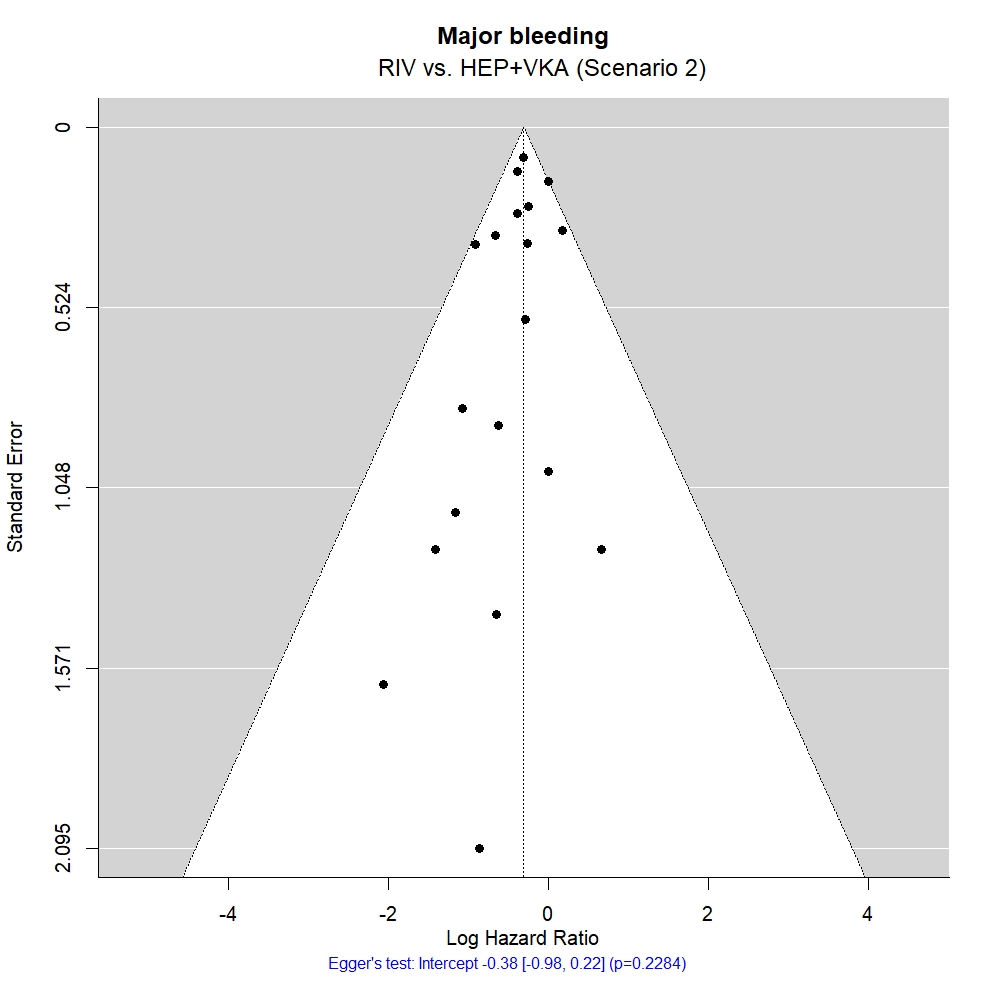


Supplementary figure 29 Funnel plot for major bleeding for RIV in VTE, Scenario 3: only vs VKA+Hep


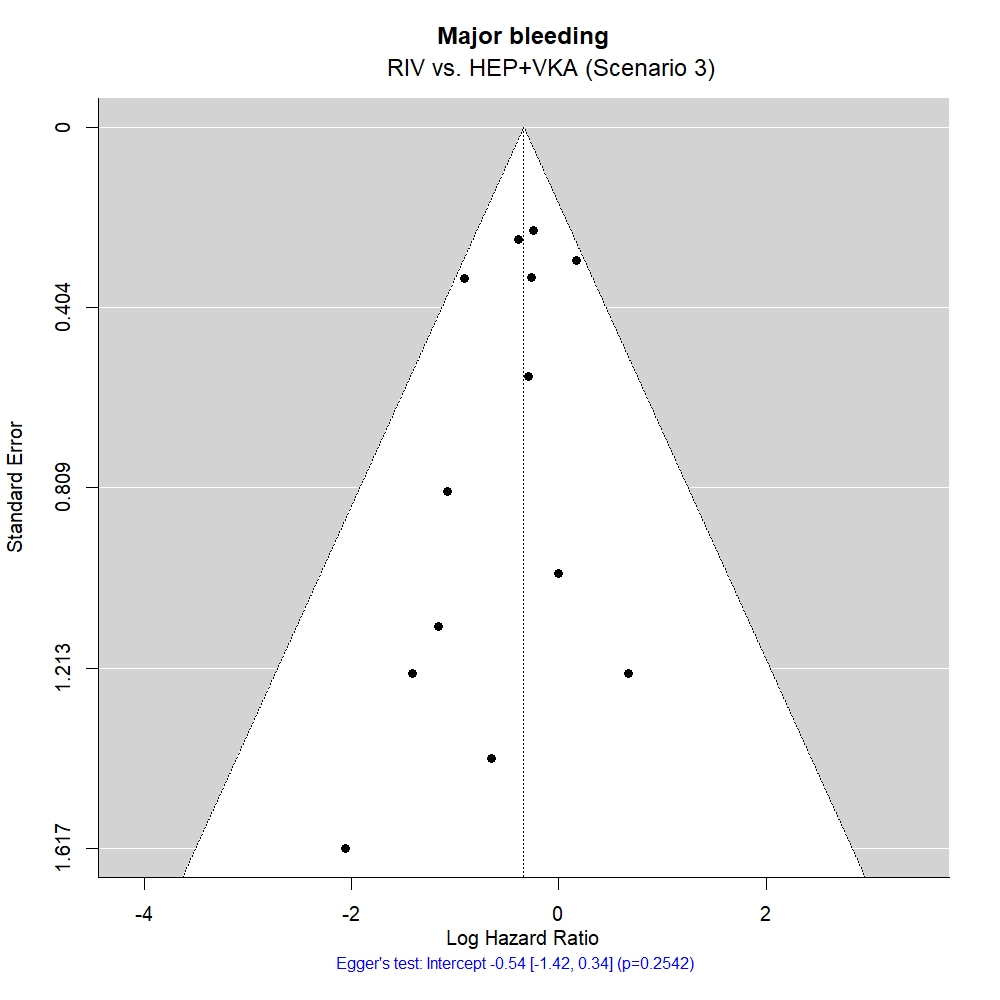


Supplementary figure 30 Funnel plot for major bleeding for RIV in VTE, Scenario 4: ≤6 months treatment


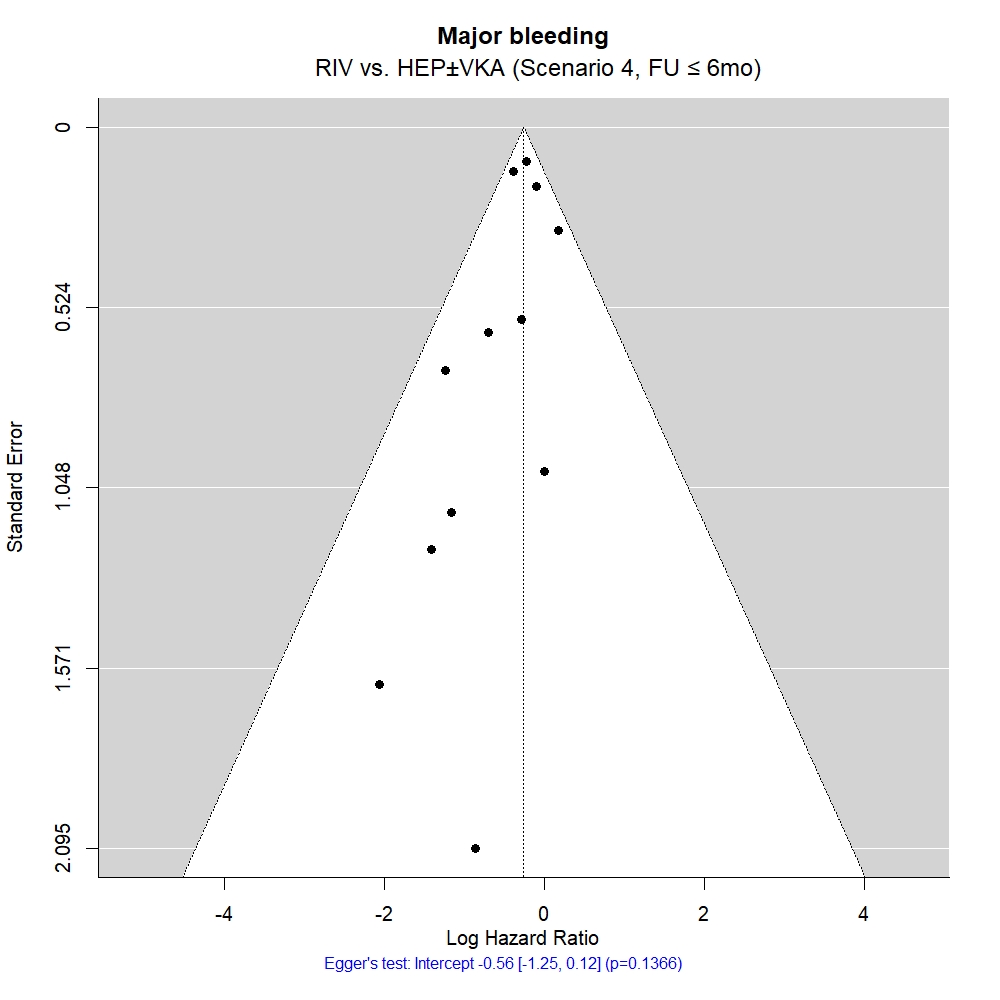


**References**

1. Ageno W, Mantovani LG, Haas S, Kreutz R, Monje D, Schneider J, et al. Safety and effectiveness of oral rivaroxaban versus standard anticoagulation for the treatment of symptomatic deep-vein thrombosis (XALIA): an international, prospective, non-interventional study. The Lancet Haematology.3(1):e12-e21.

2. Badreldin H. Hospital length of stay in patients initiated on direct oral anticoagulants versus warfarin for venous thromboembolism: a real-world single-center study. Journal of Thrombosis and Thrombolysis.46(1):16-21.

3. Bouget J, Balusson F, Maignan M, Pavageau L, Roy PM, Lacut K, et al. Major bleeding risk associated with oral anticoagulant in real clinical practice. A multicentre 3-year period population-based prospective cohort study. British Journal of Clinical Pharmacology. 2020;86(12):2519-29.

4. Bounameaux H, Haas S, Farjat AE, Ageno W, Weitz JI, Goldhaber SZ, et al. Comparative effectiveness of oral anticoagulants in venous thromboembolism: GARFIELD-VTE. Thrombosis Research. 2020;191:103-12.

5. Bryk AH, Pirog M, Plens K, Undas A. Heavy menstrual bleeding in women treated with rivaroxaban and vitamin K antagonists and the risk of recurrent venous thromboembolism. Vascular Pharmacology. 2016;87:242-7.

6. Bui MH, Son NT, Viet PT, Hiep NH, Chu Dinh T. Oral rivaroxaban versus standard therapy in acute venous thromboembolism treatment for vietnamese patients. Open Access Macedonian Journal of Medical Sciences.7(24):4255-9.

7. Carroll R, Lambrelli D, Donaldson R, Schultze A, Nordstrom B, Stynes G, et al. Treatment patterns of patients with venous thromboembolism treated with oral anticoagulants in England. Value in Health. 2018;21(Supplement 3):S115.

8. Chaudhari H, Lende V, Bojja N. [PC158] Comparative Study Between Rivaroxaban and Standard Therapy (Enoxaparin Followed by Acitrom [Vitamin K Antagonist]) for Treatment of Deep Venous Thrombosis. Journal of Vascular Surgery. 2019;69(6):e247.

9. Chu A, Limberg J. Rivaroxaban program for acute venous thromboembolism upon ED discharge, with focus on utility of commercially available dose pack. American Journal of Emergency Medicine. 2017;35(12):1910-4.

10. Coleman CI, Bunz TJ, Turpie AGG. Effectiveness and safety of rivaroxaban versus warfarin for treatment and prevention of recurrence of venous thromboembolism. Thrombosis and Haemostasis.117(10):1841-7.

11. Coleman CI, Bunz TJ. Post-Thrombotic Syndrome in Patients Treated with Rivaroxaban or Warfarin for Venous Thromboembolism. Res Pract Thromb Haemost. 2017;1:948-9.

12. Coleman CI, Turpie AGG, Bunz TJ, Beyer-Westendorf J. Effectiveness and Safety of Rivaroxaban Versus Warfarin in Frail Patients with Venous Thromboembolism. The American journal of medicine. 2018;131(8):933-8.e1.

13. Coleman CI, Turpie AGG, Bunz TJ, Beyer-Westendorf J. Effectiveness and safety of rivaroxaban versus warfarin in patients with provoked venous thromboembolism. Journal of Thrombosis and Thrombolysis.46(3):339-45.

14. Coleman CI, Turpie AGG, Bunz TJ, Baker WL, Beyer-Westendorf J. Effectiveness and safety of outpatient rivaroxaban versus warfarin for treatment of venous thromboembolism in patients with a known primary hypercoagulable state. Thrombosis Research. 2018;163:132-7.

15. Coleman CI, Turpie AG, Bunz TJ, Beyer-Westendorf J, Baker WL. Impact of prolonged anticoagulation with rivaroxaban on provoked venous thromboembolism recurrence: The improve-VTE study. Blood. 2018;132(Suppl. 1).

16. Coleman CI, Thomas JB, Peacock WF, Martinez BK, Baker WL. Effectiveness and safety of rivaroxaban versus warfarin in patients with unprovoked venous thromboembolism. THSNA 2018 Summit Abstract Proceedings; 2008: American Journal of Hematology; 2018. p. E12.

17. Costa OS, Beyer-Westendorf J, Ashton V, Milentijevic D, Moore KT, Bunz TJ, et al. Effectiveness and safety of rivaroxaban versus warfarin in obese patients with acute venous thromboembolism: analysis of electronic health record data. Journal of Thrombosis and Thrombolysis.51(2):349-58.

18. Costa OS, Thompson S, Ashton V, Palladino M, Bunz TJ, Coleman CI. Rivaroxaban versus warfarin for treatment and prevention of recurrence of venous thromboembolism in African American patients: a retrospective cohort analysis. Thrombosis J.18(1):6.

19. Dawwas G, Dietrich E, Park H. Comparative effectiveness of apixaban versus warfarin for the prevention of recurrent venous thromboembolism and cardiovascular diseases in patients with venous thromboembolism. Value in Health. 2018;21(Supplement 1):S57.

20. Dawwas GK, Smith SM, Dietrich E, Lo-Ciganic WH, Park H. Comparative effectiveness and safety of apixaban versus warfarin in patients with venous thromboembolism. American Journal of Health-System Pharmacy. 2020;77(3):188-95.

21. De Crem N, Peerlinck K, Vanassche T, Vanheule K, Debaveye B, Middeldorp S, et al. Abnormal uterine bleeding in VTE patients treated with rivaroxaban compared to Vitamin K antagonists. Thrombosis Research. 2015;136(4):749-53.

22. Desai A, Amishi D, Calixte R, Aparnath M, Hindenburg A, Salzman S, et al. Comparing Length of Stay Between Patients Taking Rivaroxaban and Conventional Anticoagulants for Treatment of Venous Thromboembolism. Lung. 2016;194(4):605-11.

23. Ferreira T, Huber SC, de Moraes Martinelli B, Junior AL, Menezes FH, Orsi FA, et al. Low prevalence of Post-thrombotic syndrome in patients treated with rivaroxaban. Vascular Pharmacology. 2020;124:106608.

24. Fung KP, Chan KH, Ng V, Tsui PT, You JHS. Health Economic Analysis of Rivaroxaban and Warfarin for Venous Thromboembolism Management in Chinese Patients. Cardiovascular Drugs and Therapy. 2019;33(3):331-7.

25. Gaertner S, Cordeanu EM, Nouri S, Faller AM, Frantz AS, Mirea C, et al. Rivaroxaban versus standard anticoagulation for symptomatic venous thromboembolism (REMOTEV observational study): Analysis of 6-month outcomes. International Journal of Cardiology. 2017;226:103-9.

26. Goldhaber S, Ageno W, Casella I, Chee KH, Schellong S, Singer DE, et al. Efficacy and safety of Dabigatran Etexilate versus Vitamin K Antagonist for treatment of Acute Venous Thromboembolism in routine clinical practice: recovery DVT/PE prospective global cohort study. Journal of the American College of Cardiology. 2020;75(11):2195.

27. Gollamudi J, Al-Kindi S, Martin P, Nayak LV. Safety of apixaban in patients with CKD stage v and ESRD with venous thromboembolism. Blood. 2018;132(Suppl. 1).

28. Guo JD, Rajpura J, Hlavacek P, Keshishian A, Sah J, Delinger R, et al. Comparative Clinical and Economic Outcomes Associated with Warfarin Versus Apixaban in the Treatment of Patients with Venous Thromboembolism in a Large U.S. Commercial Claims Database. Journal of managed care & specialty pharmacy. 2020:1-14.

29. Hlavacek P, Guo JD, Rosenblatt L, Keshishian A, Russ C, Mardekian J, et al. Safety, effectiveness, and health care cost comparisons among elderly patients with venous thromboembolism prescribed warfarin or apixaban in the United States Medicare population. Current Medical Research and Opinion.35(12):2043-51.

30. Huang Y, Duan L, He W, Hong C, Guo Y, Wang X, et al. Efficacy and Safety of Rivaroxaban versus Warfarin for the Treatment of Acute Pulmonary Embolism: A Real-World Study. Analytical Cellular Pathology. 2020;2020:6813492.

31. Kohn CG, Bunz TJ, Beyer-Westendorf J, Coleman CI. Comparative risk of major bleeding with rivaroxaban and warfarin: Population-based cohort study of unprovoked venous thromboembolism. European Journal of Haematology. 2019;102(2):143-9.

32. Kreutz R, Mantovani LG, Haas S, Monje D, Schneider J, Bugge JP, et al. XALIA-LEA: An observational study of venous thromboembolism treatment with rivaroxaban and standard anticoagulation in the Asia-Pacific, Eastern Europe, the Middle East, Africa and Latin America. Thrombosis Research. 2019;176:125-32.

33. Krivoshchekov EP, Migunov IA, Romanov VE. [Rivaroxaban as monotherapy in patients with venous thromboembolism]. Monoterapiya Rivaroksabanom venoznykh tromboembolicheskikh oslozhnenii. 2016(10):61-5.

34. Kucher N, Aujesky D, Beer JH, Mazzolai L, Baldi T, Banyai M, et al. Rivaroxaban for the treatment of venous thromboembolism: The SWIss Venous ThromboEmbolism Registry (SWIVTER). Thrombosis and Haemostasis. 2016;116(3):472-9.

35. Kushnir M, Choi Y, Eisenberg R, Rao D, Tolu S, Gao J, et al. Efficacy and safety of direct oral factor Xa inhibitors compared with warfarin in patients with morbid obesity: a single-centre, retrospective analysis of chart data. The Lancet Haematology.6(7):e359-e65.

36. Lai YF, Neo JK, Cheen MH, Kong MC, Tai BC, Ng HJ. Comparison of Medication Adherence and Treatment Persistence between New Oral Anticoagulant and Warfarin among Patients. Annals of the Academy of Medicine, Singapore. 2016;45(1):12-7.

37. Larsen TB, Skjoth F, Kjaeldgaard JN, Lip GYH, Nielsen PB, Sogaard M. Effectiveness and safety of rivaroxaban and warfarin in patients with unprovoked venous thromboembolism: a propensity-matched nationwide cohort study. The Lancet Haematology. 2017;4(5):e237-e44.

38. Lopez-Nunez JJ, Perez-Andres R, Di Micco P, Schellong S, Gomez-Cuervo C, Sahuquillo JC, et al. Direct Oral Anticoagulants or Standard Anticoagulant Therapy in Fragile Patients with Venous Thromboembolism. TH Open. 2019;3(1):E67-E76.

39. Lutsey PL, Zakai NA, MacLehose RF, Norby FL, Walker RF, Roetker NS, et al. Comparative effectiveness of direct oral anticoagulants and warfarin on risk of bleeding resulting in hospitalization among venous thromboembolism patients. Circulation. 2018;137(Supplement 1).

40. Lutsey PL, Zakai NA, MacLehose RF, Norby FL, Walker RF, Roetker NS, et al. Risk of hospitalised bleeding in comparisons of oral anticoagulant options for the primary treatment of venous thromboembolism. British Journal of Haematology. 2019;185(5):903-11.

41. Moustafa F, Pesavento R, di Micco P, González‐Martínez J, Quintavalla R, Peris ML, et al. Real‐life Use of Anticoagulants in Venous Thromboembolism With a Focus on Patients With Exclusion Criteria for Direct Oral Anticoagulants. Clin Pharmacol Ther. 2018;103(4):684-91.

42. Nagaoki Y, Aikata H, Daijyo K, Teraoka Y, Shinohara F, Nakamura Y, et al. Efficacy and safety of edoxaban for treatment of portal vein thrombosis following danaparoid sodium in patients with liver cirrhosis. Hepatology Research. 2018;48(1):51-8.

43. Naymagon L, Tremblay D, Zubizarreta N, Moshier E, Troy K, Schiano T, et al. The efficacy and safety of direct oral anticoagulants in noncirrhotic portal vein thrombosis. Blood Advances. 2020;4(4):655-66.

44. Outler DL, Patel SM, Elliott JB, Knauss MD, Akbashev MY. Evaluation of rivaroxaban prescription adherence rates compared to warfarin for venous thomboembolism at a large teaching hospital. Journal of Thrombosis and Thrombolysis. 2019;47(4):603.

45. Ouyang H, Khillan R, Yu NH, Preet M. The real world experience regarding the safety of rivaroxaban and apixaban in venous thromboembolism patients with high bleeding risk. Blood. 2019;134(Supplement 1).

46. Patel SM, Wang T, Outler DL, Elliott J, Knauss M, Peasah SK, et al. Low persistence to rivaroxaban or warfarin among patients with new venous thromboembolism at a safety net academic medical center. Journal of Thrombosis and Thrombolysis. 2020;49(2):287-93.

47. Perales IJ, San Agustin K, DeAngelo J, Campbell AM. Rivaroxaban Versus Warfarin for Stroke Prevention and Venous Thromboembolism Treatment in Extreme Obesity and High Body Weight. Annals of Pharmacotherapy.54(4):344-50.

48. Petrikov AS, Shoikhet IN, Dudin DV, Karbyshev IA. [Use of a thrombin inhibitor for treatment of deep vein thrombosis and pulmonary thromboembolism in patients with thrombophilia]. Primenenie ingibitora trombina dlia lecheniia tromboza glubokikh ven i tromboembolii legochnoi arterii u bol'nykh s trombofiliei. 2017;23(2):33-40.

49. Poli D, Antonucci E, Vignini E, Martinese L, Testa S, Simioni P, et al. Anticoagulation resumption after intracranial hemorrhage in patients treated with VKA and DOACs. European Journal of Internal Medicine. 2020;80:73-7.

50. Roetker NS, Lutsey PL, Zakai NA, Alonso A, Adam TJ, Maclehose RF. All-Cause Mortality Risk with Direct Oral Anticoagulants and Warfarin in the Primary Treatment of Venous Thromboembolism. Thrombosis and Haemostasis. 2018;118(9):1637-45.

51. Sebastian T, Hakki LO, Spirk D, Baumann FA, Periard D, Banyai M, et al. Rivaroxaban or vitamin-K antagonists following early endovascular thrombus removal and stent placement for acute iliofemoral deep vein thrombosis. Thrombosis Research. 2018;172:86-93.

52. Sena S, Bulent M, Derya K, Deniz K, Halil A, Okan E, et al. Real-life data of direct anticoagulant use, bleeding risk and venous thromboembolism recurrence in chronic thromboembolic pulmonary hypertension patients: an observational retrospective study. Pulmonary Circulation. 2020;10(1):2045894019873545.

53. Sharifi M, Vajo Z, Freeman W, Bay C, Sharifi M, Schwartz F. Transforming and Simplifying the Treatment of Pulmonary Embolism: "Safe Dose" Thrombolysis Plus New Oral Anticoagulants. Lung. 2015;193(3):369-74.

54. Sindet-Pedersen C, Pallisgaard JL, Staerk L, Gerds TA, Fosbol EL, Torp-Pedersen C, et al. Comparative safety and effectiveness of rivaroxaban versus VKAs in patients with venous thromboembolism: A Danish nationwide registry-based study. Thrombosis and Haemostasis. 2017;117(6):1182-91.

55. Sogaard M, Nielsen PB, Skjoth F, Kjaeldgaard JN, Coleman CI, Larsen TB. Rivaroxaban Versus Warfarin and Risk of Post-Thrombotic Syndrome Among Patients with Venous Thromboembolism. American Journal of Medicine. 2018;131(7):787.

56. Spyropoulos AC, Ashton V, Chen YW, Wu B, Peterson ED. Rivaroxaban versus warfarin treatment among morbidly obese patients with venous thromboembolism: Comparative effectiveness, safety, and costs. Thrombosis Research. 2019;182:159-66.

57. Trutyak RI. Anticoagulant therapy in patients, suffering idiopathic deep veins thrombosis. 2016(10):48-50.

58. Utne KK, Dahm A, Wik HS, Jelsness-Jorgensen LP, Sandset PM, Ghanima W. Rivaroxaban versus warfarin for the prevention of post-thrombotic syndrome. Thrombosis Research. 2018;163:6-11.

59. Wang L, Baser O, Wells P, Peacock WF, Coleman CI, Fermann GJ, et al. Overall Effectiveness of Rivaroxaban in Patients with Pulmonary Embolism. Clinical Therapeutics. 2017;39(7):1426-36.e2.

60. Weeda ER, Peacock WF, Fermann GJ, Baugh CW, Wells PS, Ashton V, et al. Shortened hospital length of stay and lower costs associated with rivaroxaban in patients with pulmonary embolism managed as observation status. International Journal of Clinical Practice. 2017;71(1):e12915.

61. Weycker D, Li X, Wygant G, Lee T, Hamilton M, Luo X, et al. Safety and effectiveness of apixaban versus warfarin in the treatment and prevention of venous thromboembolism. Journal of the American College of Cardiology. 2018;71(11 Supplement 1).

62. Weycker D, Li X, Wygant GD, Lee T, Hamilton M, Luo X, et al. Effectiveness and Safety of Apixaban versus Warfarin as Outpatient Treatment of Venous Thromboembolism in U.S. Clinical Practice. Thrombosis and Haemostasis. 2018;118(11):1951-61.

63. Wysokinski W, Bott-Kitslaar D, Casanegra A, Froehling D, Bartlett M, Lenz C, et al. Efficacy and Safety of Rivaroxaban and Apixaban in Relation to Low Molecular Heparin and Vitamin K Antagonists in Acute Venous Thromboembolism – A Single Center Prospective Study. Res Pract Thromb Haemost. 2018;2:335-6.

64. Yuko N, Aikata H, Murakami E, Nakahara T, Kawaoka T, Tsuge M, et al. Efficacy and safety of edoxaban for treatment of portal vein thrombosis in patients with liver cirrhosis. Hepatology. 2018;68(Supplement 1):1159A-60A.

65. Zakai N, Walker R, Maclehose R, Koh I, Alonso A, Lutsey P. Recurrence risk according to anticoagulant prescribed for the secondary prevention of venous thrombosis. Res Pract Thromb Haemost. 2019;3(Supplement 1):815.
